# Lack of adverse effects in subchronic and chronic toxicity/carcinogenicity studies on the glyphosate-resistant genetically modified maize NK603 in Wistar Han RCC rats

**DOI:** 10.1007/s00204-019-02400-1

**Published:** 2019-02-12

**Authors:** Pablo Steinberg, Hilko van der Voet, Paul W. Goedhart, Gijs Kleter, Esther J. Kok, Maria Pla, Anna Nadal, Dagmar Zeljenková, Radka Aláčová, Júlia Babincová, Eva Rollerová, Soňa Jaďuďová, Anton Kebis, Elena Szabova, Jana Tulinská, Aurélia Líšková, Melinda Takácsová, Miroslava Lehotská Mikušová, Zora Krivošíková, Armin Spök, Monica Racovita, Huib de Vriend, Roger Alison, Clare Alison, Wolfgang Baumgärtner, Kathrin Becker, Charlotte Lempp, Marion Schmicke, Dieter Schrenk, Annette Pöting, Joachim Schiemann, Ralf Wilhelm

**Affiliations:** 1 Institute for Food Toxicology, University of Veterinary Medicine Hannover, Bischofsholer Damm 15, 30173 Hannover, Germany; 2 Wageningen University and Research, Biometris, Droevendaalsesteeg 1, 6708 PB Wageningen, The Netherlands; 3 RIKILT Wageningen University & Research, Akkermaalsbos 2, 6708 WB Wageningen, The Netherlands; 4 Institute of Food and Agricultural Technology, University of Girona, Campus Montilivi, 17003 Girona, Spain; 5 CRAG-CSIC-IRTA-UAB-UB, Edifici CRAG, Campus UAB, 08193 Cerdanyola, Spain; 6 Slovak Medical University, Faculty of Public Health, Limbová 12, 83303 Bratislava, Slovakia; 7 Slovak Medical University, Faculty of Medicine, Limbová 12, 83303 Bratislava, Slovakia; 8 Graz University of Technology, Schlögelgasse 2, 8010 Graz, Austria; 9 Alpen-Adria Universität Klagenfurt, Schlögelgasse 2, 8010 Graz, Austria; 10 LIS Consult, Hogesteeg 9, 3972 JS Driebergen, The Netherlands; 11 Roger Alison Ltd., Lampeter, Ceredigion, UK; 12 Department of Pathology, University of Veterinary Medicine Hannover, Bünteweg 17, 30559 Hannover, Germany; 13 Clinic for Cattle, University of Veterinary Medicine Hannover, Bischofsholer Damm 15, 30173 Hannover, Germany; 14 Food Chemistry and Toxicology, University of Kaiserslautern, Erwin-Schrödinger-Straße 52, 67663 Kaiserslautern, Germany; 15 Federal Institute for Risk Assessment, Max-Dohrn-Straße 8-10, 10589 Berlin, Germany; 16 Institute for Biosafety in Plant Biotechnology, Julius Kühn-Institut (JKI), Federal Research Centre for Cultivated Plants, Erwin-Baur-Str. 27, 06484 Quedlinburg, Germany; 17 Present Address: Max Rubner-Institut, Federal Research Institute of Nutrition and Food, Haid-und-Neu-Str. 9, 76131 Karlsruhe, Germany; 18 Present Address: Global Sustainability Institute, Anglia Ruskin University, East Road, Cambridge, CB1 1PT UK

**Keywords:** Genetically modified maize NK603, GMO risk assessment, Biosafety, G-TwYST, OECD Test Guideline No. 408, OECD Test Guideline No. 453, Rat feeding trial, Subchronic oral toxicity study, Combined chronic toxicity/carcinogenicity study

## Abstract

In 2012, a controversial study on the long-term toxicity of a Roundup herbicide and the glyphosate-tolerant genetically modified (GM) maize NK603 was published. The EC-funded G-TwYST research consortium tested the potential subchronic and chronic toxicity as well as the carcinogenicity of the glyphosate-resistant genetically modified maize NK603 by performing two 90-day feeding trials, one with GM maize inclusion rates of 11 and 33% and one with inclusion rates of up to 50%, as well as a 2-year feeding trial with inclusion rates of 11 and 33% in male and female Wistar Han RCC rats by taking into account OECD Guidelines for the testing of chemicals and EFSA recommendations on the safety testing of whole-food/feed in laboratory animals. In all three trials, the NK603 maize, untreated and treated once with Roundup during its cultivation, and the conventional counterpart were tested. Differences between each test group and the control group were evaluated. Equivalence was assessed by comparing the observed difference to differences between non-GM reference groups in previous studies. In case of significant differences, whether the effects were dose-related and/or accompanied by changes in related parameters including histopathological findings was evaluated. It is concluded that no adverse effects related to the feeding of the NK603 maize cultivated with or without Roundup for up to 2 years were observed. Based on the outcome of the subchronic and combined chronic toxicity/carcinogenicity studies, recommendations on the scientific justification and added value of long-term feeding trials in the GM plant risk assessment process are presented.

**Electronic supplementary material:**

The online version of this article (10.1007/s00204-019-02400-1) contains supplementary material, which is available to authorized users.

## Introduction

Since the mid-1990s, genetically modified (GM) crops have increasingly been grown commercially around the world, reaching a global acreage of 189.8 million hectares planted by farmers worldwide, particularly in non-EU countries, such as USA, Brazil, Argentina, Canada, and India. The four major cultivated commodity crops are soybean, maize, oilseed rape, and cotton, whilst a wide range of other GM crops, fruits, and vegetables are grown to a lesser extent. The main traits that have been introduced through genetic modification are herbicide tolerance and insect resistance (ISAAA 2017).

In many countries around the world, GM crops are only allowed onto the market after a regulatory approval or consultation procedure, which entails, amongst others, a pre-market safety assessment. To assess the safety of foods derived from GM crops, the FAO/WHO Codex Alimentarius established internationally harmonized guidelines (Codex Alimentarius 2008). Central to the safety assessment is the comparison of a given GM crop with a genetically near non-GM counterpart with a history of safe use, focusing on the differences between both, identifying both intended and unintended effects of the genetic modification. This entails, amongst others, a comprehensive analysis of the compositional characteristics of these comparators, including nutrients (e.g. amino acids, fatty acids, vitamins and minerals), anti-nutrients, toxins, and other compounds of biological relevance (e.g. phytoestrogens). Based on the differences thus identified and the information already available on the possible safety implications of such changes, it can be determined if and how the further safety assessment should be performed before reaching a conclusion. For newly expressed proteins encoded by the introduced foreign genes, the assessment may, for example, address their potential toxicity and allergenicity through the performance of a number of in silico, in vitro or in vivo tests (Codex Alimentarius 2008).

Besides the above-mentioned compositional analysis, testing of whole food products may be considered in some exceptional cases, whilst it has to be realized that foods are complex mixtures and, unlike purified chemicals, there are boundaries to the dose ranges that can be tested in experimental animals, thereby diminishing the sensitivity of such tests (Codex Alimentarius 2008). In the EU, the requirements for the risk assessment of GM food/feed are specified in detail by the Implementing Regulation No. 503/2013 (EU 2013a), which takes into account the Codex Alimentarius guideline for the conduct of food safety assessment of foods derived from GM plants (Codex Alimentarius 2008).

According to the Implementing Regulation No. 503/2013, applicants are requested to carry out an obligatory 90-day feeding study with whole food/feed for each GMO event to be marketed in the EU, although various stakeholders presented scientific arguments not unanimously supporting this requirement (see e.g. Devos et al. 2016). Depending on the outcome of previous studies, a 2-year carcinogenicity study with rats may also be requested by the European Food Safety Authority (EFSA) on a case-by-case basis. To prepare for such eventualities, EFSA was asked by the European Commission to provide supplementary guidance on key elements to be considered for a 2-year carcinogenicity trial in rats with whole food/feed. Against this backdrop and to address possible concerns after the publication of a study on the long-term toxicity of Roundup, a glyphosate-based herbicide formulation and the glyphosate-tolerant genetically modified maize NK603 (Séralini et al. 2012; EFSA 2012), the European Commission funded the 4-year research project G-TwYST (GM Plant Two Year Safety Testing) to address the following issues related to 2-year feeding trials in a stepwise approach: (1) the execution of at least one rat feeding trial with the GM maize NK603 applying EFSA protocols and recommendations, whereby the participating institutions should strictly comply with all applicable international standards and norms concerning feeding trials in close collaboration with EFSA; (2) analysis, report and provision of recommendations, in particular as to the scientific justification and added value of such long-term feeding trials with regard to GMO risk assessment.

To achieve the first objective, the G-TwYST partners performed three rat feeding studies with GM maize NK603, both untreated and treated once with Roundup^®^ during its cultivation, as well as the untreated conventional counterpart as control:

- two 90-day trials for subchronic toxicity testing, one with GM maize inclusion rates of 11 and 33% and one with GM maize inclusion rates of 11, 33 and 50% as well as
- a combined chronic toxicity/carcinogenicity (2-year) study with GM maize inclusion rates of 11 and 33%,

all three of them based on the OECD Guidelines for the testing of chemicals (OECD 1998, 2009a) and EFSA recommendations (EFSA 2011a, 2013, 2014). A 90-day feeding trial with a maize incorporation rate of 50% was included, since EFSA (2014) proposed it as the reference value for a high maize dose in 90-day studies in rodents, based on a report by Zhu et al. (2013). Furthermore, various stakeholders strongly supported the performance of a 90-day feeding trial with a maize incorporation rate of 50% at the first G-TwYST Stakeholder Meeting held in Vienna, Austria, in December 2014.

In the present study, the results of the three above-mentioned feeding trials are described and discussed. Based on the results obtained, the conclusions and recommendations of the G-TwYST consortium on the design, conduct and analysis of rat feeding studies with whole food/feed, as well as the scientific justification and added value of long-term feeding trials for the GM plant risk assessment are presented.

## Materials and methods

### Plant material

Given that the NK603 maize had been approved in the EU for import, processing as well as food and feed uses but not for cultivation, the NK603 and control maize plants were cultivated according to good agricultural practice in an experimental field station of the University of Guelph (ON, Canada) in 2014. Two varieties were produced: the GM maize NK603 (Pioneer 8906 R), without and with a Roundup application during cultivation, and its near-isogenic non-GM comparator (Pioneer 8906). About 84,000 plants/ha were planted in May 2014, the non-GM seeds on the 13th of May 2014 and the GM seeds on the 14th of May 2014. Weeds were controlled by applying Primextra^®^ II Magnum^®^ (S-Metolachlor + Atrazine, 3.5 L/ha) on the 20th of May 2014 at all sites and Roundup Transorb^®^ HC (2.5 L/ha, 1.35 kg glyphosate [potassium salt]/ha) at the “Roundup site” on the 20th of June 2014. GM maize and non-GM maize were grown on a farm in a distance of about 1 km to avoid cross pollination. Glyphosate-treated and untreated fields were separated by a field road. Maize was harvested in November 2014 and kernels were removed from the cobs on-site by machine. They had grain moisture levels in the usual range and were dried in a biological dryer, kept below 60 °C, down to a moisture level of 13–14%.

### Diet preparation and analyses

Four tons of GM maize NK603, four tons of Roundup-treated GM maize NK603 and 7.8 tons of the near-isogenic non-GM comparator (maize kernels) were transported on the 14th of December 2014 to Germany (air freight) and stored in big bags in a commercial storage facility not storing other grains and under ambient climate conditions. No other plant material was stored at the facility. The quality of the kernels was analysed after import. The sampling of the big bags (i.e. kernels from the same variety and treatment) was performed according to Regulation 691/2013 (EU 2013b) by a representative of the Landesuntersuchungsanstalt für das Gesundheits- und Veterinärwesen Sachsen in January 2015.

In autumn 2015, an infestation with the Indian meal moth (*Plodia interpunctella*) was noticed and a fumigation with phosphine, which does not leave residues on/in the stored kernels, took place. The big bags were moved to another storage facility and stored under ambient climate conditions between November 2015 and April 2017 (storage conditions: 12.3 ± 6.2 °C, 64.6 ± 7.2% humidity, hourly recorded). In summer 2016, mice were viewed in the storing hall, so that the kernels were sieved and placed in rigid containers.

Kernels were shipped to ENVIGO/Mucedola srl (Milan, Italy), milled (mesh size: 1 mm) to prepare the feed. The formulation of the diets was calculated by the nutritionist of the company based on their standard feeds and ingredients for all feeding trials to provide a balanced nutrition. The feeds were isoproteic, isocaloric and adjusted to the dietary requirements of the rat strain Wistar Han RCC used in the feeding trials. Besides the milled maize, the formulation mainly consisted of other plant-derived ingredients, including wheat, wheat middlings, soybean meal, soybean oil and a rice protein concentrate, while it did not contain animal-derived ingredients (Supplementary Electronic Material, Table 1). Except for the GM maize, the other ingredients of the diets were supposed to be GM-free, but traces of GMO (traits) were frequently detected: this information is documented in the diet analyses file that can be accessed via the internet portal named CADIMA (Central Access Database for Impact Assessment of Crop Genetic Improvement Technologies; http://www.cadima.info). Traces of GMO (traits) were also detected in the commercially available seeds of control maize. The pellets were dried at a temperature of < 50 °C, coded in a blinded fashion and sent to the Slovak Medical University (Bratislava, Slovakia) for the feeding trials as vacuum-packed, γ-irradiated batches (irradiation dose = 25 kGy).

Diets were produced in six batches. A re-coded part of batch 3 of all diet groups was used for the 90-day feeding trial with a GM maize inclusion rate of 11 and 33% (complemented with the near-isogenic variety up to 33% maize in toto), whereas a separate batch of diets was prepared for the 90-day feeding trial with a GM maize inclusion rate of up to 50% (complemented with the near-isogenic variety up to 50% maize in toto). Maize and diet subsamples were retained at the animal feed producing facility (Mucedola srl) for analysis. Irradiated diet samples of > 1.5 kg each were sent to the Julius Kühn-Institut (Quedlinburg, Germany) and stored at − 80 °C. Batches 1, 3 and 5 were analysed. Diet samples for analyses were shipped to RIKILT Wageningen University and Research, where the feed pellets were milled and re-mixed. Subsamples of the milled material were analysed by RIKILT and for complementary analyses either dispatched to Covance (Madison, WI, USA) or SGS (Hamburg, GER). The parameters measured and the methods applied by each of the certified laboratories are listed in the diet analyses file (http://ww.cadima.info).

### Study design

The study design of the two 90-day feeding trials (Tables 1, 7) was based on the OECD Test Guideline 408 for the testing of chemicals (OECD 1998) and EFSA recommendations on the performance of 90-day rodent feeding trials with whole food/feed (EFSA 2011a, 2014). The study design of the combined chronic toxicity/carcinogenicity feeding trial (Table 11) was based on the OECD Test Guideline 453 (OECD 2009) and EFSA considerations on the applicability of OECD TG 453 to whole food/feed (EFSA 2013).

As recommended by EFSA (2011a), two animals of the same gender were housed per cage and the cage was taken as the experimental unit. In the 90-day feeding trial with a GM maize NK603 inclusion rate of 11 and 33%, as well as in the combined chronic toxicity/carcinogenicity study there were five feeding groups (Tables 1, 11) and in the 90-day feeding trial with a GM maize inclusion rate of up to 50% there were eight feeding groups (Table 7). The cages were organized in blocks of 5–8 cages, and the feeding groups were randomized within blocks, i.e. a completely randomized block design was applied in all three feeding trials. There were separate blocks with male and female rats. To keep the units within blocks as homogeneous as possible, the ten heaviest rats of each sex (based on the weights 48 h after arrival) were housed in the first block, the next ten heaviest rats of each sex were housed in block 2, and so on. Except for feed consumption, which was determined per cage, all other parameters were measured in individual animals.

### Rat feeding trials

The trials were performed in compliance with GLP in the experimental animal facility at the Department of Toxicology of the Slovak Medical University in Bratislava (Slovakia). Five-week-old male and female specific-pathogen-free Wistar Han RCC (RccHan™:WIST) rats were purchased from Envigo (San Pietro al Natisone, Italy). A large amount of histopathological data has been published on the three main strains of rat used in carcinogenicity studies, i.e. Wistar Han, Sprague–Dawley, and Fischer 344 (Weber 2017). It is well known that Fischer 344 rats are prone to develop spontaneous myeloid leukemia and Leydig cell tumours, while Sprague–Dawley rats show high incidence rates in the case of spontaneous pituitary gland adenomas and mammary gland neoplasms (Weber 2017). The Wistar Han rat strain was used to perform the three feeding trials described in this paper because it shows the lowest incidence of spontaneous tumours in most organs when compared to the Fischer 344 and Sprague–Dawley strains.

The feeding trials were started 1 week after delivery of the animals at the animal testing facility. A detailed examination of all animals to verify their health condition (see the section “Periodical health status observations”) was carried out just before the start of the feeding trials. Feed and water were supplied ad libitum; feed was changed once a week and water every day. Feed consumption was determined once weekly during the first 13 weeks, every 2 weeks thereafter and reported as the total amount of feed consumed by two animals in one cage per week or 2 weeks, respectively.

### Periodical health status observations

Rats were inspected twice daily for changes in skin, fur, eyes, mucous membranes, occurrence of secretions and excretions as well as activity level and change in behaviour. A detailed physical examination of each animal out of the cage was performed prior to the beginning of the feeding trials, on day 1, once weekly during the first 13 weeks and once monthly thereafter to identify changes in skin, fur, eyes, mucous membranes, occurrence of secretions and excretions, autonomic activity such as lacrimation, piloerection, pupil size, unusual respiratory patterns as well as activity level and change in behaviour. At the end of the feeding trials a functional assessment of changes in gait, posture and response to handling, as well as the presence of clonic or tonic movements or bizarre behaviour (self-mutilation, walking backwards) was carried out. Sensory reactivity to auditory, visual and proprioceptive stimuli was recorded. An ophthalmologic examination of both eyes of all animals in the conscious state was performed prior to the beginning of the feeding trials and 2 weeks before the end of the studies. The eyes and the peribulbar structures were examined macroscopically after pupillary dilatation induced by instillation of a 0.5% tropicamide solution. Each animal was weighed 48 h after its arrival at the experimental animal facility of the Slovak Medical University, on the randomization day (i.e. day − 1), on the first day of the feeding trials, once weekly during the first 13 weeks, once every 2 weeks thereafter and at the end of the studies.

### Haematology and clinical biochemistry analyses

In the case of the two 90-day feeding trials, blood samples from the tail vein of 16 males and 16 females per group after 16–18 h fasting were taken at the end of the study for the haematological analyses (with EDTA as anticoagulant) as well as for the clinical biochemistry analyses (without anticoagulant). In the case of the combined chronic toxicity/carcinogenicity study, at the end of months 3 and 6, blood samples from the tail vein of 40 males and 40 females per group after 16–18 h fasting were taken for the haematological and clinical biochemistry analyses in the same way as described above. At months 12 and 24, blood samples were taken from all animals in the different groups.

No later than 4 h after collection of the blood samples the following haematology parameters were measured by making use of a Sysmex K-4500 automated haematology analyser (Sysmex, Kobe, Japan): white blood cell count (WBC), red blood cell count (RBC), haemoglobin concentration (HGB), haematocrit (HCT), mean cell volume (MCV), mean corpuscular haemoglobin (MCH), mean corpuscular haemoglobin concentration (MCHC), platelet count (PLT) as well as the absolute (LYMA) and relative lymphocyte count (LYMR). For the differential leukocyte count, blood smears were stained with the May–Grunwald and Giemsa–Romanowski dyes and thereafter examined by light microscopy; the percentage lymphocytes, neutrophils, eosinophils, basophils and monocytes were determined by examining 200 cells.

The parameters alkaline phosphatase (ALP), alanine aminotransferase (ALT), aspartate aminotransferase (AST), albumin (ALB), total protein (TP), glucose (GLU), creatinine (CREA), urea (U), cholesterol (CHOL), triglycerides (TRG), calcium (Ca), chloride (Cl), potassium (K), sodium (Na) and phosphorus (P) were measured maximally 4 h after collection of the blood samples in serum with an Ortho Clinical Vitros^®^ 250 Chemistry System (Ortho-Clinical Diagnostics, Raritan, NJ, USA), whereas coagulation parameters were not determined.

### Estrous cycle monitoring

17β-Estradiol was measured in the female rats being in the estrus phase of the estrous cycle. To monitor estrous cycles in adult female rats, daily vaginal lavages were taken during 10 days to track the estrous cycles by vaginal cytology. Vaginal lavages were obtained daily between 08:00 and 09:00 a.m. and examined under a low-power light microscope. Lavages were collected by flushing the entrance of the vagina with physiological saline, and a Giemsa–Romanowski stain was used to visualize the cells by optical microscopy. The stage of the estrous cycle was determined based on the presence of leukocytes (metestrus, diestrus), nucleated epithelial cells (proestrus) and cornified epithelial cells (estrus) (Long and Evans 1922; Caligioni 2009). A female rat that showed a constant 4- or 5-day vaginal estrous cycle was regarded as an animal with a regular estrous cycle. An extended estrus was defined as exhibiting cornified cells with no leukocytes for three or more days, and an extended diestrus was defined as the presence of leukocytes for four or more days (Cooper and Goldman 1999).

### Hormone measurements

17β-Estradiol blood levels were measured using the Estradiol (rat) ELISA (EIA-5774) from DRG Instruments (Marburg, Germany). The intra assay coefficient of variation was 4.1%, calculated by measuring one rat sample 20 times in one test routine. The lower detection limit was < 2.0 pg/mL. The cross-reactivity reported by the manufacturer was 4.2% for estrone, 3.8% for 17β-estradiol-3-glucuronide and 3.6% for 17β-estradiol-3-sulphate. Testosterone blood levels were measured using the Testosterone (Rat/Mouse) ELISA (EIA-5179) from DRG Instruments. The intra assay coefficient of variation was 5.3%, calculated by measuring one rat sample 20 times in one test routine. The lower detection limit was < 0.2 ng/mL. The cross-reactivity reported by the manufacturer was 69.6% for dihydrotestosterone and 7.4% for dihydroxyandrosterone. T_3_ blood levels were measured using the Total T3 RIA Kit (IM 1699) from Beckman Coulter (Brea, CA, USA). The intra assay coefficient of variation was 9.9%, calculated by measuring one rat sample 20 times in one test routine. The lower detection limit was < 0.75 nmol/L. T_4_ blood levels were measured using the Total T4 RIA Kit (IM 1447) from Beckman Coulter. The intra assay coefficient of variation was 10.2%, calculated by measuring one rat sample 20 times in one test routine. The lower detection limit was < 26 nmol/L.

### Urinalysis

In the case of the two 90-day feeding trials, an analysis of urine of 16 males and 16 females per group was performed at the end of the study. In the combined chronic toxicity/carcinogenicity study, an analysis of urine of 20 male and 20 female rats per group was performed at months 3, 6, and 12; at month 24, urine samples from all surviving animals were analysed.

Urine was collected from each individual rat in metabolic cages for 16 h. The parameters total protein, glucose, ketone, leukocyte number, erythrocyte number, bilirubin, urobilinogen, nitrate, and pH were analyzed with Combur^10^Test^®^ UX test strips (Roche Diagnostics, Mannheim, Germany) and semi-quantitatively evaluated by reflectance photometry with a Urilux S analyzer (Roche Diagnostics). Osmolarity was measured with the Advanced^®^ Model 3300 micro-osmometer from Advanced Instruments (Norwood, MA, USA).

### Gross necropsy and histopathology

At the end of the study, rats were anaesthetized after a 16- to 18-h fasting period with 10 mg/kg bw xylazine and 75 mg/kg bw ketamine. Blood samples for the corresponding analyses were taken from the abdominal aorta. Thereafter, the successive necropsy of the thoracic cavity, the abdominal cavity, the genital organs and the head was performed. Moreover, the wet weight of the kidneys, spleen, liver, adrenal glands, heart, thymus, uterus, ovaries, testes, epididymides and brain of all animals was recorded. Organ samples were stored in neutrally buffered 10% formalin, except for the eyes and the male reproductive tissues, which were immersed in Bouin’s solution. The formalin-fixed tissue samples were washed, dehydrated and embedded in paraffin. Thereafter, 4-µm thick sections were stained with haematoxylin and eosin for the light microscopic examination of the tissue structure.

A complete microscopic examination of the brain (including cerebrum, cerebellum and medulla/pons), spinal cord (at the cervical, mid-thoracic and lumbar level), pituitary, thyroid, parathyroid, thymus, oesophagus, salivary glands, stomach, small and large intestines, gut-associated lymphoid tissue (GALT), liver, pancreas, kidneys, adrenals, spleen, heart, trachea and lungs, aorta, tongue, eyes, Harderian gland, lacrimal gland, ovaries, cervix, vagina, uterus, female mammary gland, prostate, testes, seminal vesicles and coagulating gland, epididymides, urinary bladder, mesenteric and mandibular lymph nodes, peripheral nerve (sciatic), skeletal muscle, femur, sternum with bone marrow, and skin was performed.

In-life and necropsy phases were conducted “blind” at the Slovak Medical University (Bratislava, Slovakia). The group allocation was revealed to the histology facility (Wolfgang Baumgärtner, University of Veterinary Medicine Hannover, Germany) before the start of tissue staining and slide preparations, and to the pathologist (Roger Alison Ltd., Lampeter, UK) before the start of the histopathological examination, i.e. histology and histopathology were “non-blind”.

Histology was conducted “non-blind” for operational reasons. If histology had been conducted “blind”, all slides from all animals would have had to be processed, incurring significant penalties in cost and time. “Non-blind” histology allowed processing of only control and high dose group animals, together with macroscopic abnormalities and potential target organs. As the histology laboratory was not involved in the generation of data, this was not considered to have impacted upon the scientific evaluation of the study.

Histopathology was conducted “non-blind” because the consensus of opinion among toxicologic pathologists is that “blind reading” during the initial evaluation of tissues can have a negative impact on both the time it takes to accomplish the microscopic evaluation as well as the quality of the information obtained from the study (Iatropoulos 1984; Newberne and de la Iglesia 1985; SOTP 1986; Prasse et al. 1986; Goodman 1988; House et al. 1992; Crissman et al. 2004). “Blind reading” makes the task of separating treatment-related changes from normal variation more difficult and may result in missing subtle lesions. Awareness of the treatment group assignment allows the pathologist to intensely focus the histopathologic evaluation and to find important, and sometimes subtle, differences between the tissues of treated and untreated animals. “Blind reading” is commonly reserved for targeted review of lesions once they have been identified at the primary evaluation, particularly in a “Pathology Working Group”. In addition, all major published data on background lesions of rats from toxicity and carcinogenicity studies has been derived from “non-blind reading”. Reading the present series of studies “blind” would have significantly decreased the comparability of the findings to published data.

The resulting tissue sections were stained by hematoxylin and eosin at the above-mentioned histology facility. Histopathology examinations were performed in compliance with the principles of Good Laboratory Practice (GLP). The PathData V. 6.2d2 computer system was used for the recording and reporting of histopathology findings. A peer review of the study was conducted at the Test Site by Dr. C. Gopinath, Consultant Toxicological Pathologist. The histological sections and raw data (final signed histopathology report) will be returned to the Study Director for archiving. Other data related to the histopathological evaluation of the study will be archived by the study pathologist for at least 10 years. No data will be discarded after this period without the consent of the Test Facility. In the present report, summary tables with all necropsy and histopathology findings are presented. The complete histopathology reports (Alison 2018a, b, c) can be accessed via the internet portal CADIMA (http://www.cadima.info).

The statistical analysis of the incidence of tumors in the combined chronic toxicity/carcinogenicity study was based on the principles outlined by Peto et al. (1980). Peto’s method corrects for longevity (and hence for the period of time at risk) and applies a statistical approach appropriate to the cause of death (“context of observation”). This statistical analysis was performed on all animals from both phases of the study together. Where appropriate, tumors were also grouped for analysis (McConnell et al. 1986). The full analysis is presented in the PathData Appendix in the corresponding histopathology report (Alison 2018c). Neoplasms were considered statistically significant according to the Peto statistical test at *p* ≤ 0.05 for rare tumors and at *p* ≤ 0.01 for common tumors (FDA 2001). These analyses were performed using the PathData software.

### Statistical analysis

This section provides a short description of the methods used for the statistical analyses presented in this paper, with more detailed descriptions of these and some additionally applied methods given in the Electronic Supplementary Material. A full description of all the statistical methods used and the results obtained in the three studies, except for the histopathology, is given in the statistical reports (Goedhart and van der Voet 2017, 2018a, b, c, d, e, f, g, h, i), which can be accessed via the internet. Statistical analyses were performed separately for the three studies, for males and females, and for the four time points (3, 6, 12 and 24 months) in the combined chronic toxicity/carcinogenicity study. Our main interest was to analyse the difference between each of the GM maize feeding groups and the control feeding groups with the same amount of maize.

#### Data preparation

All parameters except time of death and pH were transformed to the natural logarithmic scale and then averaged to the cage level. This implies that, rather than looking at differences between feeding group means, ratios between the GM feeds and the corresponding control feed are of interest. Since the endpoints uLeu, uHemogl and uKeton had zero values, half of the smallest positive value was added to these observations before taking the logarithm. Data from rats fed diets with non-GM maize in the EU-funded project GRACE (http://www.grace-fp7.eu) were used as historical control data to set equivalence limits according to the method of van der Voet et al. (2017). The GRACE data have been analysed before (Schmidt and Schmidtke 2014; Schmidt et al. 2015a, b, 2016, 2017; Zeljenková et al. 2014, 2016). More details of the data preparation including outlier detection are given in the Electronic Supplementary Material.

Summary tables of means and standard deviations (SDs), classified by the feeding groups, were prepared on the original non-transformed scale. These tables were obtained by first calculating cage means and then calculating the summary statistics. Extended tables with the number of observations and coefficients of variation (%) are included in the statistical reports (Goedhart and van der Voet 2017, 2018b, c, d, e, f). In the combined chronic toxicity/carcinogenicity study, when measurements were made after 3, 6, 12 and 24 months, the number of cages per feeding group was 35 for the body weights, 20 for the haematology, differential WBC and clinical biochemistry data and 10 for the urine data. The number of cages per feeding group in the two 90-day feeding trials was 8, except for the hormone data with six cages for males and eight cages for females.

#### Mortality after 2 years

Death events of animals were rare up to month 12, with at most 3 dead animals per group of 50. Therefore, only the mortality rates at 24 months were statistically analysed. Only the 50 animals per sex and feeding group that were part of the 2-year cohort were statistically analysed, because the other 20 animals were sacrificed after 1 year. Fitting a beta-binomial regression model (Williams 1982) by means of maximum likelihood to the number of dead animals in each cage as response variable revealed that the estimate of the beta-binomial over-dispersion parameter equals its bounded value of 0.0001 for both males and females. This indicates that there was no over-dispersion and, therefore, the ordinary logistic model (McCullagh and Nelder 1989) was used to analyse the number of dead animals per cage. After allowing for differences between blocks, one-sided pairwise Wald tests were performed for the one-sided null hypothesis that the mortality probability of a GM feed is equal to or smaller than the mortality probability of the non-GM control feed. Mortality was also analysed by survival analysis using the procedures KAPLANMEIER, RSTEST, RPROPORTIONAL and RPHFIT and direct programming in GenStat 18 (VSN International 2015).

#### Growth curves and feed consumption

For each individual rat, growth curves were fitted to the observed weights for restricted periods of time. For the data up to 13 weeks (3 months), an exponential growth curve $A+B~\exp ( - \gamma \;{\text{Week}})$ with growth rate $\gamma$ was fitted to the observed weights. For the data between weeks 13 and 27 (6 months), and separately for the data between weeks 27 and 52 (12 months), a simple linear regression, ${\text{Weight}}=\alpha +\beta ~\;{\text{Week}}$, was fitted to the observed weights, and the growth rate was defined as $\gamma =\log (\beta )$. In general, the growth curves fitted very well and it was, therefore, decided to only analyse the estimated growth rates $\gamma$, further called growth rate, and the final weights observed after week 13, 27 and 52. The latter are further called Weight_13 (or just BodyWeight in the two 90-day feeding trials), Weight_27 and Weight_52. No general growth curves could be fitted for the data between weeks 53 and 104 (24 months) in the combined chronic toxicity/carcinogenicity study, and, therefore, only the final weights for those animals that survived for 24 months, further called Weight_104, were statistically analysed.

#### Equivalence and difference tests of quantitative endpoints

Traditionally, the first step in the statistical analysis of toxicological data is to determine if there are statistically significant differences between groups (difference tests). Only in a second step of the classical approach, the toxicological relevance of such statistically significant differences is interpreted in the light of historical control data. In this paper, the “toxicological relevance” question is directly addressed using equivalence tests. This procedure has a known probability of a Type 1 error (failing to find a potential relevant difference). In contrast, the traditional two-step approach has an unknown probability of failing to find a potential relevant difference because such a difference might not be statistically significant due to lack of precision. Thus, the one-step approach, which always considers the historical control data (irrespective of the result of the difference test), is preferred to the two-step approach. This is also in line with the EFSA recommendation (EFSA 2011c) stating that less emphasis should be placed on the reporting of statistical significance and more on statistical point estimation and associated interval estimations as more information can be presented using the latter.

Equivalence testing was introduced for GM safety assessment for compositional data in the EFSA guidance for risk assessment of food and feed from GM plants (EFSA 2011b). In the context of 90-day feeding studies in rodents, EFSA (2014) recognized the potential advantages of equivalence testing and recommended further investigation. In response to this issue, an equivalence test was developed in the course of the G-TwYST project (see van der Voet et al. [2017] for a full description and explanation of the approach). This test compares the difference between a test (T) and a control (C) feed, obtained simultaneously in a current study, to the typical differences between reference (R) feeds obtained in one or more historical studies. The equivalence test corrects for between-study differences, and the within-study variation between references R, along with the residual variation, is used to set equivalence limits for the difference between T and C in the current study. The so-called distribution wise equivalence (DWE) criterion is used in this test. The equivalence test employs the concept of desired power (here chosen as 95%) in a simplified situation, where there is no between-reference variation, where the historical and current studies have the same residual variance, and where the current study is assumed to have a sample size as approved by a regulator. Here we assumed regulatory sample sizes equal to the replication, i.e. the number of cages for most parameters in the current studies, e.g. 8 for the 90-day studies.

A critical factor is that the equivalence test of van der Voet et al. (2017) requires historical control data. It is assumed that the natural variability in results between non-GM reference groups can be estimated from previous studies in the same experimental facility. For the 3-month data in the three feeding trials, the GRACE non-GM reference data described in section “Data preparation” serve this purpose. For the data obtained after 6, 12 and 24 months, no corresponding reference data were available. Variance components between and within reference feeds were not much different between 3 and 6 months in the 90-day feeding trial with the GM maize at an inclusion rate of 11 and 33%, and, therefore, the 3-month GRACE data were still used as a reference for the 6-month data. The data variation was generally larger after 12 and 24 months; therefore, no equivalence tests are reported here for these data, and the assessment of the biological relevance was in these cases based on the expert opinion of the study coordinator. Details of the equivalence test calculations are given in the Electronic Supplementary Material.

The equivalence test results in a confidence interval for the so-called equivalence limit scaled difference (ELSD), which can be used both for difference and for equivalence testing. The hypothesis of no difference is rejected in case the interval does not contain zero, while the non-equivalence hypothesis is rejected in favour of an “equivalence” conclusion when the interval fully lies inside the interval (− 1, 1). When the ELSD point estimate is between − 1 and + 1, but one or both of the confidence limits lie outside this interval, the conclusion, in the terminology of EFSA, is that “equivalence is more likely than not”. “Non-equivalence is more likely than not” when the ELSD point estimate is lower than − 1 or higher than + 1. As a further help for interpretation of the ELSD graphs, it can be noted that confidence intervals for ELSD will become wider when the within-group variation in the current study is higher than in the historical data. Such higher variation may be treatment-induced or just the consequence of a lower analytical precision in the current study.

Classical statistical methods for continuous data were applied in line with OECD and EFSA approaches, and very similar to the approaches followed in the GRACE project (Schmidt and Schmidtke 2014; Schmidt et al. 2015a, b). OECD Test Guidelines require numerical results to be evaluated by an appropriate and acceptable statistical method, but give no further guidance on statistical analysis. More detailed guidance, although strictly only meant for chronic and carcinogenicity studies of single compounds, not of complex whole foods, is provided in chapter 4 of the OECD Guidance Document 116 (OECD 2014). A classical analysis of variance was performed on the cage means after log-transforming the data. This was done in the statistical program R. The analysis of variance was performed according to the randomized block design employing the model “Block + Treatment”, where Treatment defines the five feeding groups. For details see the Electronic Supplementary Material. Significance results of the different tests comparing GM maize-fed groups with the control group have been indicated in the tables with means and SDs.

All tests were performed by comparing two-sided 95% confidence intervals to values representing equivalence limits or strict equality (no difference).

### Stakeholder involvement and access to data

A key characteristic of the G-TwYST project was to allow for the involvement of a broad range of stakeholders and to ensure transparency of the research conducted, including access to the data. Stakeholder consultations were conducted not just on the results but also a priori on the study plans. The main stakeholder groups targeted were competent authorities, industry, civil society organizations, and researchers interested or experienced in animal feeding studies with GM food/feed. The geographical focus was on Europe.

In a first round, the draft study plans were subjected to a stakeholder consultation. The comments on these drafts, which had been received in the course of a stakeholder workshop and afterwards in writing, were discussed by the study team and taken into account when finalizing the study plans. As a result of the discussions during the stakeholder workshop, a 90-day feeding trial with a GM maize inclusion rate of up to 50%, as well as an analysis of hormone levels in blood samples of male (testosterone, T_3_ and T_4_) and female rats (17β-estradiol, T_3_ and T_4_) fed the GM maize at inclusion rates of 11 and 33% were performed. Study team members answered the stakeholder comments and questions in a written form, so that the stakeholders could verify if and how their comments had been taken into consideration and understand the underlying reasons for doing so.

In a similar way, the draft results and conclusions were subjected to stakeholder scrutiny. This final round included three subsequent steps, one workshop and two rounds of written comments: one round of written comments focused on the results of the 90-day studies. The stakeholder workshop and another round of written comments focused on the combined chronic toxicity and carcinogenicity study. To facilitate the consultation process, the documents including all full-length statistical as well as histopathology draft reports and draft interpretations were made available to registered participants that had signed a Non-Disclosure Agreement. Again, all stakeholder comments were considered when finalizing interpretation and conclusions, and written responses were prepared by the study team.

Maximum transparency of the process was established by publishing on the project website all stakeholder comments, as well as the written responses of the study team members in comprehensive consultation reports alongside with the draft and revised study plan (http://www.g-twyst.eu).

For each consultation round more than 700 stakeholders were invited by e-mail. Stakeholder participants were not selected in any way: all interested stakeholder representatives could participate. Overall 70 stakeholders from 19 countries participated in one or more consultation stages. A total of 158 stakeholder comments were received in written form and responded to by the study team. In each consultation stage, representatives of all main stakeholder groups targeted were involved and actively contributed.

### Access to the raw data

In line with the G-TwYST transparency policy any interested person will have access to the raw data obtained in the frame of the G-TwYST project, including the clinical, ophthalmological, body weight, haematology, clinical biochemistry, organ weight, necropsy and histopathology data presented in this study, through the internet portal CADIMA (http://www.cadima.info).

## Results

### Feed composition analysis

The main components of the diets are listed in Table 1 of the Electronic Supplementary Material. The amount of soybean meal was restricted to a level of 5% in all diets, since soybean meal contains high amounts of isoflavones, which per se induce estrogenic effects (Eisenbrand 2008) and could, therefore, interfere with the outcome of the feeding trials. Soybean meal is also an important protein source; to compensate the lower delivery of proteins by soybean meal, a rice protein concentrate was included in all diets.

The compositional analysis of the diets used in the three feeding trials performed with the GM maize NK603 in the course of the G-TwYST project can be accessed via the internet portal CADIMA (http://www.cadima.info). Briefly, six batches of all diets were prepared and batches 1, 3 and 5 of all diets were analyzed. All batches showed similar levels of the proximates (ash, total carbohydrates, fat, protein), starch, fibres, amino acids, fatty acids, minerals, vitamins, sugars, anti-nutrients and other secondary metabolites, except for the phenylpropanoids caffeic acid, ferulic acid and *p*-coumaric acid as well as the B vitamin niacin and the isoflavones daidzin and glycitin, which were nominally higher in the batch 1 than in the batches 3 and 5 of all diets. These differences are due to the fact that Covance analysed the bound + free phenylpropanoids in batch 1, while SGS analysed the free phenylpropanoids in batches 3 and 5. Moreover, the levels of the B vitamin niacin and the isoflavones daidzin and glycitin were also higher in batch 1 than in batches 3 and 5, which is due to some differences in the methodology applied by the different labs involved. None of the detected differences were considered to affect the health of the rats in any way. The levels of the above-mentioned proximates and compounds in the separate batch of diets prepared for the 90-day feeding trial with a GM maize inclusion rate of up to 50% were similar to those measured in the batch 3 of all diets, which were used for the 90-day feeding trial with a GM maize inclusion rate of 11 and 33%.

Low and similar amounts of polychlorinated dibenzo-*p*-dioxins and dibenzofurans, polychlorinated biphenyls, polycyclic aromatic hydrocarbons, mycotoxins and nitrosamines were detected in all analyzed diet batches. Residues of the pesticides 2-phenylphenol, cypermethrin, deltamethrin, tetramethrin, ethoxyquin, piperonyl butoxide, pirimiphos-methyl, *N*-desethyl-pirimiphos-methyl and propiconazole were detected in all diets. The glyphosate levels in the near-isogenic non-GM maize (Pioneer 8906) kernels, as well as in the untreated and Roundup-treated GM maize NK603 (Pioneer 8906 R) kernels were ≤ 16 ppb, while the glyphosate levels ranged between about 30 and 140 ppb in batches 1, 3 and 5 of all diets, including the control diet (Table 3, Electronic Supplementary Material). Glyphosate levels were below the limit of detection in the 50% control, 11% NK603, 50% NK603, 11% NK603 + Roundup and 50% NK603 + Roundup diets, while glyphosate was detected in the 33% control and 33% NK603 + Roundup diets used for the 90-day feeding trial with the GM maize NK603 at an inclusion rate of up to 50% (Table 4, Electronic Supplementary Material). The amount of the major glyphosate metabolite aminomethylphosphonic acid (AMPA) in all diets was below the limit of quantification (< 100 ppb). The detected levels of contaminants and pesticide residues were well below regulatory limits and, therefore, none of them were considered to affect the health of the rats in any way.

As expected, the NK603 event was detected in all analyzed batches of the diets containing 11, 33 or 50% of the GM maize NK603 using a highly specific PCR assay amplifying a DNA fragment overlapping the transition between inserted and genomic maize DNA in the NK603 maize (Tables 3, 4, Electronic Supplementary Material). Moreover, the NK603 event was not detected in batch 1 of the control diet, whereas the batches 3 and 5 of the control diets as well as the separate control diet batch with a 50% maize inclusion rate contained non-quantifiable traces of the NK603 event (Tables 3, 4, Electronic Supplementary Material). These NK603 maize traces were considered not to influence the feeding trials in any way.

Following irradiation, microorganisms such as coliforms, *Enterobacteriaceae*, yeast and molds were not detected in the diets.

### 90-day feeding trial with the GM maize NK603 at an inclusion rate of 11 and 33% in the diets

**Fig. 1:**
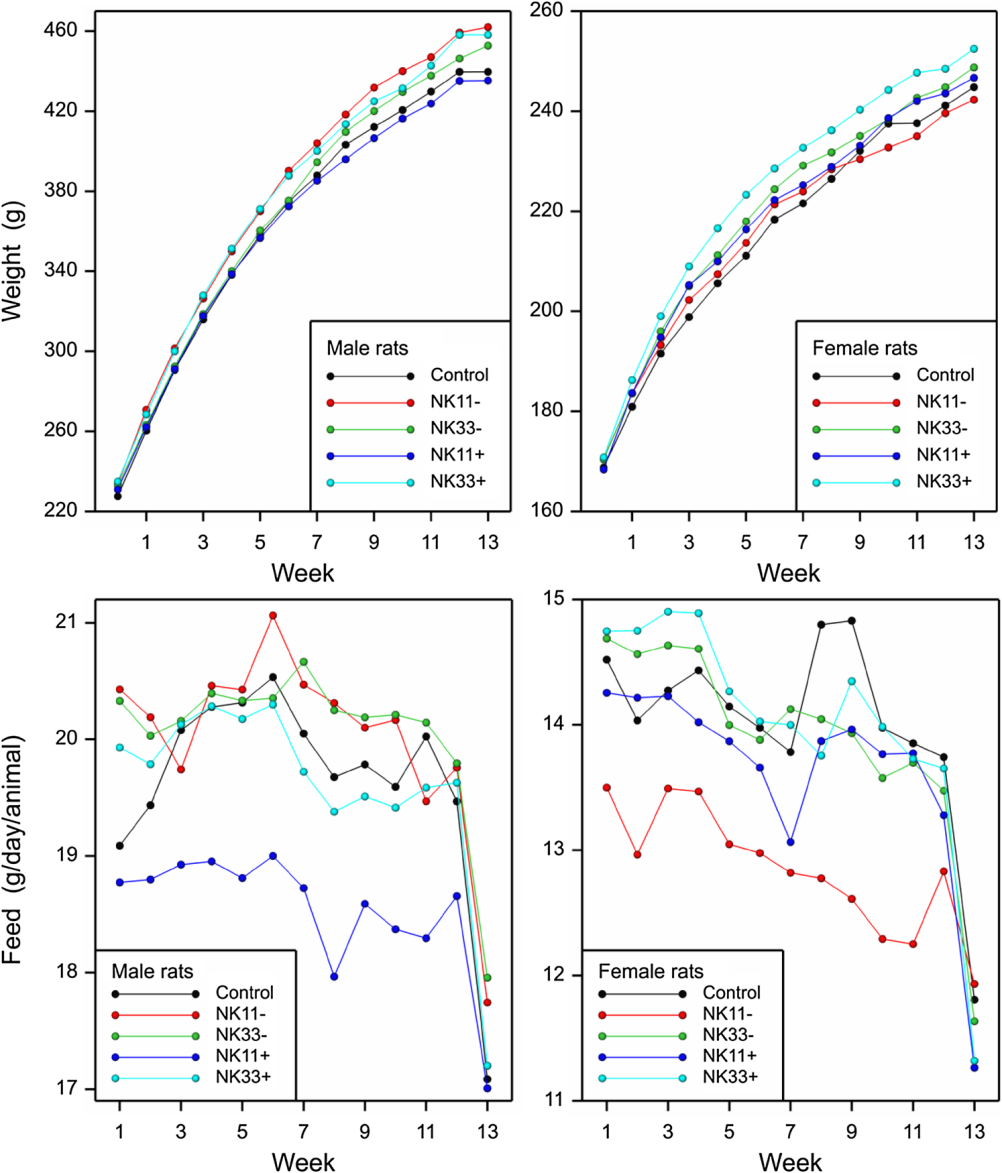
Male and female rat body weight development (upper charts) and feed consumption (lower charts) during the 90-day feeding trial with GM maize NK603 at an inclusion rate of 11 and 33% in the diet

**Fig. 2:**
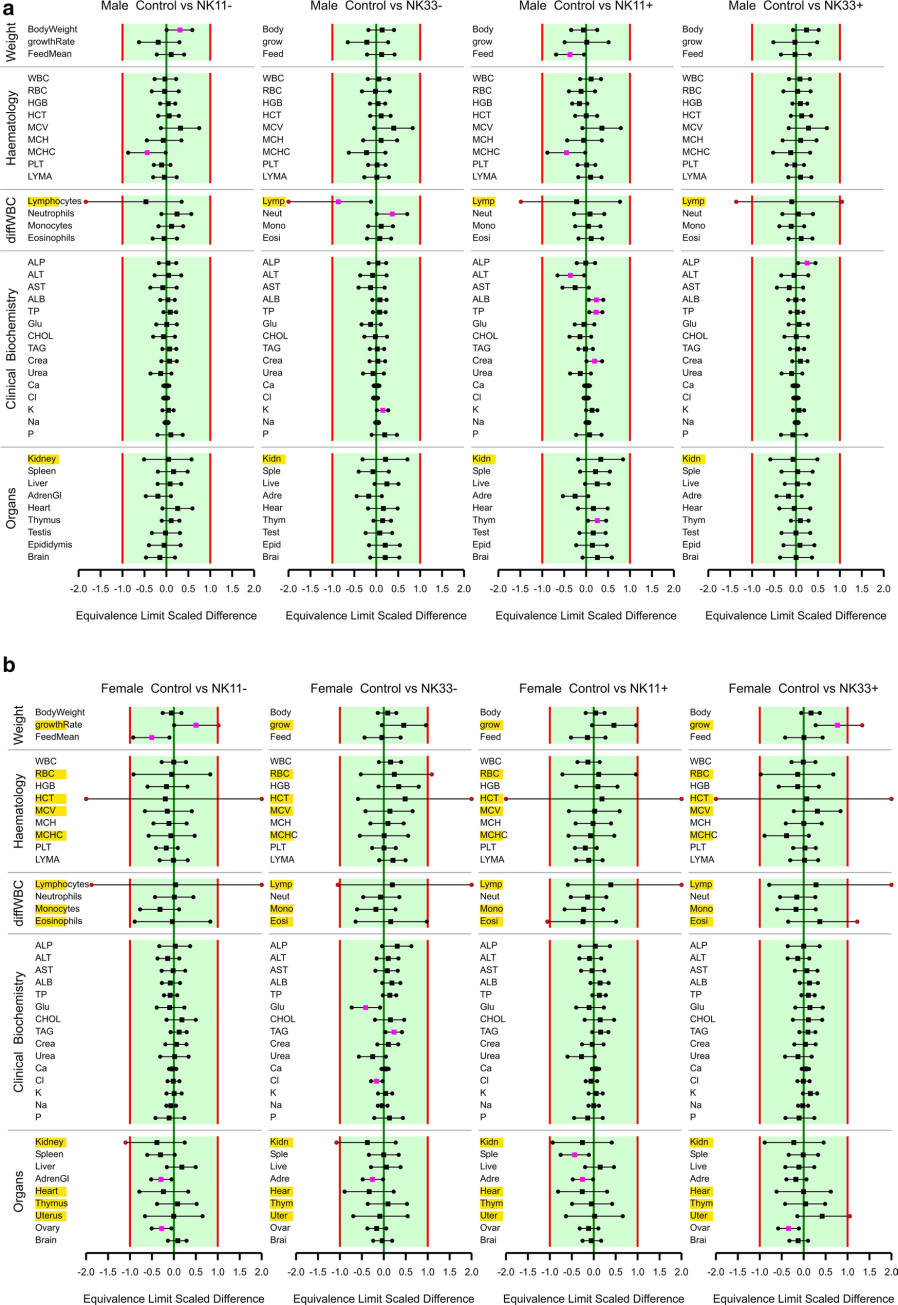
Equivalence testing of the parameters measured in male (**a**) and female rats (**b**) fed the diets with 11% GM maize NK603 (NK11−), 33% GM maize NK603 (NK33−), 11% GM maize NK603 + Roundup (NK11+) or 33% GM maize NK603 + Roundup (NK33+) versus the rats fed the control diet. For estimates (square symbols) left of zero, the parameter in rats fed the GM maize feed has a smaller mean than that in rats fed the control feed. Endpoints labelled with a yellow background have a larger residual variance compared to the historical studies (variance ratio > 150%). Fuchsia coloured symbols denote a significant difference

The study design is shown in Table 1. There was one unscheduled death: a male rat fed the 33% NK603 diet was sacrificed moribund on day 51 due to a malignant lymphoma, which was considered not to be treatment-related, since it was only observed in 1 out of 16 young animals having been fed the diet for less than 2 months. The rat body weights as well as the feed consumption during the 90-day feeding period in all five experimental groups are shown in Figs. 1 and 2. Statistically significant differences were observed in 5 of the 24 comparisons regarding final body weight, growth rate and feed consumption in male and female rats over four NK603 groups. The equivalence tests (in which the data obtained in the present study were compared with the historical control data of the animal testing facility, i.e. the data obtained from the GRACE project feeding trials) showed “equivalence” for 22 of the 24 comparisons, i.e. ELSD intervals were in the equivalence area between − 1 and + 1. The upper ELSD confidence limit was outside the interval [− 1, + 1] for the growth rate in female rats fed the 11% GM maize NK603 or the 33% GM maize NK603 + Roundup diet; therefore, in these two cases the appropriate conclusion is “equivalence more likely than not” (Fig. 2b).

**Table 1:** Study design of the 90-day feeding trial with the GM maize NK603 at an inclusion rate of 11 and 33% in the diet

Group	Content in the diet (%)			Number of animals	
	Near-isogenic non-GM	NK603	NK603 + roundup	Males	Females
1	33	0	0	16	16
2	22	11	0	16	16
3	0	33	0	16	16
4	22	0	11	16	16
5	0	0	33	16	16
Sentinels				6	6
Total				86	86

The data on the haematological parameters measured in the blood samples of the male and female rats are shown in Table 2 and Fig. 2. The MCHC was significantly lower in male rats fed the 11% NK603 diet and the 11% NK603 + Roundup (with a Roundup application during cultivation) diet when compared to the control value based on the 95% confidence interval. The percentage lymphocytes (determined in the differential leukocyte analysis) was significantly lower and the percentage neutrophils significantly higher in male rats fed the 33% NK603 diet. Apart from these four statistically significant differences in male rats, no significant differences were observed in the case of the other 108 comparisons with the control group involving the 14 haematological parameters in male and female rats over 4 NK603 groups. The equivalence tests showed “equivalence” in 89 of the 104 comparisons with the control group for 13 haematological parameters (note that there were no historical control data for 1 parameter, LYMR) in male and female rats over 4 NK603 groups, and “equivalence more likely than not” in the case of the remaining 15 comparisons. One or both ELSD confidence limits were outside the interval [− 1, + 1] for the percentage lymphocytes in the male rats fed with any of the 4 GM maize diets (Fig. 2a), for HCT and the percentage lymphocytes in the female rats fed with any of the 4 GM maize diets, for RBC in female rats fed the 33% NK603 diet and for the percentage eosinophils in female rats fed the 11% NK603 + Roundup and the 33% NK603 + Roundup diets (Fig. 2b). These were all cases in which the within-group data variance was larger than the within-variance of the historical control data.

**Table 2:** Haematology parameters (mean ± SD) in the serum of male and female Wistar Han RCC rats in the 90-day feeding trial with GM maize NK603 at an inclusion rate of 11 and 33% in the diet

Parameter	33% Control	11% NK603	33% NK603	11% NK603 + Roundup	33% NK603 + Roundup
Male rats					
WBC (10^9^/L)	9.10 ± 1.92	8.85 ± 1.58	9.42 ± 1.63	9.75 ± 1.22	9.57 ± 1.04
RBC (10^12^/L)	7.79 ± 0.30	7.77 ± 0.44	7.77 ± 0.27	7.67 ± 0.35	7.83 ± 0.20
HGB (g/dL)	14.6 ± 0.41	14.7 ± 0.29	14.7 ± 0.44	14.4 ± 0.55	14.8 ± 0.18
HCT (%)	42.4 ± 1.21	42.8 ± 1.96	43.0 ± 1.40	42.4 ± 1.60	43.2 ± 0.84
MCV (fL)	54.4 ± 1.07	55.2 ± 1.44	55.4 ± 1.16	55.4 ± 1.38	55.1 ± 0.63
MCH (pg)	18.8 ± 0.46	18.7 ± 0.52	19.0 ± 0.69	18.8 ± 0.78	19.0 ± 0.45
MCHC (g/dL)	34.6 ± 0.28	33.9 ± 0.94*	34.2 ± 0.78	33.9 ± 0.59*	34.4 ± 0.76
PLT (10^9^/L)	842 ± 75	792 ± 120	862 ± 125	851 ± 83	847 ± 115
LYMR (%)	73.0 ± 3.74	72.5 ± 4.97	70.9 ± 5.89	72.0 ± 3.41	73.4 ± 5.06
LYMA (10^3^/µL)	6.64 ± 1.55	6.42 ± 1.24	6.68 ± 1.34	7.00 ± 0.70	7.04 ± 1.09
Lymphocytes (%)	71.4 ± 6.45	68.1 ± 6.48	65.8 ± 5.86*	69.8 ± 6.53	70.6 ± 6.28
Neutrophils (%)	25.7 ± 6.58	28.8 ± 7.01	30.5 ± 5.17*	26.8 ± 6.83	26.4 ± 6.60
Monocytes (%)	1.50 ± 0.46	1.66 ± 0.44	1.78 ± 0.92	1.53 ± 0.41	1.31 ± 0.42
Eosinophils (%)	1.44 ± 0.58	1.41 ± 0.72	1.84 ± 1.32	1.91 ± 0.90	1.72 ± 0.78
Female rats					
WBC (10^9^/L)	5.52 ± 1.48	5.37 ± 0.97	6.06 ± 1.16	4.86 ± 0.70	5.43 ± 1.26
RBC (10^12^/L)	6.73 ± 0.22	6.72 ± 0.55	6.87 ± 0.17	6.81 ± 0.50	6.66 ± 0.30
HGB (g/dL)	13.7 ± 0.45	13.6 ± 1.03	14.1 ± 0.18	13.8 ± 0.81	13.6 ± 0.65
HCT (%)	39.4 ± 1.09	39.1 ± 3.29	40.5 ± 0.95	39.9 ± 2.57	39.6 ± 1.81
MCV (fL)	58.7 ± 1.40	58.2 ± 1.79	59.1 ± 1.03	58.7 ± 1.63	59.6 ± 1.32
MCH (pg)	20.4 ± 0.69	20.2 ± 0.54	20.6 ± 0.44	20.4 ± 0.84	20.4 ± 0.62
MCHC (g/dL)	34.8 ± 0.83	34.7 ± 0.75	34.8 ± 0.64	34.7 ± 0.93	34.3 ± 0.47
PLT (10^9^/L)	902 ± 113	825 ± 144	901 ± 91	813 ± 100	904 ± 94
LYMR (%)	75.2 ± 5.81	75.1 ± 3.52	77.5 ± 3.52	76.6 ± 4.92	77.1 ± 4.96
LYMA (10^3^/µL)	4.22 ± 1.37	4.04 ± 0.69	4.69 ± 0.91	3.71 ± 0.69	4.15 ± 0.97
Lymphocytes (%)	69.2 ± 5.98	69.5 ± 6.24	70.4 ± 3.79	71.9 ± 5.15	71.1 ± 4.08
Neutrophils (%)	28.1 ± 4.73	28.5 ± 5.76	26.8 ± 4.46	26.1 ± 5.02	26.1 ± 4.18
Monocytes (%)	1.53 ± 0.76	1.12 ± 0.65	1.44 ± 0.83	1.12 ± 0.38	1.25 ± 0.57
Eosinophils (%)	1.16 ± 1.20	0.91 ± 0.71	1.31 ± 0.94	0.88 ± 1.03	1.56 ± 0.98

Eight cages/group, two rats/cage

*Statistically significant difference to the control value based on the 95% confidence interval

The data on the clinical biochemical parameters measured in the blood samples of the male and female rats are shown in Table 3 and Fig. 2. In male rats, K levels were significantly higher in animals fed the 33% NK603 diet, ALT activity was significantly lower and ALB, TP, CREA and K levels were significantly higher in animals fed the 11% NK603 + Roundup diet and ALP activity was significantly higher in animals fed the 33% NK603 + Roundup diet when compared to the corresponding control diets. In female rats, GLU and Cl levels were significantly lower and the TAG level significantly higher if compared to the corresponding control values. Apart from these 10 significances, the other 110 comparisons of the 15 clinical biochemical parameters with the control group in male and female rats over four NK603 groups showed no statistically significant differences. The equivalence tests showed “equivalence” for all 120 comparisons involving clinical biochemical parameters, i.e. all ELSD intervals were fully inside the interval [− 1, + 1] (Fig. 2a, b).

**Table 3:** Clinical biochemistry parameters (mean ± SD) in the serum of male and female Wistar Han RCC rats in the 90-day feeding trial with GM maize NK603 at an inclusion rate of 11 and 33% in the diet^1^

Parameter	33% Control	11% NK603	33% NK603	11% NK603 + Roundup	33% NK603 + Roundup
Male rats					
ALP (µkat/L)	1.25 ± 0.14	1.28 ± 0.18	1.27 ± 0.14	1.25 ± 0.13	1.44 ± 0.19*
ALT (µkat/L)	0.56 ± 0.04	0.57 ± 0.06	0.55 ± 0.08	0.50 ± 0.06*	0.56 ± 0.08
AST (µkat/L)	2.40 ± 0.60	2.30 ± 0.47	2.23 ± 0.36	2.12 ± 0.38	2.22 ± 0.45
ALB (g/L)	36.4 ± 0.81	36.6 ± 1.77	36.9 ± 0.79	37.8 ± 0.99*	36.4 ± 1.30
TP (g/L)	64.4 ± 1.13	65.1 ± 2.32	65.0 ± 1.08	66.2 ± 1.66*	64.6 ± 1.36
GLU (mmol/L)	5.38 ± 0.85	5.34 ± 0.82	5.02 ± 0.69	5.15 ± 0.36	5.49 ± 0.53
CHOL (mmol/L)	2.05 ± 0.23	2.01 ± 0.27	2.02 ± 0.22	1.94 ± 0.19	2.04 ± 0.17
TAG (mmol/L)	1.02 ± 0.25	1.10 ± 0.14	1.05 ± 0.33	1.03 ± 0.31	1.09 ± 0.39
CREA (mmol/L)	40.1 ± 2.09	41.5 ± 4.78	40.8 ± 3.61	43.8 ± 4.66*	41.9 ± 4.15
UREA (mmol/L)	5.13 ± 0.32	4.90 ± 0.55	4.99 ± 0.28	4.88 ± 0.43	4.96 ± 0.50
Ca (mmol/L)	2.40 ± 0.04	2.40 ± 0.03	2.41 ± 0.06	2.41 ± 0.05	2.39 ± 0.04
Cl (mmol/L)	102 ± 1.33	102 ± 1.19	102 ± 1.03	102 ± 0.70	102 ± 1.22
K (mmol/L)	4.99 ± 0.23	5.09 ± 0.37	5.25 ± 0.20*	5.22 ± 0.31*	5.11 ± 0.24
Na (mmol/L)	145 ± 1.65	145 ± 1.46	145 ± 1.16	146 ± 1.12	145 ± 1.39
P (mmol/L)	2.37 ± 0.31	2.44 ± 0.19	2.50 ± 0.15	2.41 ± 0.16	2.31 ± 0.18
Female rats					
ALP (µkat/L)	0.58 ± 0.14	0.58 ± 0.06	0.65 ± 0.07	0.58 ± 0.07	0.58 ± 0.10
ALT (µkat/L)	0.47 ± 0.06	0.42 ± 0.04	0.53 ± 0.12	0.45 ± 0.13	0.42 ± 0.08
AST (µkat/L)	2.30 ± 0.67	2.22 ± 0.34	2.33 ± 0.25	2.21 ± 0.53	2.37 ± 0.44
ALB (g/L)	42.5 ± 3.53	41.5 ± 2.48	44.6 ± 2.71	44.1 ± 2.64	43.9 ± 2.84
TP (g/L)	68.7 ± 3.38	67.2 ± 2.90	71.0 ± 2.55	70.9 ± 2.41	70.5 ± 2.97
GLU (mmol/L)	5.19 ± 0.35	5.12 ± 1.18	4.40 ± 0.68*	5.05 ± 1.12	5.62 ± 1.03
CHOL (mmol/L)	1.91 ± 0.21	2.05 ± 0.28	2.01 ± 0.28	2.00 ± 0.25	1.99 ± 0.34
TAG (mmol/L)	0.52 ± 0.13	0.62 ± 0.20	0.65 ± 0.09*	0.60 ± 0.12	0.50 ± 0.39
CREA (mmol/L)	40.9 ± 2.43	42.0 ± 2.84	42.9 ± 3.98	40.8 ± 5.06	41.9 ± 5.67
UREA (mmol/L)	5.86 ± 0.35	5.94 ± 0.85	5.32 ± 0.67	5.29 ± 0.64	5.59 ± 0.57
Ca (mmol/L)	2.47 ± 0.07	2.45 ± 0.04	2.49 ± 0.04	2.50 ± 0.05	2.49 ± 0.05
Cl (mmol/L)	102 ± 1.03	102 ± 1.54	101 ± 1.06*	102 ± 1.15	102 ± 1.88
K (mmol/L)	4.45 ± 0.22	4.46 ± 0.20	4.52 ± 0.29	4.54 ± 0.21	4.70 ± 0.33
Na (mmol/L)	145 ± 1.94	144 ± 1.22	145 ± 1.16	145 ± 1.79	145 ± 2.68
P (mmol/L)	2.04 ± 0.23	1.96 ± 0.23	2.15 ± 0.36	1.93 ± 0.21	1.96 ± 0.22

Eight cages/group, two rats/cage

*Statistically significant difference to the control value based on the 95% confidence interval

The testosterone, T_3_ and T_4_ levels in the serum of male rats are shown in Table 4. The testosterone and T_3_ levels in the four groups fed the NK603 diet were not significantly different from those in the corresponding control group, whereas the T_4_ level was significantly lower in the group fed the 33% NK603 diet if compared to the control group. The 17β-estradiol, T_3_ and T_4_ levels in the serum of female rats are shown in Table 5. The 17β-estradiol levels in the four groups fed the NK603 diet were significantly lower than that in the control group. The T_3_ levels in the four groups fed the NK603 diet were not significantly different from that in the control group, while the T_4_ level was significantly lower in the 33% NK603 + Roundup group than in the control group.

**Table 4:** Testosterone, T_3_ and T_4_ levels (mean ± SD) in the serum of male Wistar Han RCC rats in the 90-day feeding trial with GM maize NK603 at an inclusion rate of 11 and 33% in the diet

Parameter	33% Control	11% NK603	33% NK603	11% NK603 + Roundup	33% NK603 + Roundup
Male rats					
Testosterone (ng/mL)	2.58 ± 0.88	2.78 ± 1.64	1.97 ± 1.12	3.03 ± 1.93	2.17 ± 0.56
T_3_ (nmol/L)	0.75 ± 0.06	0.82 ± 0.14	0.74 ± 0.09	0.75 ± 0.09	0.81 ± 0.08
T_4_ (nmol/L)	55.2 ± 4.74	54.0 ± 5.40	48.7 ± 3.65*	55.1 ± 3.77	53.4 ± 5.29

Six cages/group, two rats/cage

*Statistically significant difference to the control value based on the 95% confidence interval

**Table 5:** 17β-Estradiol, T_3_ and T_4_ levels (mean ± SD) in the serum of female Wistar Han RCC rats in the 90-day feeding trial with GM maize NK603 at an inclusion rate of 11 and 33% in the diet

Parameter	33% Control	11% NK603	33% NK603	11% NK603 + Roundup	33% NK603 + Roundup
Male rats					
17β-Estradiol (pg/mL)	7.74 ± 4.51	4.98 ± 3.45*	4.63 ± 1.35*	5.04 ± 2.08*	4.64 ± 1.29*
T_3_ (nmol/L)	0.76 ± 0.16	0.77 ± 0.12	0.69 ± 0.08	0.73 ± 0.13	0.63 ± 0.12
T_4_ (nmol/L)	34.2 ± 7.90	31.0 ± 8.50	34.0 ± 11.40	37.8 ± 9.00	25.4 ± 8.50*

Eight cages/group, two rats/cage

*Statistically significant difference to the control value based on the 95% confidence interval

The urinalysis data are summarized in the statistical report by Goedhart and van der Voet (2017). The urine pH was significantly lower in male rats fed the 33% NK603 and 33% NK603 + Roundup diets than in the control group, while the ketone level was significantly lower in female rats fed the 33% NK603 + Roundup diet than in the control group. Apart from these 3 significances, the other 45 comparisons with the control group involving six urinalysis parameters in male and female rats over 4 NK603 groups showed no significant differences.

The relative organ weights in male and female rats are shown in Table 6 and Fig. 2. The relative thymus weight in male rats fed the 11% NK603 + Roundup diet was significantly higher than that in the control group. In female rats, the relative adrenal gland weight in the groups fed the 11% NK603, 33% NK603 and the 11% NK603 + Roundup diets, the relative ovary weight in the animals fed the 11% NK603 and the 33% NK603 + Roundup diets, as well as the relative spleen weight in rats fed the 11% NK603 + Roundup diet were significantly lower when compared to the corresponding control groups. Apart from these 6 significances, the other 66 comparisons with the control group involving the 10 relative organ weights in male and female rats over 4 NK603 groups showed no significant differences. The equivalence tests showed that one or both ELSD confidence limits, but not the point estimates, were outside the interval [− 1, + 1] for the relative kidney weight in female rats fed the 11% NK603 and the 33% NK603 diets as well as for the relative uterus weight in the animals fed the 33% NK603 + Roundup diet (Fig. 2a, b). In these three cases, the conclusion is, therefore, “equivalence more likely than not”, whereas in the case of all other 69 comparisons with the control group involving the 10 organ weight parameters in male and female rats over three NK603 groups the conclusion is “equivalence”.

**Table 6:** Relative organ weights in male and female Wistar Han RCC rats in the 90-day feeding trial with GM maize NK603 at an inclusion rate of 11 and 33% in the diet

Organ	Relative organ weights (organ weight/body weight × 100)				
	33% Control	11% NK603	33% NK603	11% NK603 + Roundup	33% NK603 + Roundup
Male rats					
Adrenal glands	0.014 ± 0.001	0.013 ± 0.002	0.013 ± 0.002	0.013 ± 0.002	0.013 ± 0.001
Brain	0.49 ± 0.04	0.48 ± 0.03	0.51 ± 0.02	0.51 ± 0.03	0.49 ± 0.05
Epididymides	0.27 ± 0.02	0.26 ± 0.01	0.28 ± 0.02	0.27 ± 0.02	0.27 ± 0.03
Heart	0.23 ± 0.01	0.24 ± 0.01	0.24 ± 0.01	0.24 ± 0.02	0.23 ± 0.01
Kidneys	0.52 ± 0.04	0.52 ± 0.03	0.54 ± 0.03	0.55 ± 0.04	0.52 ± 0.04
Liver	2.16 ± 0.10	2.19 ± 0.13	2.24 ± 0.13	2.24 ± 0.10	2.17 ± 0.04
Spleen	0.18 ± 0.02	0.19 ± 0.01	0.18 ± 0.02	0.19 ± 0.02	0.18 ± 0.02
Testes	0.81 ± 0.08	0.80 ± 0.05	0.82 ± 0.04	0.83 ± 0.81	0.68 ± 0.06
Thymus	0.09 ± 0.01	0.10 ± 0.02	0.10 ± 0.02	0.10 ± 0.02*	0.10 ± 0.02
Female rats					
Adrenal glands	0.031 ± 0.003	0.028 ± 0.002*	0.028 ± 0.002*	0.028 ± 0.003*	0.029 ± 0.002
Brain	0.84 ± 0.06	0.85 ± 0.83	0.83 ± 0.05	0.83 ± 0.05	0.82 ± 0.02
Heart	0.32 ± 0.02	0.31 ± 0.04	0.31 ± 0.02	0.31 ± 0.02	0.32 ± 0.03
Kidneys	0.60 ± 0.05	0.57 ± 0.05	0.57 ± 0.05	0.58 ± 0.03	0.58 ± 0.03
Liver	2.44 ± 0.14	2.55 ± 0.19	2.50 ± 0.32	2.53 ± 0.17	2.39 ± 0.08
Ovaries	0.038 ± 0.003	0.034 ± 0.003*	0.035 ± 0.006	0.036 ± 0.003	0.032 ± 0.001*
Spleen	0.24 ± 0.02	0.23 ± 0.03	0.24 ± 0.02	0.22 ± 0.02*	0.25 ± 0.02
Thymus	0.12 ± 0.02	0.12 ± 0.02	0.12 ± 0.03	0.12 ± 0.02	0.12 ± 0.02
Uterus	0.25 ± 0.05	0.26 ± 0.08	0.24 ± 0.05	0.25 ± 0.06	0.31 ± 0.05

*Statistically significant difference to the control value based on the 95% confidence interval

The summary tables with all necropsy findings observed in male and female rats are listed in the Supplementary Electronic Material’s Table 5. There were no treatment-related necropsy findings following the feeding of NK603 or NK603 + Roundup to rats for 90 days. The summary tables with all histopathological findings observed in male and female rats are listed in the Supplementary Electronic Material’s Tables 6 and 7, respectively. There were no treatment-related histopathological findings following the feeding of NK603 or NK603 + Roundup to rats for 90 days.

### 90-Day feeding trial with the GM maize NK603 at an inclusion rate of up to 50% in the diets

**Fig. 3:**
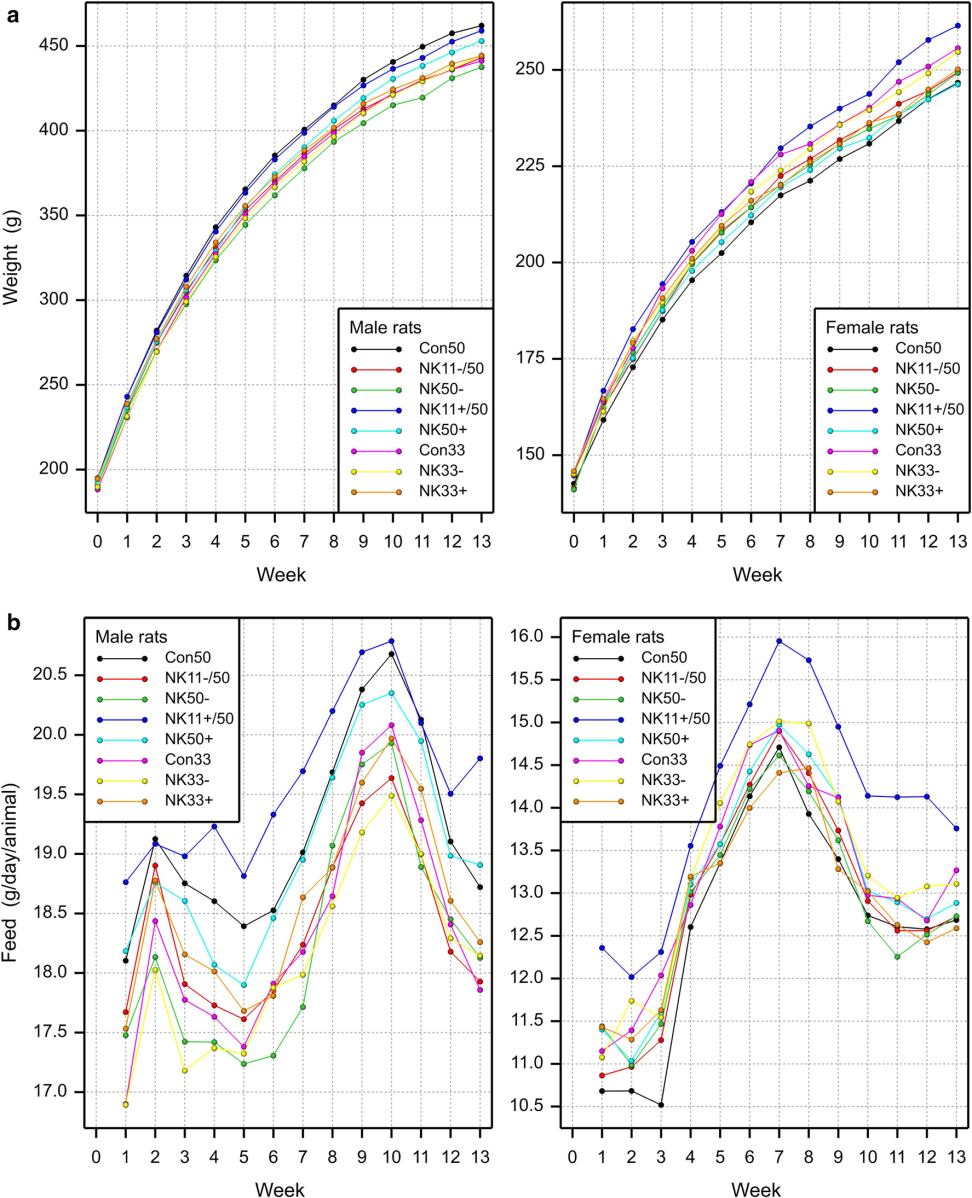
Male and female rat body weight development (upper charts) and feed consumption (lower charts) during the 90-day feeding trial with GM maize NK603 at an inclusion rate of up to 50% in the diet

**Fig. 4:**
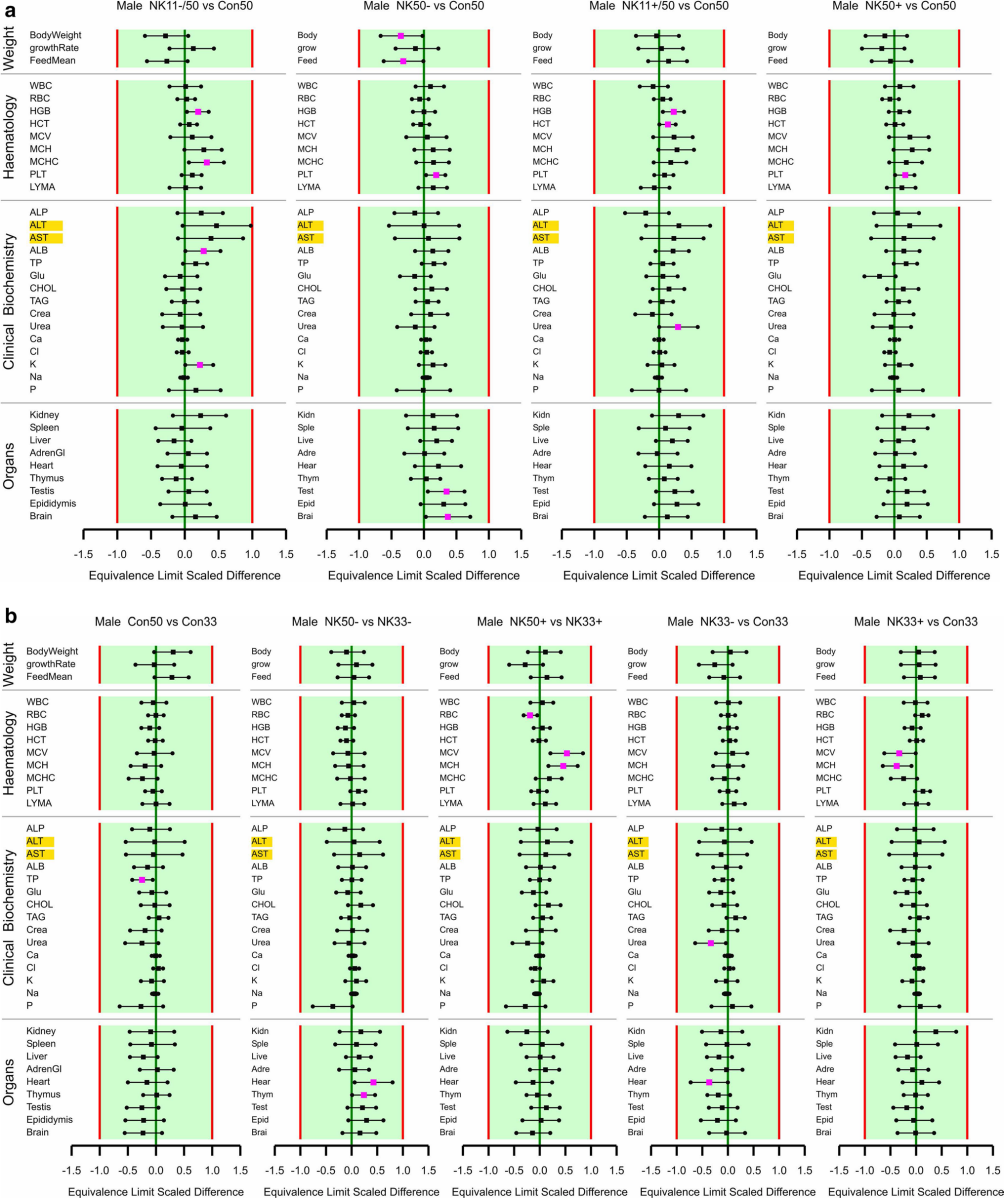

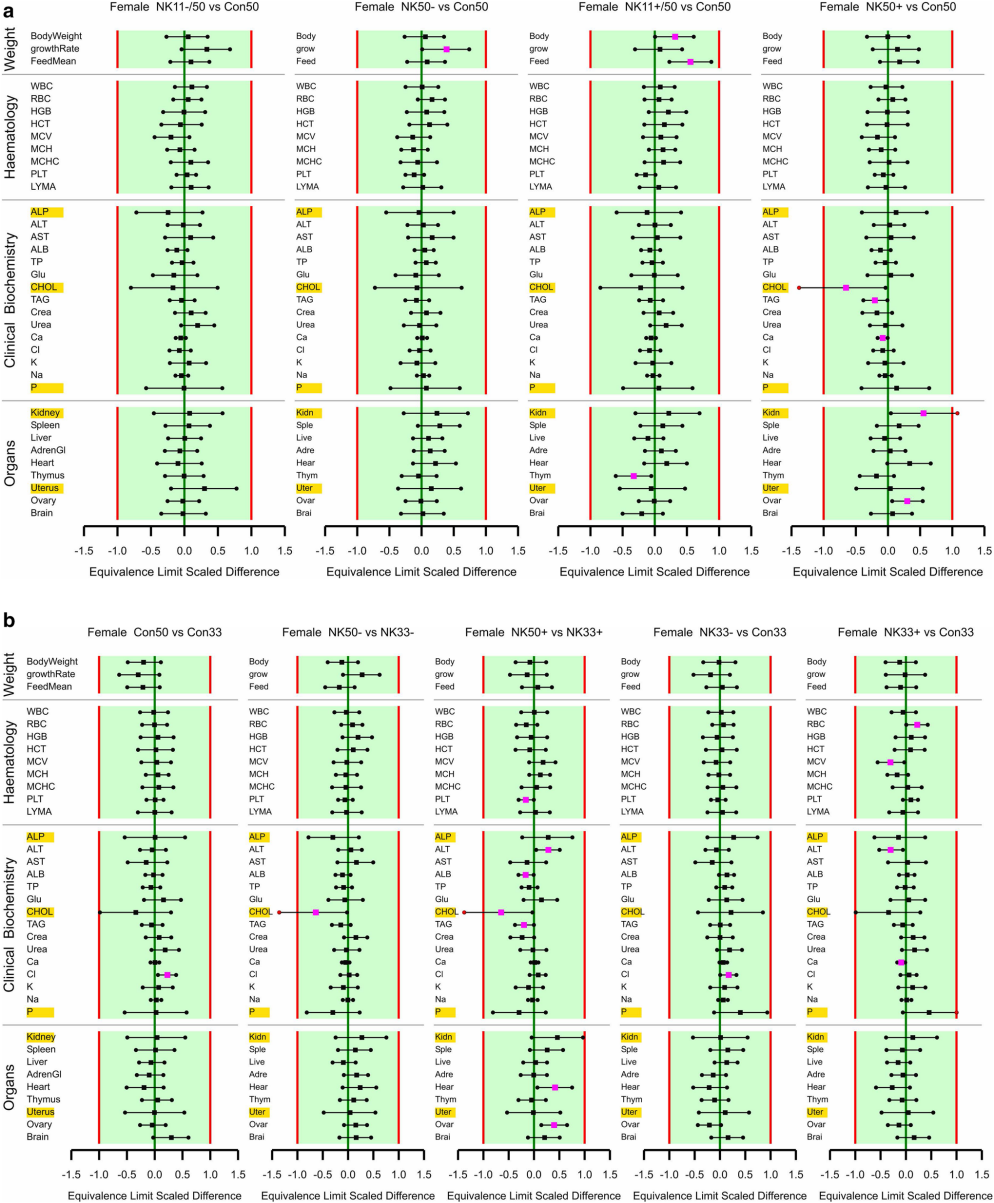
**a** Equivalence testing of 50% GM maize feeds versus the corresponding 50% non-GM control maize feed for male rats. **b** Equivalence testing of 50% maize feeds versus the corresponding 33% maize feeds and of 33% GM maize feeds versus the 33% non-GM maize feed for male rats. **c** Equivalence testing of 50% GM maize feeds versus the corresponding 50% non-GM control maize feed for female rats. **d** Equivalence testing of 50% maize feeds versus the corresponding 33% maize feeds and of 33% GM maize feeds versus the 33% non-GM maize feed for female rats. In all subplots, for estimates on the left of zero, the first mentioned feed has a smaller mean than the last mentioned feed. Endpoints labelled with a yellow background have a larger residual variance compared to the historical studies (variance ratio > 150%). Fuchsia coloured symbols denote a significant difference

The study design is shown in Table 7, while the body weight and the feed consumption of the rats are shown in Fig. 3a, b, respectively, with a summary on group comparisons in Fig. 4a–d. The body weight as well as the feed consumption during the 90-day feeding period was significantly decreased for male rats fed NK603 at the 50% inclusion rate relative to the 50% control diet. The body weight as well as the feed consumption of female rats during the 90-day feeding period was significantly increased for female rats fed NK603 + Roundup at an inclusion rate of 11% compared to the control group with an inclusion rate of 50%. The growth rate was significantly increased for female rats fed NK603 at an inclusion rate of 50%. Apart from these 5 significances, the other 49 comparisons involving the body weight, feed consumption and growth rate of male and female rats over 9 types of comparison (see headers in Fig. 4) showed no significant differences. The equivalence tests showed “equivalence” for all 54 comparisons, i.e. all ELSD intervals were fully inside the interval [− 1, + 1] (Fig. 4a–d).

**Table 7:** Study design of the 90-day feeding trial with the GM maize NK603 at an inclusion rate of up to 50% in the diet

Group	Content in the diet (%)			Number of animals	
	Near-isogenic non-GM	NK603	NK603 + Roundup	Males	Females
1	33	0	0	16	16
2	0	33	0	16	16
3	0	0	33	16	16
4	50	0	0	16	16
5	39	11	0	16	16
6	0	50	0	16	16
7	39	0	11	16	16
8	0	0	50	16	16
Sentinels				6	6
Total				134	134

The data on the haematological parameters measured in the blood samples of the male and female rats are shown in Table 8 and Fig. 4. In male rats, HGB in the group fed the 11% NK603 and the 11% NK603 + Roundup diets, PLT in the group fed the 50% NK603 and the 50% NK603 + Roundup diets and HCT in the group fed the 11% NK603 + Roundup diet were significantly higher than in the 50% control group, MCV and MCH were significantly lower in the group fed the 33% NK603 + Roundup than in the 33% control group and MCV and MCH were significantly higher in the group fed the 50% NK603 + Roundup diet than in the group fed the 33% NK603 + Roundup diet. Moreover, the percentage lymphocytes was significantly higher in male rats fed the 50% control diet than in the group fed the 33% control diet. The percentage neutrophils was significantly lower in male rats fed the 50% NK603 diet than in the groups fed the 50% control diet or the 33% NK603 diet. In female rats, RBC was significantly higher and MCV significantly lower in the group fed the 33% NK603 + Roundup diet than in the group fed the 33% control diet, while PLT was significantly lower in the group fed the 50% NK603 diet + Roundup than in the group fed the 33% NK603 + Roundup diet. Apart from these 14 significances, the other 238 comparisons involving the 14 haematological parameters in male and female rats over nine types of comparison (see headers in Fig. 4) showed no significant differences.

**Table 8:** Haematology parameters (mean ± SD) in the serum of male and female Wistar Han RCC rats in the 90-day feeding trial with GM maize NK603 at an inclusion rate of up to 50% in the diet

Parameter	33% Control	50% Control	11% NK603	33% NK603	50% NK603	11% NK603 + Roundup	33% NK603 + Roundup	50% NK603 + Roundup
Male rats								
WBC (10^9^/L)	8.36 ± 2.06	7.98 ± 1.53	8.01 ± 1.31	8.24 ± 1.15	8.56 ± 1.56	7.41 ± 1.01	8.22 ± 1.53	8.46 ± 1.45
RBC (10^12^/L)	8.63 ± 0.21	8.63 ± 0.14	8.67 ± 0.21	8.64 ± 0.18	8.54 ± 0.17	8.71 ± 0.21	8.79 ± 0.20	8.54 ± 0.20^c^
HGB (g/dL)	15.9 ± 0.40	15.7 ± 0.47	16.1 ± 0.41^a^	16.0 ± 0.28	15.7 ± 0.22	16.2 ± 0.36^a^	15.8 ± 0.32	15.9 ± 0.48
HCT (%)	46.9 ± 0.84	46.8 ± 1.27	47.3 ± 1.17	47.2 ± 0.84	46.5 ± 0.72	47.8 ± 0.88^a^	47.0 ± 1.00	46.9 ± 1.09
MCV (fL)	54.4 ± 0.85	54.3 ± 0.94	54.6 ± 0.79	54.6 ± 0.75	54.4 ± 0.54	54.9 ± 1.14	53.5 ± 0.68^b^	55.0 ± 0.94^c^
MCH (pg)	18.5 ± 0.53	18.2 ± 0.38	18.6 ± 0.33	18.5 ± 0.40	18.4 ± 0.26	18.6 ± 0.54	18.0 ± 0.43^b^	18.6 ± 0.26^c^
MCHC (g/dL)	34.0 ± 0.56	33.6 ± 0.46	34.1 ± 0.33	33.9 ± 0.45	33.8 ± 0.28	33.9 ± 0.47	33.6 ± 0.59	33.9 ± 0.39
PLT (10^9^/L)	770 ± 94	737 ± 66	792 ± 80	763 ± 78	859 ± 166^a^	776 ± 68	843 ± 83	829 ± 76^a^
LYMR (%)	75.2 ± 2.99	74.5 ± 3.13	74.5 ± 2.88	77.9 ± 3.13	76.3 ± 2.27	75.7 ± 3.00	73.0 ± 3.74	75.8 ± 2.62^c^
LYMA (10^3^/µL)	5.94 ± 1.27	5.92 ± 1.13	5.97 ± 1.08	6.41 ± 0.84	6.52 ± 1.08	5.61 ± 0.93	5.99 ± 1.06	6.40 ± 1.00
Lymphocytes (%)	67.0 ± 11.4	78.1 ± 8.9^b^	79.4 ± 10.7	74.1 ± 10.7	82.7 ± 12.6	74.4 ± 10.7	71.9 ± 12.9	73.8 ± 12.4
Neutrophils (%)	29.2 ± 10.4	18.8 ± 8.8	17.1 ± 9.6	21.3 ± 9.5	14.2 ± 12.3^a,e^	22.0 ± 9.5	24.4 ± 12.5	22.4 ± 11.1
Monocytes (%)	1.62 ± 1.22	1.97 ± 0.59	2.03 ± 1.80	2.78 ± 1.63	1.62 ± 1.10	2.09 ± 1.48	1.78 ± 0.83	2.00 ± 1.19
Eosinophils (%)	2.19 ± 2.06	1.16 ± 0.42	1.47 ± 0.75	1.84 ± 0.74	1.47 ± 0.83	1.50 ± 0.50	1.87 ± 0.69	1.84 ± 1.47
Female rats								
WBC (10^9^/L)	4.99 ± 0.91	5.01 ± 0.69	5.34 ± 0.80	5.17 ± 1.21	4.95 ± 1.00	5.25 ± 0.98	4.81 ± 1.02	4.91 ± 1.09
RBC (10^12^/L)	7.64 ± 0.20	7.64 ± 0.14	7.68 ± 0.17	7.70 ± 0.23	7.78 ± 0.31	7.69 ± 0.14	7.84 ± 0.10^b^	7.70 ± 0.17
HGB (g/dL)	15.0 ± 0.24	15.1 ± 0.42	15.1 ± 0.25	15.0 ± 0.74	15.2 ± 0.49	15.4 ± 0.28	15.2 ± 0.26	15.1 ± 0.24
HCT (%)	43.9 ± 0.90	44.0 ± 0.86	43.7 ± 1.08	44.0 ± 1.57	44.4 ± 1.65	44.5 ± 0.98	44.2 ± 0.73	43.9 ± 1.16
MCV (fL)	57.5 ± 1.09	57.6 ± 0.95	56.9 ± 0.68	57.2 ± 0.85	57.1 ± 0.56	57.9 ± 1.04	56.5 ± 0.90^b^	57.0 ± 0.92
MCH (pg)	19.7 ± 0.48	19.8 ± 0.46	19.7 ± 0.22	19.7 ± 0.50	19.6 ± 0.35	20.0 ± 0.38	19.4 ± 0.42	19.6 ± 0.45
MCHC (g/dL)	34.3 ± 0.40	34.4 ± 0.37	34.5 ± 0.35	34.4 ± 0.56	34.3 ± 0.52	34.6 ± 0.50	34.3 ± 0.58	34.4 ± 0.69
PLT (10^9^/L)	785 ± 46	787 ± 36	807 ± 57	773 ± 74.8	745 ± 93	734 ± 69	824 ± 46	760 ± 58^c^
LYMR (%)	77.9 ± 4.08	79.4 ± 3.72	78.1 ± 3.71	79.2 ± 3.44	79.8 ± 7.18	77.5 ± 2.48	78.0 ± 4.08	79.6 ± 4.97
LYMA (10^3^/µL)	3.91 ± 0.89	3.97 ± 0.59	4.16 ± 0.61	4.09 ± 0.98	3.96 ± 0.89	4.05 ± 0.66	3.79 ± 0.92	3.92 ± 1.09
Lymphocytes (%)	80.5 ± 8.3	72.7 ± 11.4	74.0 ± 7.4	73.8 ± 6.8	76.4 ± 7.0	72.4 ± 1.8	73.2 ± 7.5	74.8 ± 9.7
Neutrophils (%)	17.4 ± 7.6	24.8 ± 10.7	23.0 ± 7.1	23.1 ± 7.7	21.0 ± 7.1	25.3 ± 2.5	24.4 ± 7.4	21.9 ± 9.4
Monocytes (%)	0.88 ± 0.76	1.16 ± 1.00	1.41 ± 1.07	1.53 ± 0.90	1.31 ± 0.74	1.09 ± 0.93	0.97 ± 0.78	1.34 ± 0.79
Eosinophils (%)	1.16 ± 0.65	1.38 ± 0.78	1.53 ± 0.69	1.50 ± 1.07	1.25 ± 0.61	1.19 ± 0.88	1.44 ± 0.94	1.94 ± 0.85

Eight cages/group, two rats/cage

^a^Statistically significant difference to the 50% control value based on the 95% confidence interval

^b^Statistically significant difference to the 33% control value based on the 95% confidence interval

^c^Statistically significant difference to the 33% NK603 + Roundup value based on the 95% confidence interval

^d^Statistically significant difference to the 11% NK603 value based on the 95% confidence interval

^e^Statistically significant difference to the 33% NK603 value based on the 95% confidence interval

The differential white blood count data in the present study were much more variable than the historical control data. This indicates that the analytical methods used in the current and historical study were not comparable, and therefore, the historical data were not considered suitable to be used for equivalence tests (see details in Goedhart and van der Voet 2018i). The equivalence tests for all 162 comparisons involving the remaining 9 haematological parameters showed “equivalence”, i.e. all ELSD intervals were fully inside the interval [− 1, + 1] (Fig. 4a–d).

The data on the clinical biochemical parameters in male and female rats are shown in Table 9 and Fig. 4. In male rats, the TP level was lower in the group fed the 50% control diet than in the group fed the 33% control diet, whereas ALB and K levels were significantly higher in the group fed the 11% NK603 diet than in the group fed the 50% control diet. UREA was significantly lower in the group fed the 33% NK603 diet than in the 33% control group, while it was significantly higher in the 11% NK603 + Roundup group than in the 50% control group. In female rats, ALT was significantly higher in the group fed the 50% NK603 + Roundup diet than in the group fed the 33% NK603 + Roundup diet, whereas it was significantly lower in the group fed the 33% NK603 + Roundup diet than in the 33% control group. ALB was significantly lower in the group fed the 50% NK603 + Roundup diet than in the group fed the 33% NK603 + Roundup diet. CHOL was significantly lower in the 50% NK603 group than in the 33% NK603 group and the 33% NK603 + Roundup group; moreover, CHOL was significantly lower in the 50% NK603 + Roundup group than in the 50% control group. TAG levels were significantly lower in the group fed the 50% NK603 + Roundup diet than in the group fed the 50% control diet and the 33% NK603 + Roundup diet. CREA was significantly lower in the group fed the 50% NK603 + Roundup diet than in the group fed the 33% NK603 + Roundup diet. The Ca level was significantly lower in the group fed the 50% NK603 + Roundup diet than in the group fed the 50% control diet and in the group fed the 33% NK603 + Roundup diet when compared to the group fed the 33% control diet. Apart from these 16 significances, the other 254 comparisons involving the 15 clinical biochemical parameters in male and female rats over nine types of comparison (see headers in Fig. 4) showed no significant differences. The equivalence tests showed “equivalence” for 266 comparisons involving the 15 biochemical parameters, whereas the conclusion was “equivalence more likely than not” in four cases (Fig. 4a–d). One of the ELSD confidence limits was outside the interval [− 1, + 1] for CHOL in female rats fed the 50% NK603 + Roundup diet relative to the 50% control diet and the 33% NK603 + Roundup diet, for CHOL in female rats fed the 50% NK603 diet relative to the 33% NK603 diet and for P levels in female rats fed the 33% NK603 + Roundup diet relative to the 33% control diet (Fig. 4c, d).

**Table 9:** Clinical biochemistry parameters (mean ± SD) in the serum of male and female Wistar Han RCC rats in the 90-day feeding trial with GM maize NK603 at an inclusion rate of up to 50% in the diet

Parameter	33% Control	50% Control	11% NK603	33% NK603	50% NK603	11% NK603 + Roundup	33% NK603 + Roundup	50% NK603 + Roundup
Male rats								
ALP (µkat/L)	1.39 ± 0.29	1.28 ± 0.19	1.47 ± 0.32	1.29 ± 0.18	1.19 ± 0.15	1.20 ± 0.21	1.35 ± 0.22	1.33 ± 0.30
ALT (µkat/L)	0.53 ± 0.07	0.54 ± 0.10	0.66 ± 0.17	0.52 ± 0.08	0.53 ± 0.08	0.64 ± 0.34	0.55 ± 0.07	0.57 ± 0.10
AST (µkat/L)	2.42 ± 0.45	2.37 ± 0.37	2.92 ± 1.01	2.28 ± 0.30	2.46 ± 0.54	2.67 ± 0.68	2.39 ± 0.24	2.57 ± 0.71
ALB (g/L)	38.4 ± 1.58	37.5 ± 0.64	39.3 ± 2.95^a^	38.2 ± 1.48	38.3 ± 0.82	39.0 ± 3.75	38.3 ± 1.36	38.4 ± 0.67
TP (g/L)	66.2 ± 2.26	64.2 ± 0.76^b^	65.5 ± 2.10	65.4 ± 1.26	65.4 ± 0.67	64.6 ± 1.71	65.7 ± 1.69	65.6 ± 1.01
GLU (mmol/L)	5.74 ± 0.61	5.58 ± 0.90	5.45 ± 0.84	5.40 ± 0.33	5.22 ± 0.76	5.70 ± 0.48	5.29 ± 0.33	5.03 ± 0.69
CHOL (mmol/L)	2.15 ± 0.24	2.13 ± 0.26	2.11 ± 0.17	2.08 ± 0.17	2.23 ± 0.14	2.25 ± 0.09	2.11 ± 0.22	2.26 ± 0.26
TAG (mmol/L)	1.07 ± 0.24	1.13 ± 0.30	1.12 ± 0.17	1.27 ± 0.24	1.18 ± 0.17	1.18 ± 0.23	1.12 ± 0.22	1.20 ± 0.24
CREA (mmol/L)	36.2 ± 5.35	33.4 ± 5.31	32.3 ± 3.34	34.4 ± 4.11	34.9 ± 6.61	32.2 ± 7.05	33.3 ± 7.31	33.3 ± 5.95
UREA (mmol/L)	5.46 ± 0.97	4.96 ± 0.56	4.91 ± 0.90	4.84 ± 0.87^b^	4.69 ± 0.39	5.50 ± 0.79^a^	5.31 ± 0.57	4.86 ± 0.70
Ca (mmol/L)	2.39 ± 0.04	2.39 ± 0.04	2.37 ± 0.04	2.40 ± 0.05	2.40 ± 0.04	2.39 ± 0.04	2.39 ± 0.04	2.39 ± 0.03
Cl (mmol/L)	99 ± 1.95	100 ± 1.03	100 ± 1.90	100 ± 2.42	101 ± 2.19	100 ± 2.63	100 ± 2.31	99 ± 1.36
K (mmol/L)	4.56 ± 0.27	4.44 ± 0.21	4.89 ± 0.88^a^	4.51 ± 0.37	4.66 ± 0.37	4.50 ± 0.24	4.43 ± 0.19	4.56 ± 0.21
Na (mmol/L)	144 ± 2.43	144 ± 1.75	143 ± 1.67	143 ± 1.77	145 ± 1.70	143 ± 2.10	145 ± 2.40	143 ± 2.41
P (mmol/L)	2.19 ± 0.41	2.00 ± 0.14	2.11 ± 0.26	2.22 ± 0.19	1.99 ± 0.23	2.02 ± 0.42	2.22 ± 0.21	2.04 ± 0.19
Female rats								
ALP (µkat/L)	0.64 ± 0.08	0.66 ± 0.14	0.59 ± 0.11	0.72 ± 0.14	0.64 ± 0.13	0.63 ± 0.13	0.62 ± 0.16	0.68 ± 0.10
ALT (µkat/L)	0.50 ± 0.08	0.48 ± 0.06	0.48 ± 0.06	0.47 ± 0.06	0.49 ± 0.07	0.48 ± 0.04	0.43 ± 0.10^b^	0.50 ± 0.08^d^
AST (µkat/L)	2.40 ± 0.30	2.22 ± 0.45	2.36 ± 0.57	2.21 ± 0.38	2.44 ± 0.60	2.29 ± 0.58	2.48 ± 0.67	2.29 ± 0.43
ALB (g/L)	44.4 ± 2.39	44.0 ± 2.00	42.7 ± 1.48	46.1 ± 3.07	44.7 ± 1.87	43.1 ± 1.59	44.7 ± 2.08	42.7 ± 2.54^d^
TP (g/L)	70.4 ± 3.06	69.2 ± 2.44	68.6 ± 1.98	72.1 ± 4.36	70.4 ± 2.27	68.4 ± 2.05	70.1 ± 3.21	68.6 ± 4.49
GLU (mmol/L)	4.87 ± 0.56	5.24 ± 0.90	4.95 ± 0.76	5.16 ± 0.50	5.01 ± 0.77	5.25 ± 1.03	5.05 ± 0.72	5.36 ± 0.88
CHOL (mmol/L)	1.83 ± 0.34	1.63 ± 0.23	1.57 ± 0.41	1.95 ± 0.35	1.65 ± 0.44^c^	1.54 ± 0.30	1.68 ± 0.29	1.34 ± 0.19^a,d^
TAG (mmol/L)	0.56 ± 0.12	0.52 ± 0.09	0.51 ± 0.13	0.56 ± 0.07	0.48 ± 0.06	0.48 ± 0.09	0.54 ± 0.15	0.43 ± 0.11^a,d^
CREA (mmol/L)	35.4 ± 3.91	36.5 ± 3.11	38.5 ± 6.08	35.3 ± 3.06	37.7 ± 4.34	37.6 ± 3.50	37.6 ± 3.58	34.0 ± 4.00^d^
UREA (mmol/L)	5.48 ± 0.33	5.93 ± 0.59	6.47 ± 0.80	5.96 ± 0.76	5.87 ± 0.64	6.36 ± 0.54	5.90 ± 0.55	5.85 ± 0.81
Ca (mmol/L)	2.46 ± 0.05	2.46 ± 0.03	2.43 ± 0.05	2.49 ± 0.06	2.46 ± 0.04	2.43 ± 0.04	2.41 ± 0.03^b^	2.42 ± 0.03^a^
Cl (mmol/L)	101 ± 1.41	103 ± 1.87^b^	102 ± 1.83	102 ± 2.20	102 ± 0.99	102 ± 1.45	101 ± 1.33	102 ± 1.35
K (mmol/L)	4.09 ± 0.20	4.19 ± 0.23	4.31 ± 0.46	4.24 ± 0.48	4.09 ± 0.34	4.17 ± 0.46	4.32 ± 0.67	4.15 ± 0.46
Na (mmol/L)	143 ± 2.11	144 ± 1.28	143 ± 1.69	144 ± 1.49	144 ± 1.39	143 ± 0.90	144 ± 1.62	143 ± 1.51
P (mmol/L)	1.62 ± 0.36	1.65 ± 0.40	1.63 ± 0.32	1.84 ± 0.30	1.64 ± 0.17	1.66 ± 0.30	1.87 ± 0.38	1.71 ± 0.36

^a^Statistically significant difference to the 50% control value based on the 95% confidence interval

^b^Statistically significant difference to the 33% control value based on the 95% confidence interval

^c^Statistically significant difference to the 33% NK603 value based on the 95% confidence interval

^d^Statistically significant difference to the 33% NK603 + Roundup value based on the 95% confidence interval

The urinalysis data are summarized in the statistical report by Goedhart and van der Voet (2018f). The urine pH was significantly lower in male rats fed the 50% NK603 + Roundup diet than in the group fed the 33% NK603 + Roundup diet. In female rats, the urine volume and pH were significantly lower in the group fed the 50% NK603 diet than in the group fed the 33% NK603 diet, while the urine volume/body weight was significantly higher in the group fed the 33% NK603 diet than in the group fed the 33% control diet and the urine osmolarity was significantly lower in the group fed the 33% NK603 diet than in the group fed the 33% control diet. Apart from these 5 significances, the other 103 comparisons regarding the 6 urinalysis parameters showed no significant differences.

The relative organ weights in male and female rats are shown in Table 10 and Fig. 4. In male rats fed the 50% NK603 diet, the relative brain and testis weights were significantly higher than those in the 50% control group and the relative heart weight and thymus weight were significantly higher than that in the 33% NK603 group. The relative heart weight in the 33% NK603 group was significantly lower than in the 33% control group. In female rats fed the 50% NK603 + Roundup diet, the relative kidney and ovary weights were significantly higher than those in the 50% control group and the relative heart and ovary weights were significantly higher than those in the 33% NK603 + Roundup group. The relative thymus weight in the 11% NK603 + Roundup group was significantly lower than in the 50% control group. Apart from these 10 significances, the other 152 comparisons involving the 10 relative organ weights in male and female rats over 9 types of comparison (see headers in Fig. 4) showed no significant differences. The equivalence tests showed “equivalence” for all but one of the 162 comparisons, and “equivalence more likely than not” for relative kidney weight in female rats fed the 50% NK603 + Roundup diet relative to the 50% control diet (Fig. 4c).

**Table 10:** Relative organ weights in male and female Wistar Han RCC rats in the 90-day feeding trial with GM maize NK603 at an inclusion rate of up to 50% in the diet

Organ	Relative organ weights (organ weight/body weight × 100)							
	33% Control	50% Control	11% NK603	33% NK603	50% NK603	11% NK603 + Roundup	33% NK603 + Roundup	50% NK603 + Roundup
Male rats								
Adrenal glands	0.014 ± 0.001	0.014 ± 0.002	0.014 ± 0.002	0.013 ± 0.002	0.014 ± 0.002	0.014 ± 0.002	0.013 ± 0.002	0.014 ± 0.002
Brain	0.51 ± 0.02	0.49 ± 0.02	0.51 ± 0.03	0.51 ± 0.02	0.52 ± 0.03^a^	0.50 ± 0.02	0.51 ± 0.02	0.50 ± 0.04
Epididymides	0.29 ± 0.01	0.28 ± 0.03	0.28 ± 0.03	0.28 ± 0.03	0.30 ± 0.02	0.30 ± 0.01	0.29 ± 0.01	0.29 ± 0.02
Heart	0.26 ± 0.01	0.25 ± 0.01	0.25 ± 0.02	0.24 ± 0.01	0.26 ± 0.01^b^	0.26 ± 0.02	0.26 ± 0.02	0.26 ± 0.01
Kidneys	0.54 ± 0.03	0.54 ± 0.03	0.56 ± 0.03	0.53 ± 0.04	0.55 ± 0.04	0.56 ± 0.02	0.58 ± 0.04	0.55 ± 0.02
Liver	2.35 ± 0.10	2.27 ± 0.10	2.22 ± 0.10	2.29 ± 0.07	2.34 ± 0.10	2.34 ± 0.09	2.29 ± 0.08	2.30 ± 0.09
Spleen	0.17 ± 0.02	0.16 ± 0.01	0.16 ± 0.01	0.17 ± 0.02	0.17 ± 0.02	0.17 ± 0.02	0.17 ± 0.02	0.17 ± 0.02
Testes	0.86 ± 0.05	0.82 ± 0.06	0.83 ± 0.05	0.85 ± 0.08	0.88 ± 0.05^a^	0.86 ± 0.04	0.83 ± 0.07	0.86 ± 0.05
Thymus	0.10 ± 0.01	0.10 ± 0.01	0.09 ± 0.01	0.09 ± 0.01	0.10 ± 0.01	0.10 ± 0.02	0.10 ± 0.01	0.09 ± 0.01
Female rats								
Adrenal glands	0.031 ± 0.003	0.030 ± 0.001	0.029 ± 0.002	0.030 ± 0.004	0.032 ± 0.003	0.031 ± 0.002	0.030 ± 0.002	0.031 ± 0.003
Brain	0.81 ± 0.02	0.86 ± 0.05	0.85 ± 0.07	0.83 ± 0.04	0.86 ± 0.07	0.82 ± 0.06	0.83 ± 0.06	0.87 ± 0.08
Heart	0.31 ± 0.03	0.30 ± 0.01	0.30 ± 0.01	0.30 ± 0.02	0.31 ± 0.02	0.31 ± 0.02	0.30 ± 0.01	0.32 ± 0.03^b^
Kidneys	0.58 ± 0.02	0.58 ± 0.04	0.59 ± 0.05	0.58 ± 0.05	0.60 ± 0.03	0.60 ± 0.05	0.59 ± 0.04	0.63 ± 0.04^a^
Liver	2.44 ± 0.10	2.40 ± 0.07	2.41 ± 0.23	2.51 ± 0.19	2.46 ± 0.14	2.35 ± 0.13	2.35 ± 0.07	2.38 ± 0.11
Ovaries	0.040 ± 0.003	0.040 ± 0.005	0.039 ± 0.005	0.037 ± 0.006	0.040 ± 0.006	0.040 ± 0.003	0.039 ± 0.007	0.046 ± 0.009^a,b^
Spleen	0.21 ± 0.02	0.21 ± 0.01	0.22 ± 0.03	0.22 ± 0.02	0.23 ± 0.03	0.22 ± 0.02	0.21 ± 0.01	0.23 ± 0.03
Thymus	0.13 ± 0.02	0.13 ± 0.02	0.13 ± 0.02	0.13 ± 0.02	0.13 ± 0.02	0.12 ± 0.01	0.13 ± 0.01	0.12 ± 0.02
Uterus	0.25 ± 0.04	0.26 ± 0.07	0.30 ± 0.08	0.27 ± 0.07	0.27 ± 0.07	0.25 ± 0.06	0.26 ± 0.06	0.26 ± 0.06

^a^Statistically significant difference to the 50% control value based on the 95% confidence interval

^b^Statistically significant difference to the corresponding 33% (i.e. 33% NK603 or 33% NK603 + Roundup) value based on the 95% confidence interval

The summary tables with all necropsy findings observed in male and female rats are listed in the Supplementary Electronic Material’s Tables 8 and 9, respectively, those with all histopathological findings observed in male and female rats are listed in the Supplementary Electronic Material’s Tables 10 and 11, respectively. There were no treatment-related necropsy and histopathological findings following the feeding of NK603 or NK603 + Roundup to rats up to 50% inclusion rate for 90 days.

### Combined chronic toxicity/carcinogenicity feeding trial with the GM maize NK603 at an inclusion rate of 11 and 33% in the diets

The study design is shown in Table 11. The male and female rat mortality rates in the five experimental groups are shown in Table 12. The mortality rate of the male rats fed the 33% NK603 + Roundup diet was significantly higher than that of the corresponding control group (*p* = 0.03 in a one-sided test). In contrast, the female rats fed the 33% NK603 + Roundup diet showed a lower, though not significantly lower mortality rate than the corresponding control group (*p* = 0.07 if the test would have been performed for decreased rather than increased mortality).

**Table 11:** Study design of the combined chronic toxicity/carcinogenicity feeding trial with the GM maize NK603 at an inclusion rate of 11 and 33% in the diet

Group	Content in the diet (%)			No. of animals			
				Chronic toxicity		Carcinogenicity	
	Isogenic non-GM	NK603	NK603 + Roundup	Males	Females	Males	Females
1	33	0	0	20	20	50	50
2	22	11	0	20	20	50	50
3	0	33	0	20	20	50	50
4	22	0	11	20	20	50	50
5	0	0	33	20	20	50	50
Sentinels						10	10
Total				100	100	260	260

**Table 12:** Male and female rat mortality rate in the combined chronic toxicity/carcinogenicity feeding trial with the GM maize NK603 at an inclusion rate of 11 and 33% in the diet

	33% Control	11% NK603	33% NK603	11% NK603 + Roundup	33% NK603 + Roundup
Male rats	18/50 (36%)	14/50 (28%)	17/50 (34%)	18/50 (36%)	27/50 (54%)
Female rats	24/50 (48%)	26/50 (52%)	20/50 (40%)	19/50 (38%)	17/50 (34%)

Shown is the number of male and female rats that died or had to be sacrificed prematurely/total number of rats per group and the corresponding percentage in parentheses

**Fig. 5:**
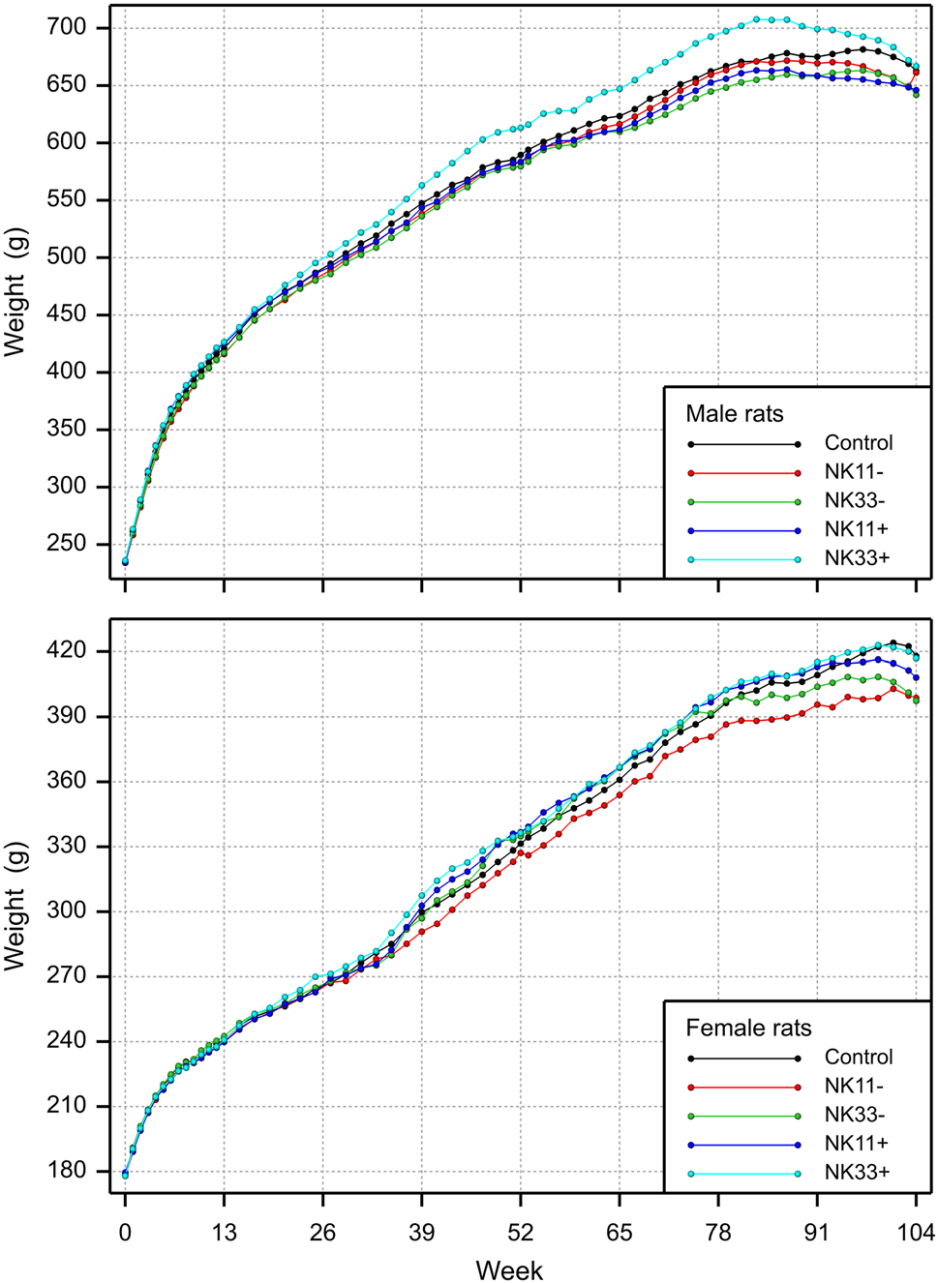
Mean body weight of all male and female rats surviving until the points in time plotted in the combined chronic toxicity/carcinogenicity feeding trial with GM maize NK603 at an inclusion rate of 11 and 33% in the diet

**Fig. 6:**
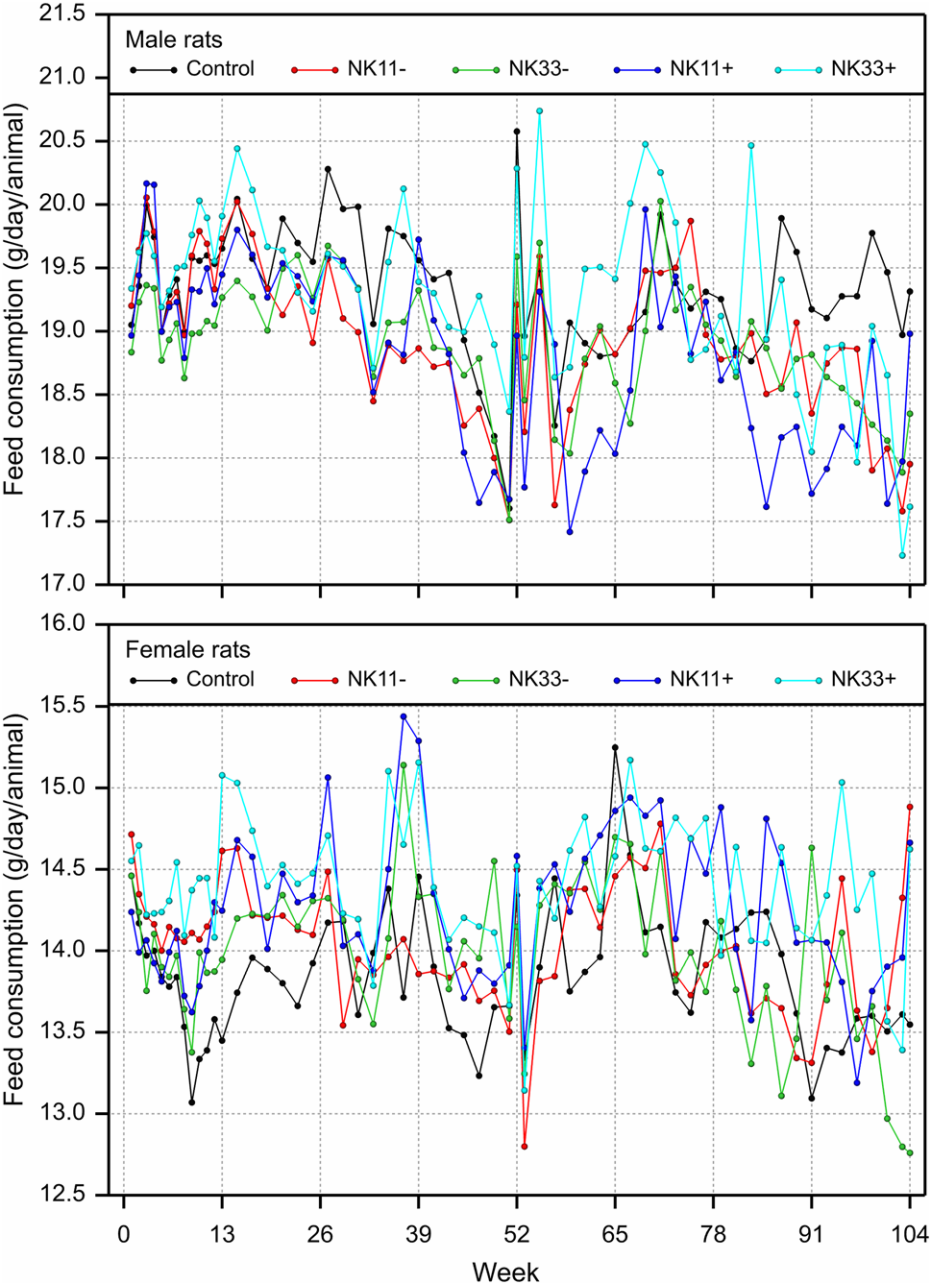
Mean feed consumption of all male and female rats surviving until the points in time plotted in the combined chronic toxicity/carcinogenicity feeding trial with GM maize NK603 at an inclusion rate of 11 and 33% in the diet

The body weight of the male and female rats surviving until the end of the 2-year feeding period is shown in Fig. 5. The male rats fed the NK603 + Roundup diet at an inclusion rate of 33% showed a significantly higher body weight when compared to the corresponding control group in the second half of the experimental period, while the body weight of the female rats was similar in all five experimental groups (Fig. 5). The mean feed consumption of male and female rats surviving until the end of the 2-year feeding period was similar to the respective control groups (Fig. 6). The equivalence tests were only performed for the data after 3 and 6 months, since there was only one set of control data from a 1-year feeding trial performed in the course of the preceding GRACE project, but no historical control data from a 2-year feeding trial at the animal housing facility. The equivalence tests showed “equivalence” for body weight and mean feed consumption at *t* = 3 and *t* = 6 months in male and female rats, i.e. all ELSD intervals were fully inside the interval [− 1, + 1]. Figures for all parameter groups are shown in Goedhart and van der Voet (2018a, i).

The data on the haematological parameters measured in the blood samples of the male rats are shown in Table 13. At *t* = 3 months, WBC were significantly higher in the group fed the 33% NK603 diet than in the control group. At *t* = 6 months, WBC and LYMA were significantly higher in the groups fed the 33% NK603 and 11% NK603 + Roundup diets than in the control group, while the percentage monocytes was significantly lower in the group fed the 11% NK603 diet than in the control group. At *t* = 12 months, HCT was significantly higher and MCHC was significantly lower in the group fed the 11% NK603 + Roundup diet than in the control group, while PLT was significantly higher in the groups fed the 33% NK603 and the 33% NK603 + Roundup diets than in the control group. The data on the haematological parameters measured in the blood samples of the female rats are shown in Table 14. At *t* = 6 months, PLT was significantly higher in the group fed the 11% NK603 + Roundup diet than in the control group, whereas the percentage monocytes was significantly lower in the group fed the 33% NK603 + Roundup diet than in the control group. At *t* = 12 months, MCH was significantly lower in the groups fed the 11% NK603 + Roundup and the 33% NK603 + Roundup diets and MCHC was significantly lower in the group fed the 11% NK603 + Roundup diet than in the control group. Moreover, the percentage lymphocytes was significantly lower and the percentage neutrophils significantly higher in the group fed the 11% NK603 diet than in the control group. At *t* = 24 months, the percentage eosinophils was significantly higher in the group fed the 33% NK603 diet than in the control group. Apart from these 21 cases of significance, the other 427 comparisons involving the 14 haematological parameters in male and female rats across 4 points in time and 4 NK603 groups showed no significant differences between the NK603 groups and the control group.

**Table 13:** Haematology parameters (mean ± SD) in the serum of male Wistar Han RCC rats in the combined chronic toxicity/carcinogenicity feeding trial with GM maize NK603 at an inclusion rate of 11 and 33% in the diet

Parameter	Time (months)	33% Control	11% NK603	33% NK603	11% NK603 + Roundup	33% NK603 + Roundup
Male rats						
WBC (10^9^/L)	3	10.1 ± 1.37	10.2 ± 2.08	11.5 ± 1.44^a^	10.9 ± 2.39	11.2 ± 2.60
	6	9.10 ± 1.63	8.90 ± 1.95	10.8 ± 1.88^a^	10.5 ± 1.84^a^	9.60 ± 1.94
	12	7.66 ± 1.59	7.20 ± 1.50	8.57 ± 1.59	7.81 ± 1.18	8.15 ± 1.08
	24	9.40 ± 3.57	9.90 ± 4.82	8.60 ± 2.97	9.80 ± 3.39	11.2 ± 5.70
RBC (10^12^/L)	3	8.64 ± 0.32	8.58 ± 0.28	8.61 ± 0.25	8.67 ± 0.27	8.60 ± 0.29
	6	8.46 ± 0.33	8.49 ± 0.31	8.47 ± 0.28	8.45 ± 0.28	8.52 ± 0.26
	12	8.48 ± 0.27	8.50 ± 0.35	8.45 ± 0.38	8.65 ± 0.35	8.58 ± 0.44
	24	7.89 ± 0.42	7.76 ± 0.63	7.96 ± 0.50	7.86 ± 0.69	7.88 ± 0.61
HGB (g/dL)	3	16.2 ± 0.50	16.1 ± 0.42	16.2 ± 0.40	16.0 ± 0.43	16.2 ± 0.45
	6	15.6 ± 0.51	15.7 ± 0.57	15.8 ± 0.53	15.6 ± 0.48	15.9 ± 0.43
	12	15.8 ± 0.26	15.9 ± 0.50	15.9 ± 0.49	16.0 ± 0.45	16.0 ± 0.61
	24	15.4 ± 0.90	15.0 ± 1.33	15.6 ± 0.90	15.3 ± 1.27	15.5 ± 1.16
HCT (%)	3	46.5 ± 1.66	46.1 ± 1.23	46.5 ± 1.36	46.7 ± 1.31	46.6 ± 1.52
	6	45.8 ± 1.54	46.0 ± 1.45	46.2 ± 1.67	45.6 ± 1.47	46.4 ± 1.25
	12	46.1 ± 1.29	46.3 ± 1.53	46.5 ± 2.07	47.2 ± 1.35^a^	46.8 ± 2.15
	24	44.1 ± 2.50	44.2 ± 2.70	44.5 ± 1.95	43.9 ± 3.27	44.2 ± 3.32
MCV (fL)	3	53.9 ± 1.44	53.8 ± 1.45	54.0 ± 1.20	53.8 ± 1.20	54.2 ± 1.19
	6	54.0 ± 0.78	54.3 ± 1.47	54.6 ± 1.48	54.0 ± 1.15	54.5 ± 0.98
	12	54.4 ± 1.55	54.6 ± 1.56	55.0 ± 1.56	54.6 ± 1.52	54.8 ± 1.20
	24	55.6 ± 2.10	56.5 ± 1.57	56.6 ± 2.09	56.0 ± 1.59	56.2 ± 1.86
MCH (pg)	3	18.7 ± 0.65	18.7 ± 0.69	18.9 ± 0.55	18.5 ± 0.49	18.8 ± 0.66
	6	18.5 ± 0.61	18.6 ± 0.82	18.7 ± 0.64	18.5 ± 0.55	18.7 ± 0.52
	12	18.7 ± 0.63	18.8 ± 0.73	18.8 ± 0.74	18.5 ± 0.76	18.7 ± 0.65
	24	19.5 ± 0.76	19.5 ± 0.71	19.6 ± 0.90	19.5 ± 0.86	19.7 ± 0.96
MCHC (g/dL)	3	34.8 ± 0.54	34.8 ± 0.56	34.9 ± 0.52	34.4 ± 0.50^a^	34.7 ± 0.76
	6	34.1 ± 0.62	34.2 ± 0.79	34.3 ± 0.61	34.3 ± 0.71	34.3 ± 0.56
	12	34.3 ± 0.57	34.3 ± 0.76	34.2 ± 0.90	33.9 ± 0.64^a^	34.2 ± 0.72
	24	34.9 ± 0.77	34.6 ± 0.67	34.7 ± 0.86	34.8 ± 0.76	35.1 ± 0.86
PLT (10^9^/L)	3	824 ± 123	847 ± 106	870 ± 124	860 ± 67	888 ± 107
	6	829 ± 44	812 ± 105	872 ± 88	864 ± 67	865 ± 112
	12	814 ± 66	807 ± 61	879 ± 84^a^	834 ± 81	883 ± 84^a^
	24	827 ± 118	850 ± 87	881 ± 187	867 ± 193	859 ± 146
LYMR (%)	3	73.3 ± 5.21	72.5 ± 5.90	72.2 ± 6.84	73.2 ± 4.60	73.7 ± 3.74
	6	71.4 ± 4.48	69.9 ± 4.90	70.5 ± 6.07	71.5 ± 6.06	72.3 ± 3.99
	12	66.9 ± 8.77	66.8 ± 5.02	69.5 ± 8.88	67.5 ± 8.09	66.5 ± 7.83
	24	58.4 ± 13.5	61.5 ± 14.1	61.0 ± 12.1	61.1 ± 11.8	56.9 ± 13.9
LYMA (10^3^/µL)	3	7.39 ± 1.11	7.52 ± 1.85	8.26 ± 1.34	7.98 ± 1.84	8.24 ± 2.14
	6	6.52 ± 1.17	6.19 ± 1.34	7.53 ± 1.33^a^	7.47 ± 1.37^a^	6.99 ± 1.67
	12	5.13 ± 1.25	4.98 ± 1.18	5.90 ± 1.23	5.25 ± 0.85	5.31 ± 1.10
	24	4.68 ± 1.84	5.14 ± 1.59	5.04 ± 2.03	5.31 ± 1.50	4.75 ± 1.54
Lymphocytes (%)	3	71.3 ± 4.1	70.8 ± 5.7	71.4 ± 5.2	70.7 ± 5.9	70.6 ± 8.5
	6	69.7 ± 5.2	70.8 ± 4.6	68.7 ± 5.3	70.0 ± 6.5	71.3 ± 4.3
	12	61.5 ± 6.0	60.5 ± 5.3	62.4 ± 6.0	60.2 ± 9.6	62.0 ± 5.5
	24	47.1 ± 14.7	45.1 ± 12.0	49.6 ± 7.8	48.8 ± 12.5	46.2 ± 15.0
Neutrophils (%)	3	24.7 ± 4.2	25.1 ± 5.7	25.9 ± 8.5	25.0 ± 6.0	25.4 ± 8.4
	6	27.0 ± 4.9	26.1 ± 4.0	27.4 ± 5.3	26.3 ± 5.8	25.1 ± 4.0
	12	36.4 ± 5.8	37.1 ± 5.5	35.0 ± 6.0	37.3 ± 9.0	35.7 ± 5.6
	24	48.2 ± 13.6	49.7 ± 11.5	45.6 ± 8.2	46.6 ± 12.6	50.0 ± 14.4
Monocytes (%)	3	1.9 ± 0.5	2.2 ± 0.8	2.0 ± 0.7	2.1 ± 0.8	2.0 ± 0.6
	6	1.2 ± 0.5	0.9 ± 0.4^a^	1.1 ± 0.5	1.0 ± 0.5	1.0 ± 0.4
	12	0.5 ± 0.3	0.6 ± 0.4	0.7 ± 0.4	0.7 ± 0.4	0.7 ± 0.5
	24	3.5 ± 2.8	3.7 ± 2.1	3.2 ± 1.7	3.2 ± 1.6	2.8 ± 2.3
Eosinophils (%)	3	2.1 ± 1.0	1.8 ± 1.1	2.2 ± 0.9	2.2 ± 1.0	2.0 ± 1.0
	6	2.2 ± 1.2	2.2 ± 1.3	2.8 ± 1.2	2.6 ± 1.7	2.5 ± 0.8
	12	1.6 ± 1.1	1.8 ± 1.1	1.9 ± 0.7	1.8 ± 1.4	1.6 ± 0.8
	24	1.2 ± 0.9	1.4 ± 1.0	1.6 ± 0.8	1.4 ± 1.2	1.0 ± 1.0

^a^Statistically significant difference to the 33% control value based on the 95% confidence interval

**Table 14:** Haematology parameters (mean ± SD) in the serum of female Wistar Han RCC rats in the combined chronic toxicity/carcinogenicity feeding trial with GM maize NK603 at an inclusion rate of 11 and 33% in the diet

Parameter	Time (months)	33% Control	11% NK603	33% NK603	11% NK603 + Roundup	33% NK603 + Roundup
Female rats						
WBC (10^9^/L)	3	7.54 ± 1.63	7.84 ± 1.24	7.80 ± 2.24	7.28 ± 1.12	8.02 ± 1.36
	6	5.86 ± 1.35	5.97 ± 0.94	6.12 ± 1.71	5.59 ± 1.27	6.05 ± 1.30
	12	5.57 ± 1.34	5.94 ± 1.53	5.13 ± 0.96	5.45 ± 1.19	5.88 ± 1.28
	24	7.80 ± 0.59	7.80 ± 2.60	6.90 ± 3.40	10.4 ± 10.2	8.10 ± 3.60
RBC (10^12^/L)	3	7.62 ± 0.33	7.62 ± 0.31	7.73 ± 0.35	7.70 ± 0.29	7.73 ± 0.37
	6	7.66 ± 0.30	7.64 ± 0.25	7.71 ± 0.30	7.68 ± 0.19	7.72 ± 0.27
	12	7.37 ± 0.36	7.37 ± 0.31	7.44 ± 0.35	7.55 ± 0.29	7.54 ± 0.25
	24	7.19 ± 0.57	7.15 ± 0.45	7.19 ± 0.68	7.08 ± 0.77	7.26 ± 0.50
HGB (g/dL)	3	15.5 ± 0.59	15.4 ± 0.52	15.6 ± 0.57	15.4 ± 0.52	15.4 ± 0.51
	6	15.6 ± 0.44	15.4 ± 0.52	15.5 ± 0.29	15.4 ± 0.29	15.5 ± 0.41
	12	15.4 ± 0.53	15.4 ± 0.52	15.5 ± 0.51	15.6 ± 0.48	15.5 ± 0.54
	24	15.2 ± 0.56	14.9 ± 0.84	14.8 ± 0.87	15.0 ± 1.09	15.1 ± 0.67
HCT (%)	3	44.4 ± 1.31	44.2 ± 1.35	44.9 ± 1.03	44.1 ± 0.88	44.8 ± 1.74
	6	44.1 ± 1.55	44.0 ± 1.45	44.3 ± 1.03	44.1 ± 0.88	44.2 ± 1.41
	12	43.2 ± 2.00	43.3 ± 1.90	43.8 ± 1.63	44.1 ± 1.56	43.9 ± 1.38
	24	42.6 ± 2.62	42.0 ± 2.82	41.3 ± 2.84	42.2 ± 2.81	42.1 ± 1.75
MCV (fL)	3	58.4 ± 2.27	58.4 ± 1.50	58.0 ± 1.90	58.0 ± 1.52	58.0 ± 1.73
	6	57.7 ± 0.96	57.6 ± 0.90	57.4 ± 1.14	57.4 ± 0.80	57.2 ± 0.99
	12	58.7 ± 0.86	58.9 ± 1.16	58.7 ± 1.25	58.4 ± 0.98	58.2 ± 1.19
	24	58.2 ± 1.91	58.8 ± 2.09	57.6 ± 2.30	58.5 ± 1.77	57.7 ± 1.66
MCH (pg)	3	20.4 ± 1.15	20.4 ± 1.10	20.2 ± 0.89	20.0 ± 0.86	20.0 ± 0.76
	6	20.4 ± 0.52	20.2 ± 0.53	20.1 ± 0.69	20.1 ± 0.49	20.1 ± 0.64
	12	21.0 ± 0.55	21.0 ± 0.60	20.8 ± 0.75	20.6 ± 0.42^a^	20.6 ± 0.50^a^
	24	20.9 ± 0.81	20.9 ± 0.87	20.8 ± 1.24	20.8 ± 1.19	20.8 ± 0.99
MCHC (g/dL)	3	34.9 ± 0.94	34.9 ± 0.94	34.8 ± 0.99	34.6 ± 1.14	34.5 ± 0.95
	6	35.3 ± 0.76	35.1 ± 0.76	34.9 ± 0.40	34.9 ± 0.59	35.2 ± 0.87
	12	35.8 ± 0.72	35.6 ± 0.86	35.3 ± 0.73	35.2 ± 0.63^a^	35.4 ± 0.82
	24	35.8 ± 0.59	35.6 ± 0.90	35.5 ± 1.02	35.5 ± 0.93	35.8 ± 0.72
PLT (10^9^/L)	3	828 ± 71	799 ± 81	787 ± 87	813 ± 84	846 ± 79
	6	843 ± 82	794 ± 63	812 ± 69	786 ± 92^a^	819 ± 117
	12	755 ± 75	792 ± 103	751 ± 74	764 ± 79	784 ± 97
	24	784 ± 102	763 ± 165	837 ± 168	842 ± 199	889 ± 190
LYMR (%)	3	73.7 ± 5.05	71.3 ± 7.26	72.7 ± 6.30	72.5 ± 8.03	74.1 ± 6.25
	6	72.4 ± 3.94	71.2 ± 4.03	71.6 ± 5.22	72.2 ± 3.88	71.1 ± 5.25
	12	69.9 ± 7.41	70.0 ± 5.44	71.4 ± 7.50	70.0 ± 6.10	70.6 ± 3.68
	24	56.8 ± 9.10	55.2 ± 10.3	56.5 ± 9.40	57.5 ± 10.6	56.8 ± 9.80
LYMA (10^3^/µL)	3	5.56 ± 1.22	5.69 ± 1.15	5.57 ± 1.41	5.22 ± 0.83	6.02 ± 1.38
	6	4.21 ± 0.85	4.26 ± 0.75	4.35 ± 1.21	4.03 ± 0.94	4.28 ± 1.04
	12	3.86 ± 0.97	3.92 ± 0.63	3.57 ± 0.60	3.78 ± 0.67	4.04 ± 0.80
	24	3.47 ± 1.29	4.11 ± 1.58	3.23 ± 0.89	3.44 ± 0.86	3.72 ± 1.36
Lymphocytes (%)	3	72.9 ± 7.5	71.9 ± 7.2	73.8 ± 6.7	71.2 ± 6.5	73.2 ± 7.0
	6	67.7 ± 4.3	67.5 ± 4.3	67.4 ± 3.9	67.5 ± 3.5	69.3 ± 4.5
	12	68.4 ± 7.4	62.8 ± 6.4^a^	66.5 ± 9.0	66.2 ± 6.7	66.7 ± 6.6
	24	49.6 ± 14.3	46.7 ± 11.6	52.9 ± 8.1	47.3 ± 13.5	50.0 ± 13.2
Neutrophils (%)	3	23.2 ± 6.4	24.4 ± 7.1	23.2 ± 6.6	24.9 ± 6.0	22.6 ± 6.4
	6	29.0 ± 4.3	29.5 ± 4.1	29.6 ± 4.1	29.3 ± 3.8	27.9 ± 4.3
	12	29.0 ± 7.3	34.3 ± 6.5^a^	30.7 ± 9.1	31.3 ± 6.9	30.1 ± 6.1
	24	46.8 ± 13.9	49.8 ± 10.9	42.9 ± 8.5	48.8 ± 11.7	46.2 ± 13.4
Monocytes (%)	3	1.9 ± 0.7	1.7 ± 0.7	1.7 ± 0.6	2.0 ± 0.9	2.0 ± 0.7
	6	1.1 ± 0.6	0.9 ± 0.6	1.0 ± 0.6	0.9 ± 0.4	0.7 ± 0.4^a^
	12	0.7 ± 0.9	0.8 ± 0.6	0.9 ± 0.7	0.8 ± 0.9	1.0 ± 0.9
	24	2.3 ± 2.2	2.2 ± 1.4	2.4 ± 1.4	2.7 ± 2.5	2.4 ± 1.6
Eosinophils (%)	3	2.1 ± 1.2	2.0 ± 1.2	1.3 ± 0.8	1.9 ± 0.8	2.2 ± 1.1
	6	2.2 ± 0.8	2.2 ± 0.9	2.0 ± 1.0	2.3 ± 0.8	2.1 ± 1.2
	12	1.9 ± 0.7	2.2 ± 1.2	1.9 ± 1.0	1.6 ± 0.6	2.2 ± 0.7
	24	1.2 ± 0.7	1.4 ± 0.9	1.8 ± 1.1^a^	1.2 ± 0.7	1.4 ± 0.8

^a^Statistically significant difference to the 33% control value based on the 95% confidence interval

The differential white blood count data in the present data were much more variable than the historical reference data. This indicates that the analytical methods used in the current and historical study were not comparable, and therefore, the historical data were not considered suitable to be used for equivalence tests (see details in Goedhart and van der Voet 2018i). The equivalence tests for the remaining 9 haematological parameters showed “equivalence” in the case of 131 comparisons, “equivalence more likely than not” for 12 comparisons, and “non-equivalence more likely than not” for one of the 144 comparisons with the control group. The latter case, in which the ELSD point estimate was outside the equivalence area between − 1 and + 1, was the percentage monocytes in female rats fed the 33% NK603 + Roundup diet at *t* = 6 months. Furthermore, one or both ELSD confidence limits were outside the interval [− 1, + 1] for MCV in male rats fed any NK603 diet at *t* = 3 months and male rats fed the 33% NK603 or the 33% NK603 + Roundup diet at *t* = 6 months, for MCH in male rats fed any NK603 diet at *t* = 6 months, as well as for MCHC in female rats fed the 33% NK603 + Roundup diet at *t* = 3 months or the 11% NK603 + Roundup diet or the 33% NK603 diet at *t* = 6 months.

The data on the clinical biochemical parameters in male rats are shown in Table 15. The TAG levels were significantly higher in the groups fed the 11% NK603 + Roundup diet than in the control group at *t* = 6 and 12 months, while UREA was significantly higher in the group fed the 11% NK603 diet than in the control group at *t* = 24 months. Cl was significantly lower in the groups fed the 33% NK603, 11% NK603 + Roundup and 33% NK603 + Roundup diets than in the control group at *t* = 12 months, whereas K was significantly higher in the group fed the 11% NK603 + Roundup diet than in the control group at *t* = 24 months and P was significantly lower in the group fed the 11% NK603 diet than in the control group at *t* = 12 months. The data on the clinical biochemical parameters in female rats are shown in Table 16. ALT was significantly lower in the group fed the 11% NK603 + Roundup diet than in the control group after 12 months and AST was significantly higher in the group fed the 33% NK603 + Roundup diet than in the control group at *t* = 6 months. TAG was significantly lower in the group fed the 11% NK603 + Roundup diet than in the control group at *t* = 6 months. CREA was significantly higher in the group fed the 11% NK603 diet than in the control group at *t* = 12 months and was significantly higher in the groups fed the 33% NK603, 11% NK603 + Roundup and 33% NK603 + Roundup diets than in the control group at *t* = 24 months. UREA was significantly higher in all four groups fed the NK603 containing diets than in the control group at *t* = 12 months. Cl was significantly higher in the group fed the 11% NK603 + Roundup diet than in the control group at *t* = 3 months, while it was significantly lower in the group fed the 11% NK603 diet than in the control group at *t* = 24 months. K was significantly higher in the group fed the 33% NK603 diet than in the control group at *t* = 3 months and in the group fed the 11% NK603 diet than in the control group at *t* = 24 months. Na was significantly higher in the group fed the 11% NK603 + Roundup diet than in the control group at *t* = 3 months and in the groups fed the 11% NK603 and 33% NK603 diets than in the control group at *t* = 12 months. P was significantly higher in the group fed the 33% NK603 diet than in the control group at *t* = 3 months. Apart from these 30 cases of significance, the other 450 comparisons involving the 15 clinical biochemical parameters for males and females over 4 time points and 4 NK603 groups showed no significant differences between the NK603 fed groups and the group fed the control diet. Likewise, the equivalence tests at *t* = 3 and *t* = 6 months showed “equivalence” for 225 comparisons involving the 15 clinical biochemical parameters, and “equivalence more likely than not” for 15 comparisons: one or both ELSD confidence limits were outside the interval [− 1, + 1] for CHOL in female rats fed the 33% NK603, the 11% NK603 + Roundup or the 33% NK603 + Roundup diet at *t* = 3 months, or fed any NK603 diet at *t* = 6 months, and P levels in female rats fed the 33% NK603 diet at *t* = 3 months.

**Table 15:** Clinical biochemistry parameters (mean ± SD) in the serum of male Wistar Han RCC rats in the combined chronic toxicity/carcinogenicity feeding trial with GM maize NK603 at an inclusion rate of 11 and 33% in the diet

Parameter	Time (months)	33% Control	11% NK603	33% NK603	11% NK603 + Roundup	33% NK603 + Roundup
ALP (µkat/L)	3	1.51 ± 0.33	1.54 ± 0.37	1.47 ± 0.26	1.47 ± 0.26	1.56 ± 0.41
	6	1.39 ± 0.30	1.44 ± 0.36	1.34 ± 0.30	1.34 ± 0.30	1.37 ± 0.34
	12	1.23 ± 0.22	1.33 ± 0.26	1.26 ± 0.21	1.26 ± 0.21	1.26 ± 0.22
	24	1.09 ± 0.44	1.18 ± 0.56	0.95 ± 0.35	0.95 ± 0.35	0.84 ± 0.23
ALT (µkat/L)	3	0.66 ± 0.10	0.68 ± 0.16	0.64 ± 0.11	0.64 ± 0.10	0.64 ± 0.10
	6	0.68 ± 0.09	0.70 ± 0.16	0.70 ± 0.15	0.68 ± 0.12	0.69 ± 0.10
	12	0.66 ± 0.11	0.68 ± 0.19	0.64 ± 0.11	0.72 ± 0.18	0.69 ± 0.14
	24	0.83 ± 0.17	0.90 ± 0.30	0.88 ± 0.26	0.88 ± 0.24	0.90 ± 0.27
AST (µkat/L)	3	2.27 ± 0.47	2.25 ± 0.34	2.18 ± 0.30	2.08 ± 0.48	2.12 ± 0.28
	6	2.09 ± 0.41	2.15 ± 0.42	2.25 ± 0.42	2.15 ± 0.32	2.11 ± 0.31
	12	2.30 ± 0.38	2.39 ± 0.52	2.29 ± 0.33	2.28 ± 0.54	2.32 ± 0.58
	24	2.60 ± 0.66	3.05 ± 0.94	2.73 ± 0.66	2.85 ± 0.95	2.80 ± 0.82
ALB (g/L)	3	39.7 ± 1.92	39.2 ± 2.27	39.4 ± 1.86	38.8 ± 1.44	39.9 ± 2.78
	6	35.0 ± 1.24	35.0 ± 1.32	35.3 ± 1.35	35.2 ± 1.32	35.2 ± 1.35
	12	37.7 ± 1.22	37.4 ± 1.05	38.0 ± 1.02	37.5 ± 1.10	37.5 ± 1.37
	24	35.8 ± 3.43	36.1 ± 4.04	37.7 ± 2.15	37.2 ± 4.13	36.0 ± 3.11
TP (g/L)	3	67.7 ± 2.12	67.6 ± 2.57	67.1 ± 2.06	67.4 ± 1.86	68.0 ± 3.04
	6	64.6 ± 1.99	64.3 ± 1.59	64.4 ± 1.72	65.0 ± 1.53	64.4 ± 1.78
	12	69.0 ± 1.80	68.4 ± 1.79	69.3 ± 1.51	68.8 ± 2.05	68.8 ± 1.80
	24	71.3 ± 3.71	71.8 ± 4.79	72.4 ± 3.11	71.7 ± 4.61	71.7 ± 3.74
GLU (mmol/L)	3	5.99 ± 0.83	5.86 ± 0.73	5.86 ± 0.91	6.12 ± 0.79	6.24 ± 0.91
	6	5.94 ± 0.81	5.79 ± 0.78	5.87 ± 0.75	6.04 ± 0.78	6.19 ± 0.57
	12	5.35 ± 0.59	5.43 ± 0.82	5.26 ± 0.47	5.69 ± 1.16	5.40 ± 0.60
	24	4.18 ± 1.10	4.26 ± 1.08	4.36 ± 1.15	4.08 ± 1.12	3.94 ± 1.26
CHOL (mmol/L)	3	2.13 ± 0.21	2.17 ± 0.22	2.14 ± 0.25	2.08 ± 0.22	2.17 ± 0.24
	6	2.20 ± 0.27	2.26 ± 0.23	2.23 ± 0.28	2.21 ± 0.27	2.29 ± 0.30
	12	2.74 ± 0.35	2.82 ± 0.38	2.85 ± 0.50	2.64 ± 0.45	2.88 ± 0.65
	24	4.59 ± 1.60	4.31 ± 2.01	4.03 ± 1.14	4.31 ± 1.98	4.72 ± 1.87
TAG (mmol/L)	3	1.01 ± 0.23	1.14 ± 0.34	1.14 ± 0.33	1.16 ± 0.37	1.03 ± 0.25
	6	1.24 ± 0.50	1.29 ± 0.48	1.33 ± 0.47	1.39 ± 0.53^a^	1.30 ± 0.43
	12	1.78 ± 0.59	1.82 ± 0.45	2.01 ± 0.58	2.05 ± 0.67^a^	2.01 ± 0.56
	24	2.08 ± 0.95	2.62 ± 1.26	2.25 ± 0.88	2.40 ± 1.52	2.52 ± 1.47
CREA (mmol/L)	3	37.4 ± 4.34	35.4 ± 4.64	35.9 ± 5.59	36.2 ± 4.29	36.0 ± 4.79
	6	42.3 ± 3.67	41.0 ± 3.78	41.8 ± 3.95	42.4 ± 3.52	40.8 ± 3.73
	12	43.7 ± 4.80	42.1 ± 3.30	43.1 ± 2.49	42.1 ± 3.35	42.3 ± 3.66
	24	30.4 ± 6.90	35.2 ± 22.6	31.0 ± 12.9	30.7 ± 9.20	41.9 ± 33.4
UREA (mmol/L)	3	5.85 ± 0.65	5.61 ± 1.06	5.70 ± 0.85	5.69 ± 0.68	5.78 ± 0.58
	6	5.16 ± 0.61	5.02 ± 0.75	5.16 ± 0.59	5.19 ± 0.60	5.10 ± 0.51
	12	4.37 ± 0.39	4.32 ± 0.49	4.38 ± 0.46	4.58 ± 0.58	4.28 ± 0.54
	24	4.03 ± 0.72	5.00 ± 1.92^a^	4.32 ± 1.12	4.42 ± 0.91	4.35 ± 1.18
Ca (mmol/L)	3	2.54 ± 0.10	2.55 ± 0.09	2.55 ± 0.09	2.54 ± 0.08	2.54 ± 0.10
	6	2.52 ± 0.04	2.52 ± 0.05	2.54 ± 0.06	2.54 ± 0.06	2.52 ± 0.05
	12	2.54 ± 0.03	2.55 ± 0.05	2.55 ± 0.05	2.54 ± 0.06	2.54 ± 0.04
	24	2.53 ± 0.09	2.54 ± 0.12	2.51 ± 0.08	2.49 ± 0.06	2.54 ± 0.11
Cl (mmol/L)	3	104 ± 2.72	104 ± 1.63	104 ± 1.98	103 ± 1.46	105 ± 2.09
	6	102 ± 1.36	102 ± 1.38	101 ± 1.44	101 ± 1.25	102 ± 1.18
	12	104 ± 1.31	103 ± 1.54	102 ± 1.33^a^	103 ± 1.80^a^	103 ± 1.65^a^
	24	102 ± 1.83	102 ± 1.64	102 ± 1.58	103 ± 2.13	102 ± 2.48
K (mmol/L)	3	5.34 ± 0.62	5.42 ± 0.55	5.39 ± 0.74	5.35 ± 0.53	5.47 ± 0.86
	6	5.06 ± 0.32	5.17 ± 0.59	5.25 ± 0.46	5.32 ± 0.48	5.13 ± 0.37
	12	4.80 ± 0.34	4.77 ± 0.23	4.88 ± 0.30	4.81 ± 0.26	4.80 ± 0.27
	24	4.61 ± 0.40	4.72 ± 0.49	4.60 ± 0.27	4.85 ± 0.43^a^	4.69 ± 0.41
Na(mmol/L)	3	146 ± 3.28	145 ± 2.20	146 ± 1.90	146 ± 2.15	146 ± 2.33
	6	144 ± 1.80	144 ± 2.23	144 ± 1.78	144 ± 1.69	142 ± 1.18
	12	143 ± 2.07	144 ± 1.84	143 ± 1.55	144 ± 2.28	143 ± 2.74
	24	143 ± 1.48	143 ± 2.24	144 ± 1.49	144 ± 2.19	143 ± 2.27
P (mmol/L)	3	2.57 ± 0.22	2.43 ± 0.25^a^	2.56 ± 0.34	2.48 ± 0.19	2.47 ± 0.27
	6	2.04 ± 0.26	1.99 ± 0.27	2.05 ± 0.20	2.09 ± 0.21	2.02 ± 0.18
	12	1.77 ± 0.17	1.67 ± 0.19^a^	1.81 ± 0.28	1.71 ± 0.17	1.69 ± 0.16
	24	1.67 ± 0.36	1.81 ± 0.41	1.52 ± 0.26	1.64 ± 0.30	1.83 ± 0.62

^a^Statistically significant difference to the 33% control value based on the 95% confidence interval

**Table 16:** Clinical biochemistry parameters (mean ± SD) in the serum of female Wistar Han RCC rats in the combined chronic toxicity/carcinogenicity feeding trial with GM maize NK603 at an inclusion rate of 11 and 33% in the diet

Parameter	Time (months)	33% Control	11% NK603	33% NK603	11% NK603 + Roundup	33% NK603 + Roundup
ALP (µkat/L)	3	0.68 ± 0.14	0.69 ± 0.13	0.71 ± 0.18	0.73 ± 0.14	0.71 ± 0.21
	6	0.65 ± 0.13	0.61 ± 0.14	0.63 ± 0.16	0.62 ± 0.13	0.61 ± 0.16
	12	0.59 ± 0.13	0.55 ± 0.08	0.60 ± 0.16	0.59 ± 0.13	0.54 ± 0.12
	24	0.58 ± 0.18	0.56 ± 0.17	0.60 ± 0.18	0.59 ± 0.12	0.55 ± 0.12
ALT (µkat/L)	3	0.52 ± 0.12	0.53 ± 0.16	0.51 ± 0.10	0.48 ± 0.08	0.57 ± 0.14
	6	0.62 ± 0.14	0.61 ± 0.13	0.64 ± 0.12	0.55 ± 0.10^a^	0.61 ± 0.09
	12	0.74 ± 0.18	0.70 ± 0.18	0.71 ± 0.15	0.65 ± 0.27	0.72 ± 0.13
	24	0.85 ± 0.23	0.80 ± 0.17	0.81 ± 0.15	0.83 ± 0.15	0.83 ± 0.16
AST (µkat/L)	3	2.14 ± 0.37	2.11 ± 0.40	2.28 ± 0.30	2.09 ± 0.36	2.43 ± 0.48^a^
	6	1.95 ± 0.36	1.84 ± 0.34	2.11 ± 0.56	1.81 ± 0.30	2.09 ± 0.40
	12	2.59 ± 0.62	2.52 ± 0.49	2.69 ± 0.50	2.55 ± 0.81	2.73 ± 0.73
	24	3.15 ± 0.98	2.92 ± 2.26	3.27 ± 0.68	3.21 ± 0.86	3.10 ± 0.68
ALB (g/L)	3	45.8 ± 2.87	45.8 ± 4.52	45.7 ± 2.96	46.0 ± 3.10	45.2 ± 4.50
	6	41.5 ± 1.93	41.8 ± 2.27	42.0 ± 2.93	42.4 ± 2.56	41.3 ± 2.70
	12	42.4 ± 1.91	42.5 ± 2.12	43.0 ± 2.14	43.3 ± 3.00	42.4 ± 2.16
	24	40.0 ± 2.84	39.5 ± 2.26	39.6 ± 2.49	38.8 ± 2.44	40.1 ± 2.42
TP (g/L)	3	71.3 ± 3.34	70.7 ± 4.41	70.8 ± 3.13	71.5 ± 3.64	70.9 ± 4.01
	6	69.6 ± 2.59	69.7 ± 2.73	70.2 ± 3.54	70.2 ± 2.75	69.1 ± 2.37
	12	71.9 ± 2.11	71.8 ± 2.30	72.1 ± 2.23	71.9 ± 2.63	71.7 ± 2.72
	24	73.8 ± 4.58	74.8 ± 3.98	75.6 ± 3.53	73.0 ± 3.76	74.9 ± 3.46
GLU (mmol/L)	3	5.15 ± 0.53	5.20 ± 0.81	4.93 ± 0.74	5.20 ± 0.77	4.89 ± 0.57
	6	5.55 ± 0.67	5.37 ± 0.76	5.26 ± 0.61	5.45 ± 0.74	5.48 ± 0.74
	12	5.03 ± 0.87	5.04 ± 0.60	4.75 ± 0.71	5.10 ± 0.70	5.18 ± 0.84
	24	4.96 ± 0.86	5.19 ± 1.20	4.75 ± 1.01	4.81 ± 1.16	4.58 ± 0.87
CHOL (mmol/L)	3	1.98 ± 0.34	1.90 ± 0.33	1.83 ± 0.34	1.78 ± 0.22	1.85 ± 0.33
	6	2.23 ± 0.42	2.16 ± 0.41	2.06 ± 0.42	2.01 ± 0.32	2.10 ± 0.35
	12	2.64 ± 0.50	2.61 ± 0.50	2.57 ± 0.47	2.47 ± 0.45	2.49 ± 0.36
	24	3.19 ± 0.76	3.29 ± 1.74	2.97 ± 0.87	2.76 ± 0.80	3.31 ± 1.14
TAG (mmol/L)	3	0.70 ± 0.13	0.68 ± 0.16	0.63 ± 0.11	0.61 ± 0.13^a^	0.65 ± 0.08
	6	0.82 ± 0.24	0.85 ± 0.40	0.74 ± 0.17	0.74 ± 0.20	0.78 ± 0.19
	12	0.96 ± 0.22	0.90 ± 0.23	1.01 ± 0.31	1.21 ± 1.08	0.98 ± 0.25
	24	1.32 ± 0.47	1.48 ± 0.65	1.18 ± 0.47	1.39 ± 0.69	1.60 ± 1.58
CREA (mmol/L)	3	44.5 ± 4.04	46.2 ± 5.08	45.2 ± 3.43	47.2 ± 5.23	46.0 ± 4.87
	6	43.3 ± 7.27	42.5 ± 4.87	41.5 ± 3.88	43.7 ± 6.78	43.5 ± 3.48
	12	43.2 ± 4.47	45.4 ± 3.66	45.2 ± 3.86	46.1 ± 4.99^a^	44.3 ± 3.46
	24	32.4 ± 4.13	35.2 ± 3.65	37.5 ± 6.27^a^	35.7 ± 5.26^a^	36.6 ± 5.37^a^
UREA (mmol/L)	3	5.76 ± 0.54	5.89 ± 0.83	5.83 ± 0.51	5.88 ± 0.50	6.13 ± 0.81
	6	5.52 ± 0.55	5.76 ± 0.54	5.64 ± 0.55	5.50 ± 0.42	5.68 ± 0.49
	12	4.63 ± 0.44	4.99 ± 0.48^a^	5.17 ± 0.57^a^	5.05 ± 0.68^a^	4.98 ± 0.54^a^
	24	4.67 ± 0.61	4.82 ± 0.83	4.98 ± 0.67	4.93 ± 0.78	4.67 ± 0.75
Ca (mmol/L)	3	2.51 ± 0.06	2.52 ± 0.08	2.53 ± 0.07	2.52 ± 0.08	2.52 ± 0.06
	6	2.53 ± 0.05	2.54 ± 0.07	2.56 ± 0.07	2.55 ± 0.07	2.53 ± 0.05
	12	2.57 ± 0.04	2.58 ± 0.05	2.58 ± 0.05	2.59 ± 0.06	2.56 ± 0.03
	24	2.51 ± 0.06	2.54 ± 0.09	2.51 ± 0.08	2.51 ± 0.06	2.51 ± 0.09
Cl (mmol/L)	3	100 ± 2.44	101 ± 2.70	101 ± 1.99	101 ± 3.49^a^	101 ± 2.94
	6	101 ± 1.58	102 ± 1.45	102 ± 1.67	102 ± 1.48	101 ± 1.85
	12	101 ± 1.52	101 ± 1.24	100 ± 1.43	100 ± 1.50	101 ± 1.66
	24	97 ± 2.31	96 ± 2.51^a^	97 ± 2.94	97 ± 2.64	97 ± 2.52
K (mmol/L)	3	4.46 ± 0.41	4.52 ± 0.27	4.54 ± 0.32	4.46 ± 0.39	4.57 ± 0.39
	6	4.17 ± 0.32	4.22 ± 0.29	4.56 ± 0.57^a^	4.32 ± 0.31	4.19 ± 0.35
	12	4.36 ± 0.28	4.38 ± 0.40	4.36 ± 0.29	4.37 ± 0.39	4.42 ± 0.52
	24	4.34 ± 0.34	3.99 ± 0.39^a^	4.31 ± 0.49	4.43 ± 0.50	4.32 ± 0.45
Na(mmol/L)	3	142 ± 2.14	143 ± 2.52	143 ± 2.49	143 ± 2.98^a^	143 ± 2.77
	6	142 ± 2.28	142 ± 2.04	142 ± 1.97	142 ± 2.06	143 ± 2.19
	12	141 ± 1.92	142 ± 2.28^a^	142 ± 1.80^a^	142 ± 2.17	142 ± 2.30
	24	142 ± 2.03	142 ± 2.73	143 ± 2.05	142 ± 2.16	141 ± 1.85
P (mmol/L)	3	1.78 ± 0.33	1.87 ± 0.25	2.00 ± 0.33^a^	1.77 ± 0.43	1.96 ± 0.64
	6	1.34 ± 0.21	1.33 ± 0.23	1.41 ± 0.26	1.35 ± 0.26	1.29 ± 0.16
	12	1.45 ± 0.23	1.46 ± 0.22	1.56 ± 0.24	1.57 ± 0.37	1.42 ± 0.25
	24	1.53 ± 0.22	1.50 ± 0.29	1.71 ± 0.41	1.62 ± 0.22	1.48 ± 0.25

^a^Statistically significant difference to the 33% control value based on the 95% confidence interval

The urinalysis data are summarized in the statistical reports by Goedhart and van der Voet (2018a, b, c, d, e). In male rats, at *t* = 3 months, the urine volume was significantly lower and the urine leukocyte number as well as the urine ketone level were significantly higher in the group fed the 33% NK603 diet than in the control group. At *t* = 6 months, the urine volume was significantly lower in the group fed the 33% NK603 diet than in the control group. At *t* = 12 months, the urine volume and the urine volume/body weight were significantly lower and the urine osmolarity and the urine ketone level were significantly higher in the group fed the 33% NK603 diet than in the control group, while the urine volume, urine volume/body weight and urine leukocyte number were significantly lower in the group fed the 11% NK603 diet than in the control group. At *t* = 24 months, the urine ketone level was significantly higher in the group fed the 33% NK603 + Roundup diet than in the control group. In female rats, at *t* = 3 months, the urine ketone level was higher in the group fed the 11% NK603 + Roundup diet than in the control group, while the urine volume/bodyweight was lower in the group fed the 33% NK603 + Roundup diet than in the control group. At *t* = 24 months, the urine pH was higher in the group fed the 33% NK603 diet than in the control group. Apart from these 13 cases of significance, the other 243 comparisons involving 8 urinalysis parameters in male and female rats over 4 points in time and 4 NK603 groups showed no significant differences between the NK603-fed groups and the group fed the control diet.

The relative organ weights in male and female rats at *t* = 12 months are shown in Table 17. In male rats fed the 11% NK603 diet, the relative epididymides weight was significantly higher than that in the control group. In female rats, the relative brain weight was significantly higher in the four groups fed the NK603 diets, whereas the relative kidney weight was significantly higher in the groups fed the 11% NK603 + Roundup and the 33% NK603 + Roundup diets than in the control group. Apart from these 7 cases of significance, the other 57 comparisons involving the 9 relative organ weights over 4 time points and 4 NK603 groups for males and females were not statistically significant between the NK603 fed groups and the group fed the control diet.

**Table 17:** Relative organ weights in male and female Wistar Han RCC rats in the combined chronic toxicity/carcinogenicity feeding trial with GM maize NK603 at an inclusion rate of 11 and 33% in the diet (at *t* = 12 months)

Organ	Relative organ weights (organ weight/body weight × 100)				
	33% Control	11% NK603	33% NK603	11% NK603 + Roundup	33% NK603 + Roundup
Male rats					
Adrenal glands	0.009 ± 0.001	0.009 ± 0.001	0.009 ± 0.001	0.008 ± 0.001	0.009 ± 0.001
Brain	0.38 ± 0.03	0.40 ± 0.05	0.39 ± 0.04	0.37 ± 0.04	0.39 ± 0.04
Epididymides	0.22 ± 0.02	0.24 ± 0.03^a^	0.22 ± 0.03	0.21 ± 0.03	0.23 ± 0.03
Heart	0.20 ± 0.01	0.21 ± 0.02	0.22 ± 0.02	0.20 ± 0.01	0.20 ± 0.01
Kidneys	0.45 ± 0.04	0.47 ± 0.04	0.48 ± 0.08	0.45 ± 0.05	0.45 ± 0.04
Liver	2.10 ± 0.19	2.08 ± 0.21	2.11 ± 0.14	2.09 ± 0.12	2.01 ± 0.20
Spleen	0.17 ± 0.02	0.17 ± 0.01	0.18 ± 0.02	0.17 ± 0.03	0.17 ± 0.02
Testes	0.64 ± 0.05	0.69 ± 0.08	0.65 ± 0.08	0.65 ± 0.06	0.68 ± 0.08
Female rats					
Adrenal glands	0.019 ± 0.003	0.019 ± 0.002	0.019 ± 0.003	0.020 ± 0.003	0.020 ± 0.003
Brain	0.59 ± 0.06	0.65 ± 0.07^a^	0.64 ± 0.07^a^	0.64 ± 0.06^a^	0.65 ± 0.04^a^
Heart	0.25 ± 0.02	0.26 ± 0.02	0.27 ± 0.03	0.26 ± 0.02	0.26 ± 0.02
Kidneys	0.48 ± 0.05	0.51 ± 0.04	0.50 ± 0.05	0.52 ± 0.05^a^	0.52 ± 0.04^a^
Liver	2.01 ± 0.19	2.05 ± 0.18	2.02 ± 0.15	2.06 ± 0.11	2.06 ± 0.15
Ovaries	0.018 ± 0.006	0.019 ± 0.003	0.021 ± 0.004	0.021 ± 0.007	0.021 ± 0.006
Spleen	0.20 ± 0.03	0.21 ± 0.02	0.19 ± 0.03	0.20 ± 0.03	0.22 ± 0.05
Uterus	0.26 ± 0.06	0.28 ± 0.07	0.24 ± 0.06	0.25 ± 0.05	0.25 ± 0.05

*Statistically significant difference to the control value based on the 95% confidence interval

The complete histopathology report is available via the internet portal CADIMA. As previously mentioned, the mortality rate of the male rats fed the 33% NK603 + Roundup diet was significantly higher than that of the corresponding control group. Tables 18 and 19 list the causes of premature death in male rats fed the control diet and the 33% NK603 + Roundup diet, respectively. The most common cause of premature death in both groups was a pituitary pars anterior adenoma, 12 in the control group and 17 in the group fed the 33% NK603 diet. The next most common cause of premature death was a kidney chronic progressive nephropathy, 1 in the control group and 3 in the group fed the 33% NK603 + Roundup diet.

**Table 18:** Causes of premature death in male rats fed the control diet in the course of the combined chronic toxicity/carcinogenicity feeding trial

Animal no.	Death week	Death codes	Primary tumor site/main organ affected	Death context	Cause of death or intercurrent sacrifice
303	75	+ 1	Kidneys		Chronic progressive nephropathy
304	87	+ 2	Pituitary gland	B4	Adenoma; pars anterior
306	99	+ 1	Pituitary gland	B4	Adenoma; pars anterior
309	84	+ 2	Pituitary gland	B4	Adenoma; pars anterior
310	87	+ 1	Pituitary gland	B4	Adenoma; pars anterior
316	86	+ 2	Pituitary gland	B4	Adenoma; pars anterior
318	98	+ 1	–	–	Urogenital inflammation
319	71	+ 2	Pituitary gland	B4	Adenoma; pars anterior
321	79	+ 2	Abdominal cavity	N4	Liposarcoma
323	89	+ 1	–	–	Urogenital inflammation
324	74	+ 2	Skin/subcutis	N4	Fibrosarcoma
326	96	+ 2	Pituitary gland	B4	Adenoma; pars anterior
332	71	+ 1	Pituitary gland	B4	Adenoma; pars anterior
337	87	+ 1	Pituitary gland	B4	Adenoma; pars anterior
340	75	+ 1	Pituitary gland	B4	Adenoma; pars anterior
343	92	+ 2	Pituitary gland	B4	Adenoma; pars anterior
344	75	+ 1	Pituitary gland	B4	Adenoma; pars anterior
347	20	+ 2	Head	N4	Squamous carcinoma

Death codes: + 1 = found dead, + 2 = sacrificed moribund; death context: B4 = benign neoplasm, definitely fatal, N4 = malignant neoplasm, definitely fatal

**Table 19:** Causes of premature death in male rats fed the 33% NK603 + Roundup diet in the course of the combined chronic toxicity/carcinogenicity feeding trial

Animal no.	Death week	Death codes	Primary tumor site/main organ affected	Death context	Cause of death or intercurrent sacrifice
2	50	+ 2	Pituitary gland	B0	Adenoma; pars anterior
21	102	+ 2	Skin/subcutis		Abscess
23	77	+ 2	Pituitary gland	B4	Adenoma; pars anterior
26	98	+ 1	Kidneys		Chronic progressive nephropathy
27	97	+ 2	Glandular stomach		Ulceration
29	100	+ 1	Pituitary gland	B4	Adenoma; pars anterior
30	103	+ 2	Pituitary gland	B4	Adenoma; pars anterior
32	77	+ 2	Pituitary gland	B4	Adenoma; pars anterior
33	51	+ 2	Pituitary gland	B4	Adenoma; pars anterior
34	96	+ 2	Pituitary gland	B4	Adenoma; pars anterior
35	54	+ 1	–	–	Pancreatitis
37	77	+ 2	Brain	N4	Meningioma; malignant
39	78	+ 2	Systemic neoplasms	N4	Histiocytic sarcoma
40	86	+ 2	Pituitary gland	B4	Adenoma; pars anterior
43	68	+ 2	Pituitary gland	B4	Adenoma; pars anterior
44	89	+ 2	Adrenal medullas	N4^a^	Pheochromocytoma; malignant
46	83	+ 2	Kidneys		Chronic progressive nephropathy
47	91	+ 2	Pituitary gland	B4	Adenoma; pars anterior
49	86	+ 2	Pituitary gland	B4	Adenoma; pars anterior
53	92	+ 1	Head	N4	Squamous carcinoma
55	89	+ 2	Pituitary gland	B3	Adenoma; pars anterior
56	99	+ 2	Kidneys		Chronic progressive nephropathy
57	98	+ 2	Pituitary gland	B4	Adenoma; pars anterior
58	86	+ 2	Pituitary gland	B4	Adenoma; pars anterior
60	84	+ 1	Adrenal medullas	N4^a^	Pheochromocytoma; malignant
66	82	+ 2	Pituitary gland	B4	Adenoma; pars anterior
68	93	+ 1	Pituitary gland	B4	Adenoma; pars anterior
70	99	+ 2	Pituitary gland	B4	Adenoma; pars anterior
49	86	+ 2	Pituitary gland	B4	Adenoma; pars anterior
53	92	+ 1	Head	N4	Squamous carcinoma

Death codes: +1 = found dead, + 2 = sacrificed moribund; death context: ^a^finding unilateral in paired organs, B0 = benign neoplasm; B3 = benign neoplasm, probably fatal, B4 = benign neoplasm, definitely fatal, N4 = malignant neoplasm, definitely fatal

The necropsy findings in the chronic toxicity phase recorded in this study were considered to be within the normal range of background alterations seen in untreated animals of this age and strain (McInnes 2012; Blankenship and Skaggs 2013; Cesta et al. 2014). The macroscopic findings in the brain, pituitary gland and mammary glands are listed in Table 20. The deformation of the brain correlated with the microscopic findings of compression by a pituitary neoplasm and mammary nodules and masses correlated with the microscopic findings of mammary neoplasia (see below). All microscopic findings observed in the rats in the chronic toxicity phase are listed in the Supplementary Electronic Material’s Table 12. The microscopic findings in the pituitary gland and mammary glands are listed in Table 21. There were no statistically significant differences in the number of macroscopic and microscopic findings between control rats and the NK603-fed rats.

**Table 20:** Macroscopic findings in the brain, pituitary gland and mammary glands in the chronic toxicity phase of the combined chronic toxicity/carcinogenicity feeding trial with the GM maize NK603

	Number of animals with macroscopic findings									
	Male rats					Female rats				
	33% Control	11% NK603	33% NK603	11% NK603 + Roundup	33% NK603 + Roundup	33% Control	11% NK603	33% NK603	11% NK603 + Roundup	33% NK603 + Roundup
Number of animals	20	20	20	20	20	20	20	20	20	20
Brain										
Deformation	1	–	–	–	1	–	1	1	–	–
Pituitary gland										
Enlarged	–	–	1	–	1	–	–	–	–	1
Nodule	–	–	–	–	–	–	1	1	–	–
Mammary gland										
Nodule	–	–	–	–	–	2	1	1	–	–

**Table 21:** Microscopic findings in the pituitary gland and mammary glands in the chronic toxicity phase of the combined chronic toxicity/carcinogenicity feeding trial with the GM maize NK603

	Number of animals with microscopic findings									
	Male rats					Female rats				
	33% Control	11% NK603	33% NK603	11% NK603 + Roundup	33% NK603 + Roundup	33% Control	11% NK603	33% NK603	11% NK603 + Roundup	33% NK603 + Roundup
Number of animals	20	20	20	20	20	20	20	20	20	20
Pituitary gland										
Adenoma pars anterior	2	3	1	2	3	3	–	–	3	1
Mammary gland										
Adenocarcinoma	–	–	–	–	–	–	1	–	–	–
Fibroadenoma	–	–	–	–	–	2	–	–	–	–

The necropsy findings in the carcinogenicity phase recorded in this study were considered to be within the normal range of background alterations seen in untreated animals of this age and strain (McInnes 2012; Cesta et al. 2014). The macroscopic findings in the brain, pituitary gland, mammary glands and thymus are listed in Table 22. The deformation of the brain correlated with microscopic findings of compression by a pituitary neoplasm (see below). The findings in the thymus (enlarged and nodule) correlated with microscopic findings of thymoma (see below). The mammary nodules and masses correlated with microscopic findings of mammary neoplasia (see below).

**Table 22:** Macroscopic findings in the brain, pituitary gland, mammary glands and thymus in the carcinogenicity phase of the combined chronic toxicity/carcinogenicity feeding trial with the GM maize NK603

	Number of animals with macroscopic findings									
	Male rats					Female rats				
	33% Control	11% NK603	33% NK603	11% NK603 + Roundup	33% NK603 + Roundup	33% Control	11% NK603	33% NK603	11% NK603 + Roundup	33% NK603 + Roundup
Number of animals	50	50	50	50	50	50	50	50	50	50
Brain										
Deformation	12	7	10	7-	16	14	18	22	14	14
Pituitary gland										
Enlarged	21	10	16	13	26	20	26	24	27	25
Nodule	2	1	1	1	2	1	–	2	–	1
Mammary gland										
Nodule	–	–	–	–	–	1	6	8	3	2
Mass	–	–	1	–	2	20	22	15	14	15
Thymus										
Enlarged	3	3	1	4	1	4	6	4	3	6
Nodule	1	5	1	2	1	1	2	2	2	1

All non-neoplastic microscopic findings observed in the rats in the carcinogenicity phase are listed in the Supplementary Electronic Material’s Table 13. There were no treatment-related differences in the number of non-neoplastic microscopic findings between control rats and NK603-fed rats. The neoplastic lesions observed in male and female rats in the chronic toxicity phase of the combined chronic toxicity/carcinogenicity feeding trial with NK603 are shown in Table 23. All neoplastic microscopic findings observed in the rats fed the 33% control, 33% NK603 and 33% NK603 + Roundup diets in the carcinogenicity phase are listed in Table 24. There were no treatment-related differences in the number of neoplastic microscopic findings between control rats and NK603-fed rats. The observed increase in the number of benign thymomas in the female group fed NK603 + Roundup at an inclusion rate of 33% was not statistically significant when compared to the control group, or when benign and malignant thymomas were analyzed in combination. Moreover, this was also the case of pituitary gland and mammary gland tumours.

**Table 23:** Neoplastic lesions in male and female rats fed the 33% control, the 33% NK603 and the 33% NK603 + Roundup diets in the chronic toxicity phase of the combined chronic toxicity/carcinogenicity feeding trial with the GM maize NK603

Organ/lesion	33% Control	33% NK603	33% NK603 + Roundup
Males			
Thyroid glands	20^a^	18	20
C-cell adenoma	–	–	1^b^
Pituitary gland	20	20	20
Adenoma; pars anterior	2	3	3
Females			
Lung	20	20	20
Metastasis	–	–	1
Thyroid glands	19	20	20
C-cell adenoma	–	–	1
Follicular cell adenoma	–	1	–
Mammary gland area	20	19	20
Fibroadenoma	2	–	–
Uterus	20	20	20
Endometrial stromal polyp	–	1	–
Pituitary gland	20	20	20
Adenoma; pars anterior	3	3	1
Abdominal cavity	7	5	6
Metastasis	–	–	1
Bone	–	–	1
Osteosarcoma	–	–	1

^a^Number of animals analyzed

^b^Number of animals with a particular tumor

**Table 24:** Neoplastic lesions in male and female rats fed the 33% control, the 33% NK603 and the 33% NK603 + Roundup diets in the carcinogenicity phase of the combined chronic toxicity/carcinogenicity feeding trial with the GM maize NK603

Organ/lesion	Males			Females		
	33% Control	33% NK603	33% NK603 + Roundup	33% Control	33% NK603	33% NK603 + Roundup
Systemic neoplasms	–	–	1^a^	–	1	–
Histiocytic sarcoma	–	–	1^b^	–	–	–
Malignant lymphoma	–	–	–	–	1	–
Heart	50	50	50	50	50	50
Schwannoma; benign	–	–	1	–	–	–
Metastasis	–	–	–	1	–	–
Liver	50	50	50	50	50	50
Adenoma; hepatocyte	1	2	4	2	1	3
Cholangiocarcinoma	–	1	–	–	–	–
Metastasis	1	–	–	–	–	–
Spleen	50	50	50	50	50	50
Hemangioma	–	1	–	–	–	–
Hemangiosarcoma	–	1	1	–	–	–
Metastasis	–	1	–	–	–	–
Mesenteric lymph node	50	50	49	50	50	49
Hemangioma	–	–	2	2	1	–
Metastasis	–	–	–	–	–	1
Kidneys	50	50	50	50	50	49
Local invasion	–	–	–	–	–	1
Tubular cell adenoma	–	2	–	–	–	1
Urinary bladder	50	50	50	50	50	49
Papilloma	–	–	–	–	1	–
Jejunum	50	49	50	50	50	50
Leiomyoma	–	–	–	1	1	1
Forestomach	50	50	50	50	50	50
Papilloma; squamous	–	–	2	–	–	–
Lung	50	50	50	50	50	50
Metastasis	–	–	–	3	2	1
Thymus	47	48	44	46	45	47
Thymoma; benign	4	3	–	6	7	11
Thymoma; malignant	–	–	1	–	1	–
Testes	50	50	50	–	–	–
Leydig cell adenoma	1	2	1	–	–	–
Epididymides	50	50	50	–	–	–
Mesothelioma	–	1	–	–	–	–
Ovaries	–	–	–	50	50	50
Granulosa adenoma	–	–	–	–	1	–
Granulosa carcinoma	–	–	–	–	–	1
Sex cord-stromal tumor; benign	–	–	–	8	3	8
Mandibular lymph nodes	49	50	50	49	49	48
Metastasis	1	–	–	–	–	1
Pancreas	49	50	49	50	50	50
Acinar cell adenoma	1	–	1	–	–	–
Islet cell adenoma	6	8	2	–	–	2
Islet cell carcinoma	2	1	1	1	1	–
Metastasis	1	1	–	–	–	–
Thyroid glands	50	50	49	49	50	49
C-cell adenoma	5	6	5	6	7	2
C-cell carcinoma	–	–	–	3	1	–
Follicular cell adenoma	5	2	1	2	2	–
Follicular cell carcinoma	2	–	–	–	–	–
Adrenal cortices	50	50	50	50	50	50
Adenoma	4	–	2	3	2	2
Carcinoma	–	–	–	–	1	–
Local invasion	–	–	–	–	–	1
Adrenal medullas	50	50	50	48	50	50
Ganglioneuroma	1	–	–	–	–	–
Neuroblastoma	–	–	–	–	–	1
Pheochromocytoma; benign	3	1	1	3	1	3
Pheochromocytoma; malignant	1	–	3	–	–	–
Skeletal muscle	50	50	50	50	50	50
Fibroma	–	–	1	–	–	–
Fibrosarcoma	–	–	–	–	1	–
Rhabdomyosarcoma	1	–	–	–	–	–
Skin/subcutis	49	50	50	50	50	50
Basal cell adenoma	–	1	–	–	–	1
Fibroma	1	1	2	1	–	1
Fibrosarcoma	2	–	–	–	–	–
Keratoacanthoma	–	–	2	–	–	–
Lipoma	1	1	1	–	–	–
Myxoma	–	1	–	–	–	–
Schwannoma; malignant	–	1	–	1	–	1
Sebaceous adenoma	1	–	–	–	–	–
Squamous carcinoma	–	–	–	1	–	–
Squamous papilloma	–	–	–	–	–	1
Mammary gland area	49	50	50	50	49	50
Adenocarcinoma	–	–	–	7	8	5
Adenoma	–	–	–	2	1	–
Fibroadenoma	–	–	–	13	16	14
Eyes	49	50	50	50	50	50
Local invasion	–	1	1	–	–	–
Harderian glands	49	50	50	50	50	50
Adenoma	–	–	1	–	–	–
Local invasion	–	1	1	–	1	1
Brain	50	50	50	49	50	50
Astrocytoma	–	–	–	–	–	1
Granular cell tumor; benign	1	2	–	1	–	–
Granular cell tumor; malignant	–	1	–	–	1	–
Local invasion	–	1	1	–	1	1
Meningioma; benign	–	–	1	–	–	–
Meningioma; malignant	–	1	–	–	–	–
Prostate gland	50	49	50	–	–	–
Adenoma	4	2	–	–	–	–
Seminal vesicles	50	49	50	–	–	–
Adenocarcinoma	–	1	–	–	–	–
Adenoma	1	–	–	–	–	–
Uterus	–	–	–	50	50	49
Endometrial stromal sarcoma	–	–	–	1	–	–
Endometrial stromal polyp	–	–	–	4	9	3
Endometrial adenoma	–	–	–	1	–	1
Fibroma	–	–	–	–	1	–
Local invasion	–	–	–	–	–	1
Schwannoma; malignant	–	–	–	1	–	1
Cervix	–	–	–	50	49	49
Local invasion	–	–	–	–	–	2
Vagina	–	–	–	48	47	46
Local invasion	–	–	–	–	–	2
Pituitary gland	50	50	50	50	50	50
Adenoma; pars anterior	31	25	29	27	31	34
Adenoma; pars interior	–	–	–	–	1	–
Carcinoma; pars anterior	–	1	–	–	1	1
Spinal cord	50	50	50	50	50	50
Astrocytoma	–	–	–	1	–	–
Bone, sternum	49	50	50	50	50	50
Local invasion	–	–	–	–	–	1
Preputial glands	1	1	1	–	–	–
Adenoma	–	1	–	–	–	–
Mediastinal tissue	1	–	–	1	–	–
Metastasis	–	–	–	1	–	–
Inguinal lymph nodes	–	–	–	2	–	–
Metastasis	–	–	–	1	–	–
Abdominal cavity	5	5	7	7	6	3
Liposarcoma	1	–	–	–	–	–
Metastasis	–	1	2	–	–	1

^a^Number of animals analyzed

^b^Number of animals with a particular tumor

## Discussion

The GM maize NK603 produced for the NK603 + Roundup diets to perform the three feeding trials was treated once with the glyphosate-containing herbicide Roundup Transorb^®^ HC as described in Materials and Methods following the principles of good agricultural practice and in conformity with the requirements of the KBBE-2013-FEEDTRIALS call “Two-year carcinogenicity rat feeding study with maize NK603”. During the final conference of the G-TwYST project, it was argued that the maize should have been exposed to much higher concentrations of the herbicide to induce toxic effects in the rats during the feeding trials, but this would have contradicted the principles of good agricultural practice. Moreover, it should be noted that the toxicity testing of glyphosate as such was not within the scope of the above-mentioned call. The toxicological evaluation of the active substance glyphosate by EFSA relied on a large number of studies (EFSA 2015). Glyphosate did not show a genotoxic potential and there was no evidence of carcinogenicity in rat or mice studies. Considering a number of long-term studies in rats, an overall long-term No Observed Adverse Effect Level (NOAEL) of 100 mg/kg body weight/day was obtained. Based on an overall maternal and developmental NOAEL of 50 mg/kg body weight/day obtained from several developmental toxicity studies in rabbits, an acceptable daily intake (ADI) value of 0.5 mg/kg body weight/day as well as an acute reference dose (ARfD) of 0.5 mg/kg body weight were derived (EFSA 2015).

The glyphosate levels in the near-isogenic non-GM maize (Pioneer 8906) kernels as well as in the untreated and Roundup-treated GM maize NK603 (Pioneer 8906 R) kernels were ≤ 16 µg/kg, which is far below the maximum residue level for glyphosate in maize (1 mg/kg) in the European Union (EU-Pesticides Database, accessed on the 5th of February 2019). All diets, including the control diet, were contaminated with low levels of glyphosate (about 30–140 µg/kg) not considered to be adverse. The source of these low levels of the contaminant is not known; they might derive from constituents in the diet other than the maize.

The choice of the rat strain for the G-TwYST feeding trials proved to be extremely important for the type of studies to be performed. In the study by Séralini et al. (2012), the Sprague–Dawley rat strain was chosen and a very high incidence of mammary gland tumors in female animals was reported. However, the female Sprague–Dawley rats are not suitable to demonstrate treatment-related mammary carcinogenesis because of the very high incidence of spontaneously formed mammary gland tumors (Weber 2017). In the present study, the incidence of mammary gland neoplasms in female rats fed the control diet was 44% (i.e. 7 adenocarcinomas, 2 adenomas and 13 fibroadenomas out of 50 rats). Therefore, the Wistar Han RCC rat strain used in the present study was a suitable choice to test if the GM maize NK603 had the ability to stimulate mammary gland carcinogenesis. It should be noted that the Wistar Han RCC rat strain has previously been used in numerous studies as an experimental model of chemically induced mammary gland carcinogenesis (e.g. Abd-Ellatef et al. 2017; Ganaie et al. 2017; Smina et al. 2017).

In the 90-day feeding trial with the GM maize NK603 at an inclusion rate of 11 and 33% in the diets, no adverse changes were observed regarding the parameters listed in the OECD Test Guideline 408 (OECD 1998) including the health status of the animals, the body weights, the haematological and clinical biochemical parameters, the relative organ weights as well as the necropsy and histopathology findings. Whilst some parameters showed statistically significant differences between the various NK603 maize-fed groups and the control group, “equivalence” or “equivalence more likely than not” could almost always be established for differences between the data of these groups and the historical control data. Moreover, the differences between test groups and the control group were generally small and/or not dose-related further indicating that they reflect normal variation and are not biologically relevant. Furthermore, there was no correlation between the haematological parameters, the clinical biochemical parameters and the relative organ weights showing significant differences and the necropsy and histopathology findings.

The T_4_ level was significantly lower in the sera of male rats fed the 33% NK603 diet and in the sera of female rats fed the 33% NK603 + Roundup diet if compared to the corresponding control groups, but there were no histopathological changes in the thyroid gland, so that these changes in the T_4_ levels are considered not to be adverse. The 17β-estradiol levels in the four groups of female rats fed the GM maize NK603 were significantly lower than that in the control group. However, these were not accompanied by histopathological alterations in any of the analyzed estrogen-sensitive tissues/organs, so that they are considered not to be adverse.

In the 90-day feeding trial with the GM maize NK603 at an inclusion rate of up to 50% in the diets, no adverse changes related to the feeding of the NK603 maize were observed regarding the parameters listed in the OECD Test Guideline 408 (OECD 1998) including the health status of the animals, the haematological and clinical biochemical parameters, the relative organ weights as well as the necropsy and histopathology findings. EFSA (2014) proposed an inclusion rate of 50% as a high maize dose in 90-day studies in rodents, based on a report by Zhu et al. (2013). In the present study, there was no indication of a nutritional imbalance if the maize inclusion rate was increased from 33 to 50% in a subchronic feeding trial, whereby in this case the diet composition had to be rebalanced. However, since it has been described that the subchronic and chronic feeding of high-protein diets leads to renal damage in rats (Rao 2002; Haseman et al. 2003; Wakefield et al. 2011; Aparicio et al. 2013), the inclusion rate of 33% maize in the diets was chosen by the G-TwYST consortium as the “high dose” of maize for the combined chronic toxicity/carcinogenicity study.

Mortality was significantly increased in male rats fed the 33% NK603 + Roundup diet when compared to the control group and was related to the increased number of deaths from pituitary neoplasia (12 male rats fed the control diet versus 17 male rats fed the 33% NK603 + Roundup diet), the most common cause of death in all groups including controls. However, it is important to mention that there was no effect of the 33% NK603 + Roundup diet on the overall incidence of pituitary neoplasia, i.e. the incidence of pituitary pars anterior adenomas in male rats fed the control and the 33% NK603 + Roundup diets was 62 and 58%, respectively. The increased mortality observed between the 12th and 24th month of the feeding trial in male rats fed the 33% NK603 + Roundup diet coincided with a strong increase in the body weight of these rats in this period of time. These results are in accordance with previous studies showing that ad libitum feeding of common high calorie diets to rodents in long-term studies results in higher body weights, an earlier onset and a higher incidence of spontaneous neoplasms such as pituitary gland tumors, as well as a reduced survival of these animals and that these effects are not observed in rats undergoing a dietary restriction (Keenan et al. 1995a, b; Roe et al. 1995; NTP 1997; Nold et al. 2001). Therefore, it is concluded that the increased mortality associated with a higher body weight and an increased incidence of pituitary neoplasms in male rats fed the 33% NK603 + Roundup diet is not specifically related to the feeding of this diet (Keenan et al. 1995a, b; Nold et al. 2001). In female rats fed the 33% NK603 + Roundup diet, the mortality rate was decreased when compared to the control female rats, but this difference was not statistically significant (*p* = 0.07). It should be noted that the overall incidence of pituitary neoplasms in control and GM maize NK603-fed male and female rats did not differ significantly.

The necropsy findings in the chronic toxicity and carcinogenicity phase of the 2-year feeding trial were considered to be within the normal range of background alterations seen in untreated animals of this age and strain and their incidence was similar in the control group and the groups fed the GM maize NK603. Moreover, the microscopic findings in the chronic toxicity and carcinogenicity phase of the 2-year feeding trial were consistent with spontaneously occurring findings described in the literature, the findings were distributed randomly among groups, and/or their appearance was similar to findings found in controls. The thymoma is a typical Wistar rat-related neoplasm, with females more affected than males (Murray et al. 1985; Poteracki and Walsh 1998; Weber 2017). The data obtained in the present study corroborate these earlier findings, whereby there was no statistically significant difference in the incidence of benign thymomas in the female control group and the female group fed the GM maize NK603 treated with Roundup at an inclusion rate of 33% or when benign and malignant thymomas were analyzed in combination. Based on the results obtained in the three feeding trials performed in the course of the G-TwYST project, it is concluded that there were no adverse findings related to the feeding with the NK603 or NK603 + Roundup diets to rats for up to 2 years.

In the study by Séralini et al. (2012), the most frequently occurring anatomical pathologies were observed in the kidneys, liver and digestive tract of male Sprague–Dawley rats and in the pituitary gland and mammary glands, including mammary tumors, in female Sprague–Dawley rats. Moreover, Séralini et al. (2012) indicated that the diets containing the untreated GM maize NK603 and the Roundup-treated GM maize NK603 contained significantly lower levels of the phenolic acids caffeic acid and ferulic acid than the control diet and hypothesised that this decrease could explain the higher tumor incidence observed in the GM maize NK603-fed rats due to less protection afforded by these compounds. In the present study, there were no statistically significant differences in the number of non-neoplastic and neoplastic findings in the above-mentioned organs/tissues between Wistar rats fed the control diet and those fed the GM maize NK603-containing diets. In line with these findings, the clinical biochemical parameters of hepatic (ALP, ALT, AST, TP and ALB) and renal function (CREA, UREA and ions) remained, with single exceptions, unaltered. Furthermore, the levels of caffeic acid were similar in the three batches of control and GM maize NK603 containing diets, and this was also the case of ferulic acid, whereby much higher levels of this compound were measured in batch 1 than in the batches 3 and 5 of all analyzed diets due to the different extraction methods applied by the two laboratories subcontracted to perform the analyses.

The protocols outlined in the OECD Test Guidelines 408 for subchronic toxicity testing and 453 for a combined chronic toxicity/carcinogenicity testing in rodents (OECD 1998, 2009) have been designed for the testing of single chemicals and provide standard procedures to identify health hazards resulting from the repeated exposure to chemicals for 90 days, 1 year and 2 years. The OECD Test Guidelines recommend the use of at least 10 animals/sex and group for subchronic toxicity testing, 20 animals/sex and group for chronic toxicity testing and at least 50 animals/sex and group for carcinogenicity testing. Chemicals can be administered individually to rodents at doses several multiples higher than the amount of the chemicals to which humans are exposed to test whether they may lead to toxicity. Whole food/feed contains a mixture of constituents and can only be administered to rodents at rather limited levels to avoid a nutritional imbalance. Therefore, it is unlikely that substances present in small amounts and with a low toxic potential in whole food/feed will cause any observable effects in animal feeding trials (EFSA GMO Panel Working Group on Animal Feeding Trials 2008).

The G-TwYST project has demonstrated that in principle, following the approach presented in van der Voet et al. (2017), reference groups of animals from previous similar trials in the same experimental facility fed with non-GM plant material considered to be safe may be used to define a regular bandwidth for each endpoint. In practice, it may be difficult to apply this approach, e.g. when the variability of analytical methods is not stable between the historical and current studies, such as for the differential white blood count data in this study, or when no historical data are available at all, as for the 2-year feeding trial. Traditional statistical tests applied in toxicological studies to find differences between test and control groups cannot make a distinction between biologically relevant and non-relevant effect sizes, and EFSA has recommended to put more emphasis on the use of confidence intervals (EFSA 2011c). Based on a confidence interval approach, equivalence tests can be used to show that a test group is within the bandwidth defined from historical data obtained in recent studies conducted in the same testing facility (“proof of safety”).

Equivalence bandwidths are ideally based on a large pool of historical data to capture the whole bandwidth of “safe” values. In this study, the available historical control database from the same test facility was limited with respect to the number of non-GM reference groups. This limitation leads to uncertainties, and to wider expected equivalence regions than would be obtained with a larger historical database. However, the consequences of limited data are taken into account in the equivalence tests, effectively by allowing larger differences to be equivalent. It may be noted that in many cases adverse effect limits are expected to lie far outside the equivalence region; thus, our approach is conservative and does not exclude toxic effects too easily.

An alternative approach is to define bandwidths using toxicologically defined quantifications of adverse effects. This was performed for nine endpoints, and was reported by Goedhart and van der Voet (2017, 2018a, f). However, tentative toxicologically defined bandwidths are often only available for a few measured endpoints in animal studies, and, therefore, an equivalence assessment based on historical data was preferred.

In the above-mentioned context, the non-GM data obtained in the course of the 90-day and 1-year rat feeding trials performed in the preceding GRACE project were used as historical control data for the equivalence testing in the G-TwYST project. G-TwYST proposed and applied a statistical method for equivalence testing that accounts for statistical uncertainties in both the current 90-day feeding trials and the chronic toxicity testing phase of the 2-year feeding trial as well as in the historical reference data (van der Voet et al. 2017; Goedhart and van der Voet 2017, 2018a, f). The analysis of the data from the rat feeding trials performed in the course of the G-TwYST project showed that the cases in which equivalence could not be concluded with a 95% statistical significance and the cases in which significant differences were observed correspond to no more than about 5% of cases across 1424 equivalence tests and 3472 difference tests, which is the expected percentage for statistical tests using a 5% error level. Moreover, most of the cases in which equivalence could not be concluded with a 95% statistical significance were related to a larger within-group variation of the measurements in the current studies as compared to the historical control data, which may be an analytical rather than a toxicological reason for a wide confidence interval.

G-TwYST performed a power analysis for quantitative findings in 90-day studies estimating effect sizes that could be estimated with 80% power (Goedhart and van der Voet 2018h). For parameters in the list of determinations as advocated by the OECD Test Guideline 408 for testing chemicals (OECD 1998) and the EFSA Guidance Document (EFSA 2011b), such as body/organ weights, haematology and clinical chemistry parameters, a design with 8 cages per group in a 90-day study is appropriate for more than 80% of the quantitative parameters if deviations of 1.3-fold (+ 30% or − 23%) or more have to be detected with at least 80% power.

However, the number of quantitative parameters as required to be measured by OECD (1998) is 40–50, and the within-group variability in these parameters was found in the G-TwYST studies to vary considerably, between 1% and 44% expressed as a coefficient of variation. The 10–20% of the parameters with a relatively high variability would require larger sample sizes to attain the same power as for the other parameters. In this study, we found relatively large variability for five parameters in both sexes (monocytes, eosinophils, BIL, TAG and HGB), and additionally for five parameters in female rats only (WBC, LYMA, Neutrophils, ALT and uterus weight) (Goedhart and van der Voet 2018h).

G-TwYST performed a whole food/feed combined chronic toxicity/carcinogenicity study with 50 animals/sex and group for the carcinogenicity phase, which is the minimal number requested by the OECD Test Guideline 453 (2009). A power analysis for qualitative (yes/no) findings, such as deaths and histopathological results, shows that with such a design only large differences in single incidences can be detected with at least 80% power (Goedhart and van der Voet 2018h). Increasing the number of animals is of limited help, and therefore, not a suggested direction for future animal studies. For example, in case that the control incidence is 1%, a ninefold rather than a 16-fold increase can be detected with 100 instead of 50 animals/sex and group, and, in the case that the control incidence is 10%, a 2.4-fold rather than a 3.2-fold increase can be detected. These levels of changes are often larger than what toxicologists would judge as minimal relevant values. The low sensitivity is inherent to the qualitative character of the findings. These considerations point at the limitations of using whole food/feed studies as an untargeted screening approach and underline the necessity to perform such studies with clearly targeted hypotheses. It is concluded that, apart from setting effect sizes of interest, a prior selection should be made of those parameters for which a sufficiently high power is needed, before a power analysis can be helpful to set the sample sizes for a 90-day animal study in line with the OECD Test Guideline 408 (1998) and EFSA documents (EFSA 2011b, 2014). The potential usefulness of such studies lies in the possibility to consider and interpret patterns of findings rather than in an analysis of single endpoints.

G-TwYST has confirmed that the variability of quantitative measurements in a combined chronic toxicity/carcinogenicity study is in general higher for the measurements after 12 and 24 months as compared to 3 or 6 months (Goedhart and van der Voet 2018a, g). Therefore, the statistical power to detect a certain difference decreases as the duration of the study increases, and larger samples may be needed for GM plant risk assessment based on 12 or 24 months data. In terms of clinical chemistry and haematology measures carried out at the 2-year termination period, scientific opinion is split as to their actual value since the concurrent appearance of age-related diseases, such as end-stage kidney and hormonal senescence, tends to confound the interpretation by increasing the variability in serum parameters associated with such effects (Young et al. 2011). For this reason, OECD TG 453 leaves the option of whether or not to carry out clinical chemistry and haematology measures after 24 months to the discretion of the study director (OECD 2009). Similar endpoints show less variability at the 12-month than at the 24-month termination point in time, but nevertheless do show increased variability when compared to those measured at the 90-day point in time due to the early appearance of age-related disease in some animals.

The G-TwYST project provided a broad set of data indicating that the performance of rat feeding trials with whole food/feed for the risk assessment of a GM plant did not result in the identification of hazards due to the GM maize NK603 and is absolutely in line with the earlier risk assessment published by EFSA (EFSA 2003). The obligatory risk assessment included, in addition to the detailed safety assessment of the newly introduced trait, a comparative assessment focusing on potential unintended effects of the genetic modification. This comparative assessment included: (1) a detailed molecular characterisation of the genetic modification, i.e. of the inserted sequence as well as of the place of insertion; (2) a phenotypic and agronomic comparison of the GM variety and its conventional comparator; (3) a compositional analysis of the GM variety in relation to its conventional counterpart as well as other non-GM varieties (reference varieties) (EFSA 2011b).

The 3Rs (Replacement, Reduction and Refinement) are guiding principles for a more ethical use of animals in laboratory experiments and were first described by Russell and Burch (1959). From the 3Rs perspective, it is particularly important to point out that the added value of long-term animal studies with whole food/feed should be carefully evaluated given the very high number of animals needed (in the case of the G-TwYST project: 720, 172 and 268 in the combined chronic toxicity/carcinogenicity study, the 90-day feeding trial with the 11 and 33% inclusion rates of the GM maize NK603 and the 90-day feeding trial with the 50% inclusion rate of the GM maize NK603, respectively).

In the original assessment of the GM maize NK603, there were no indications that the NK603 maize was not as safe as other maize varieties currently on the European market. As a result of this lack of hazards, there were no triggers, based on general toxicological principles, to perform additional animal feeding studies. Therefore, the G-TwYST 90-day and long-term animal studies were conducted in the absence of a specific hypothesis. The G-TwYST data from 90-day and long-term rodent feeding studies confirmed that there are no hazards, thereby supporting the results from the initial risk assessment performed by EFSA.

In conclusion, in the European GRACE and G-TwYST projects a series of animal feeding trials were performed (Zeljenková et al. 2014, 2016; this study). This series of studies neither delivered a scientific basis for the 90-day animal feeding trial demanded by the European Commission to be performed for each new GM plant variety nor did it indicate that untargeted, extended feeding studies with rats fed GM plant material are of value for a final confirmation of safety. Thus, an added value of animal studies relative to the available non-animal studies for the risk assessment of GM plants (EFSA Scientific Committee et al. 2017) was not substantiated.

## Electronic supplementary material

Below is the link to the electronic supplementary material.

Supplementary material 1 (DOCX 108 KB)
