# Supplementary material for: Lack of adverse effects in subchronic and chronic toxicity/carcinogenicity studies on the glyphosate-resistant genetically modified maize NK603 in Wistar Han RCC rats
Source: Arch Toxicol. 2019 Feb 12;93(4):1095–139. doi: 10.1007/s00204-019-02400-1 (PMC7261740; doi:10.1007/s00204-019-02400-1)
Supplement: Supplementary file 1 — Supplementary material 1 (DOCX 108 KB) [file 204_2019_2400_MOESM1_ESM.docx]

**Electronic Supplementary Material for:**

**Two subchronic toxicity studies and a combined chronic toxicity/carcinogenicity study on the genetically modified maize NK603 in Wistar Han RCC rats**

Pablo Steinberg^1,^*^,^**, Hilko van der Voet^2^, Paul W. Goedhart^2^, Gijs Kleter^3^, Esther J. Kok^3^, Maria Pla^4,5^, Anna Nadal^4^, Dagmar Zeljenková^6^, Radka Aláčová^6^, Júlia Babincová^6^, Eva Rollerová^6^, Soňa Jaďuďová^6^, Anton Kebis^6^, Elena Szabova^6^, Jana Tulinská^7^, Aurélia Líšková^7^, Melinda Takácsová^7^, Miroslava Lehotská Mikušová^7^, Zora Krivošíková^7^, Armin Spök^8,9^, Monica Racovita^9,^***, Huib de Vriend^10^, Roger Alison^11^, Clare Alison^11^, Wolfgang Baumgärtner^12^, Kathrin Becker^12^, Charlotte Lempp^12^, Marion Schmicke^13^, Dieter Schrenk^14^, Annette Pöting^15^, Joachim Schiemann^16^, Ralf Wilhelm^16^

^1^ Institute for Food Toxicology, University of Veterinary Medicine Hannover, Bischofsholer Damm 15, 30173 Hannover, Germany

^2^ Wageningen University & Research, Biometris, Droevendaalsesteeg 1, 6708PB Wageningen, The Netherlands

^3^ RIKILT Wageningen University & Research, Wageningen University & Research, Akkermaalsbos 2, 6708 WB Wageningen, The Netherlands

^4^ Institute of Food and Agricultural Technology, University of Girona, Campus Montilivi, 17003 Girona, Spain

^5^ CRAG-CSIC-IRTA-UAB-UB, Edifici CRAG, Campus UAB, 08193 Cerdanyola, Spain

^6^ Slovak Medical University, Faculty of Public Health, Limbová 12, 83303 Bratislava, Slovakia

^7^ Slovak Medical University, Faculty of Medicine, Limbová 12, 83303 Bratislava, Slovakia

^8^ Graz University of Technology, Schlögelgasse 2, A-8010 Graz, Austria

^9^ Alpen-Adria Universität Klagenfurt, Schlögelgasse 2, A-8010 Graz, Austria

^10^ LIS Consult, Hogesteeg 9, 3972 JS Driebergen, The Netherlands

^11^ Roger Alison Ltd., Lampeter, Ceredigion, United Kingdom

^12^ Department of Pathology, University of Veterinary Medicine Hannover, Bünteweg 17, Hannover, 30559, Germany

^13^ Clinic for Cattle, University of Veterinary Medicine Hannover, Bischofsholer Damm 15, 30173 Hannover, Germany

^14^ Food Chemistry and Toxicology, University of Kaiserslautern, Erwin-Schrödinger-Straße 52, 67663 Kaiserslautern, Germany

^15^ Federal Institute for Risk Assessment, Max-Dohrn-Straße 8-10, 10589 Berlin, Germany

^16^ Institute for Biosafety in Plant Biotechnology, Julius Kühn-Institut (JKI), Federal Research Centre for Cultivated Plants, Erwin-Baur-Str. 27, 06484 Quedlinburg, Germany

* Current address: Max Rubner-Institut, Federal Research Institute of Nutrition and Food, Haid-und-Neu-Str. 9, 76131 Karlsruhe, Germany

# ** To whom correspondence should be addressed at Max Rubner-Institut, Haid-und-Neu-Str. 9, 76131 Karlsruhe, Germany. Tel.: +49-721-6625-201. Fax: +49-721-6625-111. E-mail: pablo.steinberg@mri.bund.de

# *** Current address: Global Sustainability Institute, Anglia Ruskin University, East Road, CB1 1PT, Cambridge, UK

**Appendix 1. Additional details of statistical analysis**

This Appendix describes more details about the statistical methods applied to the data from the three feeding studies. A full description of all statistical methods and all results for the three studies, except for the histopathology, is given in the statistical reports (Goedhart and van der Voet 2017, 2018a-i), which can be accessed via the internet portal CADIMA.

11.1 Data preparation

Outlier detection was performed before the feeding group codes were unblinded. Grubbs’ outlier test at the 1% level was sequentially applied to the residuals after an analysis of variance (see below) on cage means for the two 90-day feeding trials and for the 3-, 6- and 12-month data in the combined chronic toxicity/ carcinogenicity study. For the 24-month data in the combined chronic toxicity/ carcinogenicity study, the outlier test was performed on individual animal data for those animals that survived after 24 months. This resulted in a number of outliers, which were first presented in the form of tables and graphs to the study director Dagmar Zeljenková and then to the G-TwYST coordinator Pablo Steinberg. Outliers were classified as either (1) typos and/or physiologically improbable values or (2) values that might be realistic. For the first category, the values were set to missing, thereby leading to the complete removal of the outliers. The analyses presented in this paper include the second category of outliers.

Note that in the five GRACE rat feeding trials a completely randomized design, i.e. without blocking, was used. The GRACE data were retrieved from the CADIMA website on the 29^th^ of November 2016. In the course of the GRACE project, five studies were conducted with diets containing several different non-GM maize varieties (Supplementary Electronic Material, Table 2; Schmidt et al [2017]).

The GRACE studies D and E only employed a single reference feed and, since the equivalence analysis corrects for differences between studies, these studies do not contribute to the between reference variation. In the GRACE study C, two reference feeds also used in GRACE study A were replicated. The degrees of freedom associated with the between reference feeds variance therefore equals 4. The degrees of freedom associated with the residual (between cages) variance varies between 50 and 78, since not all measurements were performed on all rats in each study.

11.2 Survival curves and mortality after 2 years

In the two 90-days feeding studies there was hardly any mortality. Survival and mortality were only investigated in the combined chronic toxicity/carcinogenicity study. Survival analysis was performed at the individual level, which disregards the fact that two animals were housed in the same cage, and at the cage level with ‘survival of a cage’ defined as survival of both animals in the cage.

Kaplan-Meier estimates of the survival curves were plotted for all animals/cages of a single sex for the five dose groups. The differences between the five dose groups were assessed using four overall tests for equality of survival curves (log-rank, Wilcoxon-Breslow, Tarone-Ware and Wilcoxon-Peto-Prentice), as defined in Collett (1994). In addition, survival was statistically analysed by means of the proportional odds model (Cox 1972) with a single survival function and with blocks and dose groups as treatment factors. Based on this model, pairwise differences between the control non-GM group and each of the GM maize NK603 test groups were assessed using a normal approximation for the logarithm of the hazard ratios. Since the interest is in increased mortality due to NK603 maize, one-sided testing was employed. Note that equal hazards correspond to a value of 0 on the log ratio scale. The survival analyses were performed using the procedures KAPLANMEIER, RSTEST, RPROPORTIONAL and RPHFIT and direct programming in GenStat 18 (VSN International 2015).

Whereas survival analysis considers the mortality pattern over time, a more restricted analysis can be done on the mortality rates at specific points in time. In such analyses, the exact time of death is not considered. The method used is described in the main text.

11.3 Growth curves and feed consumption

The observed weights were graphically displayed along with the fitted curve and the resulting estimate of the growth rate $\gamma$. In case that an absolute standardized residual was larger than 3, the accompanying weight was set to missing and the curve was fitted again. However, since the study laboratory had no explanation why these weights were outlying, the weights with the large residuals were retained.

11.4 Equivalence and difference tests of quantitative endpoints

For the historical non-GM GRACE data (Supplementary Electronic Material, Table 2), the following statistics were calculated: the within-study between reference feeds sums of squares ($SS_{R}$) and its degrees of freedom $df_{R}$, the residual sums of squares ($SS_{E}$) and degrees of freedom $df_{E}$, and finally the effective unit replication $n_{eff}$, which is necessary to estimate the between reference variance using the mean squares for feeds and for residuals. The equivalence test also required for the G-TwYST studies estimates of differences between the GM maize containing feeds and the control feed as well as the residual sums of squares and the associated degrees of freedom resulting from an analysis of variance.

The equivalence limit $\theta_{0}$ for the DWE criterion is only based on the design values of the historical studies and on three regulatory values: the minimal regulatory sample size $n_{0}$, a probability $\alpha$ which defines a $100\left( 1-\alpha\right)$% confidence interval for a difference between two feeding groups in the current study, and a probability $\beta$ which defines the desired power $1-\beta$ for the equivalence test. We used $\alpha=0.05$ and $\beta=0.05$, resulting in a power of 0.95. For the two 90-day feeding trials, we used a regulatory sample size $n_{0}=8$, which equals the replication, i.e. the number of cages, for most variables in both the GRACE studies and the two G-TwYST 90-day feeding trials. For the combined chronic toxicity/carcinogenicity study, the regulatory sample size was set to the level of replication in that study, which is different for different endpoint groups. Furthermore, the equivalence limit $\theta_{0}$ is calculated by simulating a large number of datasets in a simplified situation, where for each dataset an upper $100\left( 1-\alpha\right)$% percentile, $\theta_{upp}^{0}$, for the DWE criterion is approximated by a large number of so-called GPQ samples. We simulated 40,000 datasets with 15,000 GPQ samples for each dataset. Note that $\theta_{0}$ is calculated as the upper $100\left( 1-\beta\right)\%$ percentile of the thus obtained 40.000 values of $\theta_{upp}^{0}$. The DWE criterion for the current dataset was approximated by means of 100,000 GPQ samples. Note that the equivalence limit $\theta_{0}$ is calculated assuming a regulatory sample size $n_{0}=8$ in the two 90-day feeding trials, which implies 14 (regulatory) degrees of freedom for residual in the current study. The current two 90-day feeding trials indeed have replication 8, but more degrees of freedom for residual (28 for the 90-day feeding trial with a GM maize inclusion rate of 11 and 33% and 49 for the 90-day feeding trial with a GM maize inclusion rate of up to 50%). For the combined chronic toxicity/carcinogenicity study, these numbers for degrees of freedom depend on the endpoint group.

For further interpretation, the ELSD confidence intervals are also presented in the statistical report (Goedhart and van der Voet 2017, 2018bcdef) at the original ratio scale, with inclusion of the estimated equivalence limits (red bars) and their uncertainty (blue bars). Note that the latter graphs cannot be used directly for performing the equivalence test. However, they show the effects and equivalence limits at a more familiar scale.

The model for difference testing was fitted by means of linear regression, using the lm() function in R, because this takes proper account of any missing values. The ANOVA residuals were assessed by means of normal probability plots and plots of residuals versus fitted values. The usual summary statistics are saved as well as estimates for the difference between GMO feeds and the control feed and corresponding standard errors, t-values and *p*-values. These are all calculated using the pooled ANOVA residual standard error. The residuals of the analysis of variance are checked for normality using the Kolmogorov-Smirnov test as well as the Shapiro-Wilk test for normality. These tests are only approximate, since the residuals are not independent. The *p*-value of the Kolmogorov-Smirnov test is not reported, since it is almost always larger that the *p*-value of the Shapiro-Wilk test, moreover for variables, where it is smaller, the *p*-value is far from significant. In addition to the t-tests, Dunnett’s test was performed, which also compares the GM maize containing feeds with the control feed, taking account of the number of comparisons made. Dunnett’s test was performed by means of the glht() function in the multcomp R-package. Non-parametric Wilcoxon signed rank tests were used to test for a difference between each GM maize containing feed and the control feed. Note that this test only uses data of these two feeds and that the test employs the within block difference between the GM maize containing diet and the control feed. The *p*-value of the test was calculated by means of the wilcox.test() function in R, which calculates exact probabilities. The non-parametric Friedman test, which is applicable to a randomized block design, was used to test for overall differences between the five feeds. Homogeneity of variance was assessed by means of Bartlett’s test and by means of Levene’s test, both using the mean and the median. These tests do not take blocking into account and basically compare the within feed variances. Note that homogeneity of variance was already assessed by means of a plot of residuals versus fitted values. The *p*-value of the Levene test with the median is not reported, since it is almost always larger that the *p*-value of the Levene test with the mean, and for variables, where it is smaller, the *p*-value is far from significant. Note that both analysis of variance and non-parametric tests require homogeneity of variance. Finally, for each feeding group separately, normality was assessed by means of the Kolmogorov-Smirnov test and the Shapiro-Wilk test. The *p*-value of the Kolmogorov-Smirnov test is always larger than 5% and is thus not reported.

For the oestrous cycle data in the 90-day feeding trial with a GM maize NK603 inclusion rate of 11 and 33%, Fisher’s exact test was used for testing whether the probability of having an unfinished cycle was different between the feeds, and whether the probability of having a cycle of 5 days was different between the feeds. Logistic regression was used for testing whether the probability of having an irregular cycle was different between the feeds.

**Table 1:** Composition of the diets used in the G-TwYST rat feeding trials (components in % w/w)

| **Maize inclusion rate in the diet** | **33.0** | **50.0** |
| --- | --- | --- |
| Wheat | 22.7 | 25.0 |
| Wheat middlings | 25.0 | 5.0 |
| Soybean meal (48% protein) | 5.0 | 5.0 |
| Soybean oil | 3.0 | 2.7 |
| Rice protein concentrate | 5.5 | 6.5 |
| Brewers yeast | 1.0 | 1.0 |
| Vitamins, salts, minerals, etc. | 4.9 | 5.1 |

**Table 2:** Non-GM maize varieties used in the five feeding trials performed in the course of the EU-funded project GRACE

| **GRACE feeding trial** | **Control diet** | **33% Conv-1 diet** | **33% Conv-2 diet** |
| --- | --- | --- | --- |
| **A** | DKC6666^1^ | PR33W82^2^ | SY-NEPAL^3^ |
| **B** | PR32T16^2^ | PR32T83^2^ | DKC6815^1^ |
| **C** | DKC6666 | - | SY-NEPAL |
| **D** | DKC6666 | - | - |
| **E** | PR32T16 | - | - |

1. From Monsanto
2. From Pioneer Hi-Bred
3. From Koipesol Semillas

| **Parameter** | **33% Control^1^** | | | **11% NK603^2^** | | | **33% NK603^3^** | | | **11% NK603 + Roundup^4^** | | | **33% NK603 + Roundup^5^** | | |
| --- | --- | --- | --- | --- | --- | --- | --- | --- | --- | --- | --- | --- | --- | --- | --- |
|  | **Batch 1** | **Batch 3** | **Batch 5** | **Batch 1** | **Batch 3** | **Batch 5** | **Batch 1** | **Batch 3** | **Batch 5** | **Batch 1** | **Batch 3** | **Batch 5** | **Batch 1** | **Batch 3** | **Batch 5** |
| **NK603 event (% of maize)** | not detected | detected, n.q.^6^ | detected, n.q. | 27% | 22% | 26% | 79% | 68% | 67% | 33% | 32% | 32% | 95% | 84% | 108% |
| **Glyphosate (ppb)** | 29 | 140 | 36 | 29 | 140 | 34 | 34 | 120 | 31 | 30 | 140 | 39 | 36 | 130 | 43 |

**Table 3:** NK603 event and glyphosate levels in the diets used for the 90-day feeding trial with GM maize NK603 inclusion rates of 11 and 33% and for the combined chronic toxicity/carcinogenicity feeding trial

^1^ 33% Pioneer 8906 (near-isogenic non-GM comparator)

^2^ 22% Pioneer 8906 + 11% Pioneer 8906 R (GM maize NK603)

^3^ 33% Pioneer 8906 R

^4^ 22% Pioneer 8906 + 11% Pioneer 8906 R treated once with Roundup during cultivation

^5^ 33% Pioneer 8906 R treated once with Roundup during cultivation

^6^ n.q., not quantifiable

**Table 4:** NK603 event and glyphosate levels in the diets used for the 90-day feeding trial with GM maize NK603 inclusion rates of 11, 33 and 50%

| **Parameter** | **33% Control^1^**  **(33% maize *in toto*)** | **33% NK603^2^**  **(33% maize *in toto*)** | **33% NK603 + Roundup^3^**  **(33% maize *in toto*)** | **50% Control maize^4^**  **(50% maize *in toto*)** | **11% NK603^5^**  **(50% maize *in toto*)** | **50% NK603^6^**  **(50% maize *in toto*)** | **11% NK603 + Roundup^7^**  **(50% maize *in toto*)** | **50% NK603 + Roundup^8^**  **(50% maize *in toto*)** |
| --- | --- | --- | --- | --- | --- | --- | --- | --- |
| **NK603 event (% of maize)** | not determined | not determined | not determined | detected,  not quantifiable | 16 | 64 | 23 | 94 |
| **Glyphosate (ppb)** | 31 | not determined | 36 | < 10 | < 10 | < 10 | < 10 | < 10 |

^1^ 33% Pioneer 8906 (near-isogenic non-GM comparator)

^2^ 33% Pioneer 8906 R (GM maize NK603)

^3^ 33% Pioneer 8906 R treated once with Roundup during cultivation

^4^ 50% Pioneer 8906

^5^ 39% Pioneer 8906 + 11% Pioneer R

^6^ 50% Pioneer 8906 R

^7^ 39% Pioneer 8906 + 11% Pioneer 8906 R treated once with Roundup during cultivation

^8^ 50% Pioneer 8906 R treated once with Roundup during cultivation

**Table 5:** Necropsy findings in male and female Wistar Han RCC rats in the 90-day feeding trial with GM maize NK603 at an inclusion rate of 11 and 33% in the diet

| **Group** | **Isogenic maize**  **(% of diet)** | **NK603 only**  **(% of diet)** | **NK603 +**  **Roundup**  **(% of diet)** | **No. of**  **Males** | **No. of**  **Females** |
| --- | --- | --- | --- | --- | --- |
| **1** | 33 | 0 | 0 | 16 | 16 |
| **2** | 22 | 11 | 0 | 16 | 16 |
| **3** | 0 | 33 | 0 | 16 | 16 |
| **4** | 22 | 0 | 11 | 16 | 16 |
| **5** | 0 | 0 | 33 | 16 | 16 |

—————————————————————————————————————————————————————————————————————————

NUMBER OF ANIMALS WITH MACROSCOPIC FINDINGS BY ORGAN/GROUP/SEX

—————————————————————————————————————————————————————————————————————————

SEX : MALE FEMALE

DOSE GROUP: 1 2 3 4 5 1 2 3 4 5

—————————————————————————————————————————————————————————————————————————

MANDIBULAR LYMPH NODE

— Discoloration, red : 5 8 7 5 4 5 2 3 5 5

—————————————————————————————————————————————————————————————————————————

SKELETAL MUSCLE

- Deformation : 1 - - - - - - - - -

- Nodule : 1 - - - - - - - - 1

—————————————————————————————————————————————————————————————————————————

BRAIN

- Deformation : 1 - - - - - - - - -

—————————————————————————————————————————————————————————————————————————

KIDNEY

- Discoloration, white: 1 - - - - - - - - -

- Discoloration, red : 1 3 3 6 - - - 1 - -

—————————————————————————————————————————————————————————————————————————

HEART

- Discoloration, red : 1 - - - - - - - - -

—————————————————————————————————————————————————————————————————————————

LUNG

- Discoloration, red : 2 1 3 3 2 - 1 2 5 1

- Discoloration, white: - 3 1 3 - 2 2 1 1 -

- Nodule : - - - - - - - 1 - -

—————————————————————————————————————————————————————————————————————————

THYMUS

- Discoloration, red : - 1 2 4 - 3 1 1 1 1

- Discoloration, brown: - - 1 - - - - - - -

—————————————————————————————————————————————————————————————————————————

JEJUNUM

- Discoloration, red : - 1 - 1 1 1 - - 1 2

—————————————————————————————————————————————————————————————————————————

ABDOMINAL CAVITY

- Nodule : - - - - - 1 - 1 - -

—————————————————————————————————————————————————————————————————————————

SEMINAL VESICLE

- Nodule : - 1 - - -

—————————————————————————————————————————————————————————————————————————

COAGULATING GLANDS

- Nodule : - 1 - - -

—————————————————————————————————————————————————————————————————————————

URINARY BLADDER

- Discoloration, red : - 1 1 2 - - - - - -

—————————————————————————————————————————————————————————————————————————

—————————————————————————————————————————————————————————————————————————

CECUM

- Discoloration, red : - 1 - - - - - - - -

—————————————————————————————————————————————————————————————————————————

STOMACH

- Discoloration, black: - - - - - - 1 - - -

- Discoloration, red : - - 1 - 1 - - - - -

—————————————————————————————————————————————————————————————————————————

PANCREAS

- Discoloration, white: - - - - - - 1 - - 1

—————————————————————————————————————————————————————————————————————————

SPINAL CORD

- Discoloration, red : - - 1 - - - - - - -—————————————————————————————————————————————————————————————————————————

LIVER

- Enlargement : - - 1 - - - - - - -

—————————————————————————————————————————————————————————————————————————

SPLEEN

- Enlargement : - - 1 - - - - - - -

- Discoloration, white: - - 1 - 1 - - - - -

—————————————————————————————————————————————————————————————————————————

TRACHEA

- Fluid, red : - - - - 1 - - - - -

—————————————————————————————————————————————————————————————————————————

BONE

- Mass : - - - - - - - - - 1

—————————————————————————————————————————————————————————————————————————

**Table 6:** Microscopic findings in male Wistar Han RCC rats in the 90-day feeding trial with GM maize NK603 at an inclusion rate of 11 and 33% in the diet

| **Group** | **Isogenic maize**  **(% of diet)** | **NK603 only**  **(% of diet)** | **NK603 +**  **Roundup**  **(% of diet)** | **No. of**  **Males** | **No. of**  **Females** |
| --- | --- | --- | --- | --- | --- |
| **1** | 33 | 0 | 0 | 16 | 16 |
| **2** | 22 | 11 | 0 | 16 | 16 |
| **3** | 0 | 33 | 0 | 16 | 16 |
| **4** | 22 | 0 | 11 | 16 | 16 |
| **5** | 0 | 0 | 33 | 16 | 16 |

————————————————————————————————————————————————————

DOSE GROUP: 1 2 3 4 5

————————————————————————————————————————————————————

SYSTEMIC NEOPLASM : - - 1 - -

— Malignant lymphoma : - - 1 - -

————————————————————————————————————————————————————

HEART : 16 - 16 - 16

- Cardiomyopathy : 6 - 2 - 6

Grade 1: 6 - 2 - 6

————————————————————————————————————————————————————

AORTA : 16 - 15 - 16

————————————————————————————————————————————————————

ESOPHAGUS : 16 - 15 - 16

————————————————————————————————————————————————————

TRACHEA : 16 - 16 - 16

— Blood in lumen : - - - - 1

————————————————————————————————————————————————————

LIVER : 16 - 16 - 16

- Infl. cell foci : 12 - 14 - 12

Grade 1: 11 - 13 - 10

Grade 2: 1 - 1 - 2

- Hematopoiesis : 1 - - - 2

Grade 1: 1 - - - 2

- Necrosis; focal : 1 - - - -

Grade 1: 1 - - - -

- Vacuolation; PP : 4 - 1 - 3

Grade 1: 4 - 1 - 3

- Vacuolation; diffuse: - - 1 - -

Grade 2: - - 1 - -

- Necrosis;single cell: 2 - - - 3

Grade 1: 2 - - - 3

- Altered focus; eosin: 1 - - - -

Grade 1: 1 - - - -

- SYSTEMIC NEOPLASM : - - 1 - -

Grade 4: - - 1 - -

————————————————————————————————————————————————————

SPLEEN : 16 - 16 - 16

- Hematopoiesis : 16 - 15 - 16

Grade 1: 2 - 3 - 3

Grade 2: 11 8 7

Grade 3: 3 4 6

- Hemosiderin : 16 - 14 - 16

Grade 1: 15 - 9 - 14

Grade 2: 1 - 5 - 2

- SYSTEMIC NEOPLASM : - - 1 - -

Grade 4: - - 1 - -

————————————————————————————————————————————————————

————————————————————————————————————————————————————

MESENT. LYMPH NODE : 15 - 16 - 16

- Ceroid : 3 - 2 - 2

Grade 1: 3 - 2 - 2

- Dilation; sinusoid : - - 1 - 1

Grade 1: - - 1 - -

Grade 2: - - - - 1

- Erythrocytosis : 1 - 1 - 1

Grade 1: 1 - 1 - 1

- SYSTEMIC NEOPLASM : - - 1 - -

Grade 2: - - 1 - -

————————————————————————————————————————————————————

KIDNEYS : 16 3 16 6 16

- Cyst; cortex : 1 - - - -

Grade 2: 1 - - - -

- Hemorrhage; agonal : 1 3 2 4 -

- Hyaline droplets : 1 - 2 - 3

Grade 1: 1 - 2 - 1

Grade 2: - - - - 2

- Tubular basophilia : 1 - - - 1

Grade 1: 1 - - - 1

- Tubular casts : - - 1 1 -

Grade 1: - - 1 1 -

- SYSTEMIC NEOPLASM : - - 1 - -

Grade 1: - - 1 - -

————————————————————————————————————————————————————

URINARY BLADDER : 16 1 16 2 16

- Congestion : - 1 - - -

Grade 1: - 1 - - -

————————————————————————————————————————————————————

DUODENUM : 15 - 16 - 16

————————————————————————————————————————————————————

JEJUNUM : 16 1 16 1 16

- Hemorrhage : - - - - 1

Grade 1: - - - - 1

————————————————————————————————————————————————————

ILEUM : 16 - 16 - 15

————————————————————————————————————————————————————

G.A.L.T. : 14 - 14 - 15

- Lipid : 2 - 2 - -

Grade 1: 2 - 2 - -

- Mineralisation : - - 1 - -

Grade 1: - - 1 - -

————————————————————————————————————————————————————

CECUM : 16 1 16 - 16

————————————————————————————————————————————————————

COLON : 16 - 16 - 16

————————————————————————————————————————————————————

RECTUM : 16 - 16 - 16

————————————————————————————————————————————————————

STOMACH : - - 1 - 1

————————————————————————————————————————————————————

FORESTOMACH : 16 - 16 - 16

————————————————————————————————————————————————————

GLANDULAR STOMACH : 16 - 16 - 16

- Dilated glands : 2 - 1 - -

Grade 1: 2 - 1 - -

- Inflammation; submuc: 3 - 1 4 1

Grade 1: 1 - 1 - 1

Grade 2: 2 - - - -

- Ectopic squamous ep.: 1 - - - -

Grade 1: 1 - - - -

————————————————————————————————————————————————————

————————————————————————————————————————————————————

LUNG : 16 3 16 4 16

- Alveolar macrophages: 7 3 6 2 5

Grade 1: 5 - 1 - 3

Grade 2: 2 3 5 2 2

- Agonal hemorrhage : 1 - 1 - 2

Grade 1: - - 1 - 1

Grade 2: 1 - - - 1

- Congestion : 1 1 3 2 1

Grade 1: 1 1 3 2 1

————————————————————————————————————————————————————

THYMUS : 16 1 16 4 16

Agonal hemorrhage : 1 1 4 4 1

Grade 1: 1 - 2 3 1

Grade 2: - 1 2 - -

Grade 3: - - - 1 -

- SYSTEMIC NEOPLASM : - - 1 - -

Grade 2: - - 1 - -

- Atrophy; lymphoid : - - 1 - -

Grade 1: - - 1 - -

————————————————————————————————————————————————————

TESTES : 16 - 16 - 16

- Sertoli C. Vacuolat.: 1 - 2 - -

Grade 1: 1 - 2 - -

- Tubular atrophy;foc.: 1 - - - -

Grade 1: 1 - - - -

————————————————————————————————————————————————————

EPIDIDYMIDES : 16 - 16 - 16

————————————————————————————————————————————————————

MANDIBULAR GLANDS : 16 - 16 - 16

————————————————————————————————————————————————————

SUBLINGUAL GLANDS : 16 - 16 - 16

————————————————————————————————————————————————————

MANDIB. LYMPH NODES : 16 8 16 5 16

- Erythrocytosis : 6 7 6 5 5

Grade 1: 5 7 5 4 4

Grade 2: 1 - 1 1 1

Grade 3: - - - - -

- Incr; plasma cells : 1 2 1 2 1

Grade 1: 1 - 1 2 -

Grade 2: - 2 - - 1

- SYSTEMIC NEOPLASM : - - 1 - -

Grade 2: - - 1 - -

————————————————————————————————————————————————————

PANCREAS : 16 - 15 - 16

- Apoptosis : 7 - 6 - 4

Grade 1: 4 - 3 - 2

Grade 2: 3 - 3 - 2

- Atrophy;acin.cel.foc: 5 - 1 - 2

Grade 1: 5 - - - 2

Grade 2: - - 1 - -

- Basophilia; focal : 1 - - - -

Grade 1: 1 - - - -

- SYSTEMIC NEOPLASM : - - 1 - -

Grade 2: - - 1 - -

————————————————————————————————————————————————————

THYROID GLANDS : 16 - 15 - 14

————————————————————————————————————————————————————

PARATHYROID GLANDS : 4 - 5 - 5

————————————————————————————————————————————————————

————————————————————————————————————————————————————

ADRENAL CORTICES : 16 - 16 - 16

- Extracaps.cort.tiss.: - - 3 - 2

- Vacuolation : 1 - 2 - 3

Grade 1: - - 2 - 3

Grade 2: 1 - - - -

- SYSTEMIC NEOPLASM : - - 1 - -

Grade 1: - - 1 - -

————————————————————————————————————————————————————

ADRENAL MEDULLAS : 16 - 16 - 16

————————————————————————————————————————————————————

SKELETAL MUSCLE : 16 - 16 - 16

- Infl. cell foci : 2 - - - 2

Grade 1: 2 - - - 2

- Granuloma : 1 - - - -

Grade 3: 1 - - - -

————————————————————————————————————————————————————

TONGUE : 16 - 16 - 16

- Infl. cell foci : 1 - - - -

Grade 1: 1 - - - -

————————————————————————————————————————————————————

SCIATIC NERVE : 16 - 16 - 16

————————————————————————————————————————————————————

SKIN/SUBCUTIS : 16 - 16 - 16

————————————————————————————————————————————————————

MAMMARY GLAND AREA : 16 - 16 - 16

————————————————————————————————————————————————————

EYES : 16 - 16 - 16

- Damaged : - - 1 - -

- Atrophy; retina : - - - - 1

Grade 2: - - - - 1

————————————————————————————————————————————————————

HARDERIAN GLANDS : 16 - 16 - 16

- Infiltrate; lymphoid: 1 - 2 - -

Grade 1: 1 - 2 - -

- Pigment; brown : 1 - 2 - 2

Grade 1: - - 1 - 2

Grade 2: - - 1 - -

————————————————————————————————————————————————————

LACRIMAL GLANDS : 16 - 16 - 16

- Inflamm./necrosis : - - - - 1

Grade 1: - - - - 1

- Degeneration/inflamm: - - 1 - -

Grade 1: - - 1 - -

- Metaplasia/Harderian: 6 - 3 - 6

Grade 1: 5 - 2 - 5

Grade 2: 1 - 1 - 1

- Infiltrate/lymphoid : - - 1 - 1

Grade 3: - - 1 - 1

————————————————————————————————————————————————————

BRAIN : 16 - 16 - 16

————————————————————————————————————————————————————

PROSTATE GLAND : 16 - 16 - 16

- Infiltrate/lymphoid : 3 - 2 - -

Grade 1: 1 - 2 - -

Grade 2: 1 - - - -

Grade 3: 1 - - - -

- Infiltrate/ neutroph: - - 1 - -

Grade 3: - - 1 - -

————————————————————————————————————————————————————

————————————————————————————————————————————————————

SEMINAL VESICLES : 16 1 16 - 16

- Infiltrate/ mixed : - - 1 - -

Grade 2: - - 1 - -

————————————————————————————————————————————————————

COAGULATING GLANDS : 15 1 16 - 16

- Infiltrate/ mixed : - - 1 - -

Grade 2: - - 1 - -

————————————————————————————————————————————————————

PITUITARY GLAND : 16 - 16 - 16

- Cyst/Pars Anterior : 1 - 1 - 1

- Vacuolated cells/PA : 2 - - - -

Grade 1: 2 - - - -

- SYSTEMIC NEOPLASM : - - 1 - -

Grade 1: - - 1 - -

————————————————————————————————————————————————————

SPINAL CORD : 16 - 16 - 16

- Hemorrhage; agonal : - - - - 1

————————————————————————————————————————————————————

BONE, STERNUM : 16 - 16 - 16

————————————————————————————————————————————————————

BONE MARROW, STERNUM : 16 - 16 - 16

- SYSTEMIC NEOPLASM : - - 1 - -

Grade 3: - - 1 - -

————————————————————————————————————————————————————

FEMUR/JOINT : 16 - 16 - 16

- SYSTEMIC NEOPLASM : - - 1 - -

Grade 2: - - 1 - -

————————————————————————————————————————————————————

Infl., inflammatory; eosin, eosinophilic cell type; PP, periportal; G.A.L.T., gut associated lymphoid tissue; submuc, submucosal; ectopic squamous ep, ectopic squamous epithelium; Sertoli C. Vacuolat., Sertoli cell vacuolation; foc., focal; mandib., mandibular; incr., increased cellularity; acin.cel.foc, acinar cell, focal; extracaps.cort.tiss., extracapsular cortical tissue; inflamm., inflammation; neutroph, neutrophil; PA, Pars Anterior

**Table 7:** Microscopic findings in female Wistar Han RCC rats in the 90-day feeding trial with GM maize NK603 at an inclusion rate of 11 and 33% in the diet

| **Group** | **Isogenic maize**  **(% of diet)** | **NK603 only**  **(% of diet)** | **NK603 +**  **Roundup**  **(% of diet)** | **No. of**  **Males** | **No. of**  **Females** |
| --- | --- | --- | --- | --- | --- |
| **1** | 33 | 0 | 0 | 16 | 16 |
| **2** | 22 | 11 | 0 | 16 | 16 |
| **3** | 0 | 33 | 0 | 16 | 16 |
| **4** | 22 | 0 | 11 | 16 | 16 |
| **5** | 0 | 0 | 33 | 16 | 16 |

————————————————————————————————————————————————————

DOSE GROUP: 1 2 3 4 5

————————————————————————————————————————————————————

HEART : 16 - 16 - 16

- Cardiomyopathy : - - - - 2

Grade 1: - - - - 2

————————————————————————————————————————————————————

AORTA : 16 - 15 - 16

————————————————————————————————————————————————————

ESOPHAGUS : 16 - 16 - 16

————————————————————————————————————————————————————

TRACHEA : 16 - 16 - 16

————————————————————————————————————————————————————

LIVER : 16 - 16 - 16

- Infl. cell foci : 10 - 9 - 14

Grade 1: 10 - 9 - 13

Grade 2: - - - - 1

- Hematopoiesis : 1 - 1 - 1

Grade 1: 1 - 1 - 1

- Vacuolation; PP : 1 - - - -

Grade 1: 1 - - - -

- Vacuolation; diffuse: - - 1 - -

Grade 2: - - 1 - -

- Necrosis;single cell: - - - - 1

Grade 1: - - - - 1

- Pigment; hepatocytes: 1 - 1 - 3

Grade 2: 1 - - - -

Grade 3: - - 1 - 3

- Pigment/ macrophages: - - 1 - 3

Grade 2: - - 1 - 3

————————————————————————————————————————————————————

SPLEEN : 16 - 16 - 16

- Hematopoiesis : 16 - 15 - 16

Grade 1: 1 - 1 - 1

Grade 2: 4 4 6

Grade 3: 11 10 9

- Hemosiderin : 15 - 15 - 16

Grade 1: 11 - 10 - 10

Grade 2: 4 - 5 - 6

————————————————————————————————————————————————————

MESENT. LYMPH NODE : 16 - 16 - 16

- Ceroid : 2 - - - -

Grade 1: 2 - - - -

- Dilation; sinusoid : - - - - 1

Grade 1: - - - - 1

- Erythrocytosis : - - 2 - -

Grade 1: - - 1 - -

Grade 2: - - 1 - -

————————————————————————————————————————————————————

————————————————————————————————————————————————————

KIDNEYS : 16 - 16 - 16

- Hemorrhage; agonal : - - 1 - -

- Infiltrate; lymphoid: 1 - 1 - -

Grade 1: 1 - 1 - -

- Tubular basophilia : - - 1 - -

Grade 1: - - 1 - -

- Tubular casts : - - - - 3

Grade 1: - - - - 3

————————————————————————————————————————————————————

URINARY BLADDER : 16 - 16 - 16

————————————————————————————————————————————————————

DUODENUM : 16 - 16 - 16

————————————————————————————————————————————————————

JEJUNUM : 16 - 16 1 16

- Hemorrhage : - - - 1 1

Grade 2: - - - 1 -

Grade 3: - - - - 1

————————————————————————————————————————————————————

ILEUM : 16 - 16 - 15

————————————————————————————————————————————————————

G.A.L.T. : 8 - 9 - 9

- Lipid : - - 1 - -

Grade 1: - - 1 - -

————————————————————————————————————————————————————

CECUM : 16 - 16 - 16

————————————————————————————————————————————————————

COLON : 16 - 16 - 16

————————————————————————————————————————————————————

RECTUM : 16 - 16 - 16

————————————————————————————————————————————————————

FORESTOMACH : 16 - 16 - 16

————————————————————————————————————————————————————

GLANDULAR STOMACH : 16 1 16 - 16

- Dilated glands : 1 - 1 - -

Grade 1: 1 - 1 - -

- Degeneration; glands: - - 1 - 2

Grade 1: - - 1 - 2

- Erosion : - - 1 - -

Grade 1: - - 1 - -

- Hemorrhage : - 1 - - -

Grade 1: - 1 - - -

————————————————————————————————————————————————————

LUNG : 16 3 16 5 16

- Alveolar macrophages: 5 2 5 3 5

Grade 1: 1 - 1 1 5

Grade 2: 4 2 4 2 -

- Agonal hemorrhage : - - 1 2 -

Grade 1: - - 1 2 -

Grade 2: - - - - -

- Alveolar bone : - - 1 - -

Grade 1: - - 1 - -

- Congestion : - 1 1 2 -

Grade 1: - 1 1 2 -

————————————————————————————————————————————————————

THYMUS : 16 1 16 1 16

Agonal hemorrhage : 4 1 1 1 3

Grade 1: 2 - 1 - 1

Grade 2: 2 1 - 1 2

- Atrophy; lymphoid : - - - - 2

Grade 1: - - - - 2

————————————————————————————————————————————————————

————————————————————————————————————————————————————

OVARIES : 16 - 16 - 16

- Corpora lutea : 16 - 16 - 16

- Cyst; luteal : - - 1 - 1

————————————————————————————————————————————————————

MANDIBULAR GLANDS : 16 - 16 - 16

————————————————————————————————————————————————————

SUBLINGUAL GLANDS : 16 - 15 - 16

————————————————————————————————————————————————————

MANDIB. LYMPH NODES : 16 2 16 5 15

- Erythrocytosis : 5 2 4 3 2

Grade 1: 3 2 3 1 2

Grade 2: 2 - - 2 -

Grade 3: - - 1 - -

- Incr; plasma cells : 1 - - - 2

Grade 1: 1 - - - 1

Grade 2: - - - - 1

- Congestion : - - - - 1

Grade 2: - - - - 1

————————————————————————————————————————————————————

PANCREAS : 16 1 16 - 16

- Apoptosis : 2 - 3 - -

Grade 1: 1 - 2 - -

Grade 2: 1 - 1 - -

- Atrophy;acin.cel.foc: 1 - 3 - -

Grade 1: 1 - 2 - -

Grade 2: - - 1 - -

- Infiltrate; mononuc : - - 1 - -

Grade 1: - - 1 - -

————————————————————————————————————————————————————

THYROID GLANDS : 15 - 14 - 15

————————————————————————————————————————————————————

PARATHYROID GLANDS : 11 - 10 - 7

————————————————————————————————————————————————————

ADRENAL CORTICES : 16 - 16 - 16

- Extracaps.cort.tiss.: 2 - 2 - 1

- Hypertrophy; diffuse: - - - - 1

Grade 2: - - - - 1

————————————————————————————————————————————————————

ADRENAL MEDULLAS : 16 - 16 - 16

————————————————————————————————————————————————————

SKELETAL MUSCLE : 16 - 16 - 16

- Infl. cell foci : - - 1 - -

Grade 1: - - 1 - -

————————————————————————————————————————————————————

TONGUE : 16 - 16 - 16

————————————————————————————————————————————————————

SCIATIC NERVE : 16 - 16 - 16

————————————————————————————————————————————————————

SKIN/SUBCUTIS : 16 - 16 - 16

- Epidermoid cyst : - - - - 1

————————————————————————————————————————————————————

MAMMARY GLAND AREA : 16 - 16 - 16

————————————————————————————————————————————————————

EYES : 16 - 16 - 16

- Atrophy; retina : - - 2 - 2

Grade 1: - - 1 - 2

Grade 2: - - 1 - -

————————————————————————————————————————————————————

————————————————————————————————————————————————————

HARDERIAN GLANDS : 16 - 16 - 16

- Infiltrate; lymphoid: 1 - 2 - 1

Grade 1: 1 - 2 - 1

- Inflamm./necrosis : 1 - 2 - 3

Grade 1: 1 - 1 - 1

Grade 2: - - 1 - 1

Grade 3: - - - - 1

- Pigment; brown : - - - - 1

Grade 2: - - - - 1

————————————————————————————————————————————————————

LACRIMAL GLANDS : 16 - 16 - 16

- Metaplasia/Harderian: 2 - - - 3

Grade 1: 2 - - - 3

Grade 2: - - - - -

————————————————————————————————————————————————————

BRAIN : 16 - 16 - 16

————————————————————————————————————————————————————

UTERUS : 16 - 16 - 16

- Dilated lumen : 6 - 5 - 8

Grade 1: 1 - 3 - 3

Grade 2: 5 - 2 - 5

————————————————————————————————————————————————————

CERVIX : 16 - 16 - 16

————————————————————————————————————————————————————

VAGINA : 16 - 16 - 16

- Diestrus : 6 - 8 - 8

- Proestrus : 4 - 5 - 3

- Estrus : 4 - 3 - 5

- Metestrus : 2 - - - -

————————————————————————————————————————————————————

PITUITARY GLAND : 16 - 16 - 16

- Cyst/Pars Anterior : 1 - - - -

————————————————————————————————————————————————————

SPINAL CORD : 16 - 16 - 16

————————————————————————————————————————————————————

BONE, STERNUM : 16 - 16 - 16

————————————————————————————————————————————————————

BONE MARROW, STERNUM : 16 - 16 - 16

- Fibroplasia : - - - - 1

Grade 1: - - - - 1

————————————————————————————————————————————————————

FEMUR/JOINT : 16 - 16 - 16

————————————————————————————————————————————————————

BODY CAVITIES : 1 - 1 - -

- Fat necrosis : 1 - 1 - -

Grade 2: 1 - 1 - -

————————————————————————————————————————————————————

BONE : - - - - 1

- Fracture : - - - - 1

————————————————————————————————————————————————————

ABDOMINAL CAVITY : 1 - 1 - -

————————————————————————————————————————————————————

Infl., inflammatory; PP, periportal; G.A.L.T., gut associated lymphoid tissue; incr., increased cellularity; acin.cel.foc, acinar cell, focal; mononuc, mononuclear cell; extracaps.cort.tiss., extracapsular cortical tissue; inflamm., inflammation

**Table 8:** Necropsy findings in male Wistar Han RCC rats in the 90-day feeding trial with GM maize NK603 at an inclusion rate of 11, 33 and 50% in the diet

| **Group** | **Isogenic maize**  **(% of diet)** | **NK603 only**  **(% of diet)** | **NK603 +**  **Roundup**  **(% of diet)** | **No. of**  **Males** | **No. of**  **Females** |
| --- | --- | --- | --- | --- | --- |
| **1** | 33 | 0 | 0 | 16 | 16 |
| **2** | 0 | 33 | 0 | 16 | 16 |
| **3** | 0 | 0 | 33 | 16 | 16 |
| **4** | 50 | 0 | 0 | 16 | 16 |
| **5** | 39 | 11 | 0 | 16 | 16 |
| **6** | 0 | 50 | 0 | 16 | 16 |
| **7** | 39 | 0 | 11 | 16 | 16 |
| **8** | 0 | 0 | 50 | 16 | 16 |

—————————————————————————————————————————————————————————————————————————

DOSE GROUP: 1 2 3 4 5 6 7 8

—————————————————————————————————————————————————————————————————————————

MANDIBULAR LYMPH NODE

— Discoloration, red : 2 1 1 2 1 1 3 2

- Enlargement : - - - - - - - 1

—————————————————————————————————————————————————————————————————————————

KIDNEY

- Discoloration, white: - - - - 1 1 1 1

- Discoloration, red : 1 1 2 1 1 - 2 4

- Defect : - 3 - - - 1 - -

- Pelvis cystic : - - 1 - - - - -

—————————————————————————————————————————————————————————————————————————

LUNG

- Discoloration, white: 2 2 - 1 2 1 - 2

—————————————————————————————————————————————————————————————————————————

THYMUS

- Discoloration, red : 1 1 1 - - - 1 -

—————————————————————————————————————————————————————————————————————————

DUODENUM

- Discoloration, red : - - 2 2 1 1 - -

—————————————————————————————————————————————————————————————————————————

JEJUNUM

- Discoloration, red : - - 2 1 1 1 2 -

—————————————————————————————————————————————————————————————————————————

ILEUM

- Discoloration, red : - - - - - - 1 -

—————————————————————————————————————————————————————————————————————————

URINARY BLADDER

- Discoloration, red : - 1 - - - - - -

—————————————————————————————————————————————————————————————————————————

CECUM

- Discoloration, red : - 1 - - - - 1 -

—————————————————————————————————————————————————————————————————————————

STOMACH

- Discoloration, red : - - - 1 - - 1 -

—————————————————————————————————————————————————————————————————————————

LIVER

- Discoloration : - - - 1 - 1 2 -

—————————————————————————————————————————————————————————————————————————

SPLEEN

- Discoloration, white: - - - 1 - 1 2 -

—————————————————————————————————————————————————————————————————————————

MESENT. LYMPH NODE

- Discoloration, red : - 1 - - - - - -

—————————————————————————————————————————————————————————————————————————

—————————————————————————————————————————————————————————————————————————

TESTES

- Reduction in size : - - - 1 - - - -

—————————————————————————————————————————————————————————————————————————

EPIDIDYMIDES

- Reduction in size : - - - 1 - - - -

—————————————————————————————————————————————————————————————————————————

PITUITARY

- Discoloration, white: - - - - 1 - - -

—————————————————————————————————————————————————————————————————————————

SKIN

- Alopecia : - - - - - - 1 -

- Nodule : - - - - - - - 1

—————————————————————————————————————————————————————————————————————————

**Table 9:** Necropsy findings in female Wistar Han RCC rats in the 90-day feeding trial with GM maize NK603 at an inclusion rate of 11, 33 and 50% in the diet

| **Group** | **Isogenic maize**  **(% of diet)** | **NK603 only**  **(% of diet)** | **NK603 +**  **Roundup**  **(% of diet)** | **No. of**  **Males** | **No. of**  **Females** |
| --- | --- | --- | --- | --- | --- |
| **1** | 33 | 0 | 0 | 16 | 16 |
| **2** | 0 | 33 | 0 | 16 | 16 |
| **3** | 0 | 0 | 33 | 16 | 16 |
| **4** | 50 | 0 | 0 | 16 | 16 |
| **5** | 39 | 11 | 0 | 16 | 16 |
| **6** | 0 | 50 | 0 | 16 | 16 |
| **7** | 39 | 0 | 11 | 16 | 16 |
| **8** | 0 | 0 | 50 | 16 | 16 |

—————————————————————————————————————————————————————————————————————————

DOSE GROUP: 1 2 3 4 5 6 7 8

—————————————————————————————————————————————————————————————————————————

MANDIBULAR LYMPH NODE

— Discoloration, red : 2 1 1 2 2 0 0 1

—————————————————————————————————————————————————————————————————————————

KIDNEY

- Discoloration, white: - - - - - - 1 -

- Discoloration, red : 1 - - - - - - -

- Discoloration,yellow: - - - - 1 - - -

- Defect : - - - - - 2 1 1

—————————————————————————————————————————————————————————————————————————

LUNG

- Discoloration, white: 2 4 2 4 2 2 2 3

—————————————————————————————————————————————————————————————————————————

THYMUS

- Discoloration, red : - - - 1 - 1 - -

—————————————————————————————————————————————————————————————————————————

JEJUNUM

- Discoloration, red : - - - 1 - - - -

—————————————————————————————————————————————————————————————————————————

ABDOMINAL CAVITY

- Nodule : 1 - 1 - - - - -

—————————————————————————————————————————————————————————————————————————

CECUM

- Discoloration, red : 1 - - - - - - -

—————————————————————————————————————————————————————————————————————————

LIVER

- Discoloration : - - - 1 - - - -

—————————————————————————————————————————————————————————————————————————

SPLEEN

- Discoloration, white: - - 1 1 - - - -

—————————————————————————————————————————————————————————————————————————

OVARIES

- Nodule : - 1 - - - - - -

- Cystic fluid : - - - - - - - 1

—————————————————————————————————————————————————————————————————————————

UTERUS

- Enlarged, cystic : - - - - 1 - - -

—————————————————————————————————————————————————————————————————————————

**Table 10:** Microscopic findings in male Wistar Han RCC rats in the 90-day feeding trial with GM maize NK603 at an inclusion rate of 11, 33 and 50% in the diet

| **Group** | **Isogenic maize**  **(% of diet)** | **NK603 only**  **(% of diet)** | **NK603 +**  **Roundup**  **(% of diet)** | **No. of**  **Males** | **No. of**  **Females** |
| --- | --- | --- | --- | --- | --- |
| **1** | 33 | 0 | 0 | 16 | 16 |
| **2** | 0 | 33 | 0 | 16 | 16 |
| **3** | 0 | 0 | 33 | 16 | 16 |
| **4** | 50 | 0 | 0 | 16 | 16 |
| **5** | 39 | 11 | 0 | 16 | 16 |
| **6** | 0 | 50 | 0 | 16 | 16 |
| **7** | 39 | 0 | 11 | 16 | 16 |
| **8** | 0 | 0 | 50 | 16 | 16 |

—————————————————————————————————————————————————————————————————————————

DOSE GROUP: 1 2 3 4 5 6 7 8

—————————————————————————————————————————————————————————————————————————

HEART : 16 — — 16 — 16 — 16

— Cardiomyopathy : 3 — — 5 — 4 — 2

Grade 1: 2 — — 5 — 4 — 2

Grade 2: 1 — — — — — — —

—————————————————————————————————————————————————————————————————————————

AORTA : 16 — — 16 — 15 — 15

—————————————————————————————————————————————————————————————————————————

ESOPHAGUS : 16 — — 15 — 16 — 16

—————————————————————————————————————————————————————————————————————————

TRACHEA : 16 — — 16 — 16 — 16

—————————————————————————————————————————————————————————————————————————

LIVER : 16 — — 16 — 16 1 16

— Infl. cell foci : 15 — — 13 — 15 — 13

Grade 1: 14 — — 12 — 14 — 12

Grade 2: 1 — — 1 — 1 — 1

— Vacuolation; PP : 2 — — — — 1 — 3

Grade 1: 2 — — — — 1 — 2

Grade 2: — — — — — — — 1

— Vacuolation; CL : — — — 1 — — — —

Grade 1: — — — 1 — — — —

— Necrosis; focal : — — — 1 — — — —

Grade 2: — — — 1 — — — —

— Hyperpl; bile duct : — — — 1 — — — —

Grade 1: — — — 1 — — — —

— Necrosis;single cell: 1 — — — — — — —

Grade 1: 1 — — — — — — —

— Pigment; hepatocytes: — — — — — — — 1

Grade 1: — — — — — — — 1

Grade 2: — — — — — — — —

— Pigment; macrophages: — — — — — — — 1

Grade 1: — — — — — — — 1

—————————————————————————————————————————————————————————————————————————

SPLEEN : 16 — — 16 — 16 2 16

— Hematopoiesis : 11 — — 8 — 10 1 11

Grade 1: 10 — — 8 — 10 1 8

Grade 2: 1 — — — — — — 3

— Hemosiderin : 16 — — 16 — 16 2 16

Grade 1: 5 — — 11 — 13 2 9

Grade 2: 11 — — 5 — 3 — 7

Grade 3: — — — — — — — —

—————————————————————————————————————————————————————————————————————————

—————————————————————————————————————————————————————————————————————————

MESENT. LYMPH NODE : 16 1 — 16 — 16 — 16

— Ceroid : 1 — — 2 — — — 2

Grade 1: 1 — — 2 — — — 2

— Dilation; sinusoid : 1 — — 1 — — — 1

Grade 1: 1 — — — — — — —

Grade 2: — — — 1 — — — 1

— Erythrocytosis : 1 — — — — — — 1

Grade 1: 1 — — — — — — 1

—————————————————————————————————————————————————————————————————————————

KIDNEYS : 16 4 3 16 1 16 3 16

— Cyst; cortex : — — — — 1 — — —

Grade 1: — — — — 1 — — —

— Hemorrhage; agonal : 1 2 1 3 1 — 2 4

— Dilatation; pelvis : — 1 2 2 — — — —

Grade 1: — 1 1 — — — — —

Grade 2: — — — 2 — — — —

Grade 3: — — 1 — — — — —

— Tubular basophilia : 2 — — 3 — 1 — 2

Grade 1: 2 — — 3 — 1 — 2

— Tubular casts : — — — — — 1 — —

Grade 1: — — — — — 1 — —

—————————————————————————————————————————————————————————————————————————

URINARY BLADDER : 16 1 — 16 — 16 — 16

—————————————————————————————————————————————————————————————————————————

DUODENUM : 16 — 2 16 1 16 — 16

— Hemorrhage : — — 2 2 1 — — —

Grade 1: — — 1 2 — — — —

Grade 2: — — 1 — 1 — — —

—————————————————————————————————————————————————————————————————————————

JEJUNUM : 16 — 2 16 1 16 2 16

— Hemorrhage : — — 2 1 1 1 1 —

Grade 1: — — 2 1 — 1 — —

Grade 2: — — — — 1 — 1 —

— Congestion : — — — — — — 1 —

Grade 1: — — — — — — 1 —

—————————————————————————————————————————————————————————————————————————

ILEUM : 16 — — 16 — 16 1 16

—————————————————————————————————————————————————————————————————————————

G.A.L.T. : 8 — — 9 — 8 — 10

— Lipid : — — — 1 — 2 — —

Grade 1: — — — 1 — 2 — —

Grade 2: — — — — — — — —

— Mineralisation : — — — — — — — 1

Grade 1: — — — — — — — 1

—————————————————————————————————————————————————————————————————————————

CECUM : 16 — — 16 — 16 1 16

— Hemorrhage : 1 — — — — — 1 —

Grade 1: — — — — — — 1 —

Grade 2: 1 — — — — — — —

—————————————————————————————————————————————————————————————————————————

COLON : 16 — — 16 — 16 — 16

—————————————————————————————————————————————————————————————————————————

RECTUM : 16 — — 16 — 16 — 16

—————————————————————————————————————————————————————————————————————————

STOMACH : — — — 1 — — 1 —

—————————————————————————————————————————————————————————————————————————

FORESTOMACH : 16 — — 16 — 16 — 16

—————————————————————————————————————————————————————————————————————————

—————————————————————————————————————————————————————————————————————————

GLANDULAR STOMACH : 16 — — 16 — 16 1 16

— Dilated glands : 4 — — 2 — 1 — 2

Grade 1: 4 — — 2 — 1 — 2

— Inflammation; submuc: 2 — — 2 — — — —

Grade 1: 1 — — 2 — — — —

Grade 2: 1 — — — — — — —

— Ectopic squamous ep : 2 — — 1 — 1 — 2

Grade 1: 2 — — 1 — 1 — 2

— Degeneration; glands: — — — — — 1 — —

Grade 1: — — — — — 1 — —

— Hemorrhage : 1 — — — — — — —

Grade 1: 1 — — — — — — —

—————————————————————————————————————————————————————————————————————————

LUNG : 16 2 — 16 2 16 — 16

— Alveolar macrophages: 4 1 — 7 1 5 — 6

Grade 1: 2 — — 4 1 3 — 6

Grade 2: 2 1 — 3 — 2 — —

Grade 3: — — — — — — — —

— Agonal hemorrhage : — 1 — 4 — 2 — —

Grade 1: — 1 — 3 — 2 — —

Grade 2: — — — 1 — — — —

— Alveolar bone : — — — 1 — — — 1

Grade 1: — — — 1 — — — 1

—————————————————————————————————————————————————————————————————————————

THYMUS : 16 1 1 16 — 16 1 16

— Agonal hemorrhage : 2 1 1 3 — 1 1 3

Grade 1: 1 1 — 3 — 1 — 3

Grade 2: — — 1 — — — 1 —

Grade 3: 1 — — — — — — —

—————————————————————————————————————————————————————————————————————————

TESTES : 16 — — 16 — 16 — 16

— Sertoli C. Vacuolat.: — — — 2 — 1 — 1

Grade 1: — — — 2 — 1 — 1

— Tubular atrophy : — — — 1 — — — —

Grade 4: — — — 1 — — — —

—————————————————————————————————————————————————————————————————————————

EPIDIDYMIDES : 16 — — 16 — 16 — 16

— Decrease spermatozoa: — — — 1 — — — —

Grade 4: — — — 1 — — — —

— Cellular debris : — — — 1 — — — —

Grade 1: — — — 1 — — — —

—————————————————————————————————————————————————————————————————————————

SUBLINGUAL GLANDS : 16 — — 16 — 16 — 16

—————————————————————————————————————————————————————————————————————————

MANDIBULAR GLANDS : 16 — — 16 — 16 — 16

—————————————————————————————————————————————————————————————————————————

MANDIB.LYMPH NODES : 15 2 1 15 1 16 3 15

— Erythrocytosis : 3 1 — 4 1 1 3 1

Grade 1: 3 — — 3 1 1 2 1

Grade 2: — 1 — 1 — — 1 —

Grade 3: — — — — — — — —

— Hemosiderin : — — — 1 — — 2 —

Grade 1: — — — 1 — — 2 —

— Dilatation; sinusoid: — — — — — — — 1

Grade 1: — — — — — — — 1

—————————————————————————————————————————————————————————————————————————

—————————————————————————————————————————————————————————————————————————

PANCREAS : 16 — — 16 — 16 — 16

— Apoptosis : 2 — — 3 — 3 — —

Grade 1: 2 — — 3 — 3 — —

— Atrophy;acin.cel.foc: 5 — — 4 — 4 — 4

Grade 1: 4 — — 3 — 3 — 3

Grade 2: 1 — — 1 — 1 — —

Grade 3: — — — — — — — 1

— Hyperpl;Islet cell : 1 — — — — — — —

Grade 1: 1 — — — — — — —

—————————————————————————————————————————————————————————————————————————

THYROID GLANDS : 16 — — 16 — 16 — 16

—————————————————————————————————————————————————————————————————————————

PARATHYROID GLANDS : 14 — — 16 — 13 — 14

—————————————————————————————————————————————————————————————————————————

ADRENAL CORTICES : 16 — — 16 — 16 — 16

— Extracaps.cort.tiss.: — — — 1 — — — 2

— Vacuolation : — — — — — — — 1

Grade 1: — — — — — — — 1

—————————————————————————————————————————————————————————————————————————

ADRENAL MEDULLAS : 16 — — 16 — 16 — 16

—————————————————————————————————————————————————————————————————————————

SKELETAL MUSCLE : 16 — — 16 — 16 — 16

— Infl. cell foci : — — — 1 — — — 1

Grade 1: — — — 1 — — — 1

—————————————————————————————————————————————————————————————————————————

TONGUE : 16 — — 16 — 16 — 16

— Atrophy; salivary gl: — — — — — — — 1

Grade 1: — — — — — — — 1

—————————————————————————————————————————————————————————————————————————

SCIATIC NERVE : 16 — — 16 — 16 — 16

—————————————————————————————————————————————————————————————————————————

SKIN/SUBCUTIS : 16 — — 16 — 16 1 16

— Fat necrosis : — — — — — — — 1

Grade 2: — — — — — — — 1

—————————————————————————————————————————————————————————————————————————

MAMMARY GLAND AREA : 16 — — 16 — 16 — 16

—————————————————————————————————————————————————————————————————————————

EYES : 16 — — 16 — 16 — 16

— Atrophy; retina : — — — 1 — — — —

Grade 1: — — — 1 — — — —

—————————————————————————————————————————————————————————————————————————

HARDERIAN GLANDS : 16 — — 16 — 16 — 16

— Infiltrate; lymphoid: — — — — — 1 — 1

Grade 1: — — — — — 1 — 1

Grade 2: — — — — — — — —

— Pigment; brown : 5 — — 8 — 1 — 10

Grade 1: 5 — — 8 — 1 — 10

—————————————————————————————————————————————————————————————————————————

LACRIMAL GLANDS : 16 — — 16 — 16 — 16

— Degeneration/inflamm: — — — 2 — — — —

Grade 1: — — — 2 — — — —

— Metaplasia;Harderian: 3 — — 6 — 5 — 4

Grade 1: 3 — — 5 — 5 — 4

Grade 2: — — — 1 — — — —

— Infiltrate; lymphoid: 3 — — 1 — 3 — 2

Grade 1: 3 — — 1 — 3 — 2

Grade 2: — — — — — — — —

—————————————————————————————————————————————————————————————————————————

BRAIN : 16 — — 16 — 16 — 16

—————————————————————————————————————————————————————————————————————————

—————————————————————————————————————————————————————————————————————————

PROSTATE GLAND : 16 — — 16 — 16 — 16

— Infiltrate; lymphoid: 4 — — 3 — 3 — 5

Grade 1: 2 — — 2 — 1 — 4

Grade 2: 2 — — 1 — 2 — 1

—————————————————————————————————————————————————————————————————————————

COAGULATING GLANDS : 16 — — 16 — 16 — 16

—————————————————————————————————————————————————————————————————————————

SEMINAL VESICLES : 16 — — 16 — 16 — 16

—————————————————————————————————————————————————————————————————————————

PITUITARY GLAND : 16 — — 16 1 16 — 16

— Cyst; Pars Anterior : — — — 3 — 2 — 2

— Angiectasis : — — — 1 — — — —

Grade 1: — — — 1 — — — —

—————————————————————————————————————————————————————————————————————————

SPINAL CORD : 16 — — 16 — 16 — 16

—————————————————————————————————————————————————————————————————————————

BONE, STERNUM : 16 — — 16 — 16 — 16

— Development anomaly : — — — — — — — 1

—————————————————————————————————————————————————————————————————————————

BONE MARROW, STERNUM : 16 — — 16 — 16 — 16

—————————————————————————————————————————————————————————————————————————

FEMUR/JOINT : 16 — — 16 — 16 — 16

—————————————————————————————————————————————————————————————————————————

MEDIAST. LYMPH NODE : — — — 2 — — — —

— Hemorrhage : — — — 2 — — — —

Grade 2: — — — 2 — — — —

— Pigment: hemosiderin: — — — 1 — — — —

Grade 1: — — — — — — — —

Grade 2: — — — 1 — — — —

—————————————————————————————————————————————————————————————————————————

ABDOMINAL CAVITY : — — — — — — — 2

— Infilt; mixed cells : — — — — — — — 2

Grade 1: — — — — — — — 1

Grade 2: — — — — — — — 1

—————————————————————————————————————————————————————————————————————————

Infl., inflammatory; hyperpl, hyperplasia; G.A.L.T., gut associated lymphoid tissue; ectopic squamous ep, ectopic squamous epithelium; Sertoli C. Vacuolat., Sertoli cell vacuolation; mandib., mandibular; acin.cel.foc, acinar cell, focal; extracaps.cort.tiss., extracapsular cortical tissue; salivary gl, salivary gland; inflamm., inflammation; infilt, infiltrate

**Table 11:** Microscopic findings in female Wistar Han RCC rats in the 90-day feeding trial with GM maize NK603 at an inclusion rate of 11, 33 and 50% in the diet

| **Group** | **Isogenic maize**  **(% of diet)** | **NK603 only**  **(% of diet)** | **NK603 +**  **Roundup**  **(% of diet)** | **No. of**  **Males** | **No. of**  **Females** |
| --- | --- | --- | --- | --- | --- |
| **1** | 33 | 0 | 0 | 16 | 16 |
| **2** | 0 | 33 | 0 | 16 | 16 |
| **3** | 0 | 0 | 33 | 16 | 16 |
| **4** | 50 | 0 | 0 | 16 | 16 |
| **5** | 39 | 11 | 0 | 16 | 16 |
| **6** | 0 | 50 | 0 | 16 | 16 |
| **7** | 39 | 0 | 11 | 16 | 16 |
| **8** | 0 | 0 | 50 | 16 | 16 |

—————————————————————————————————————————————————————————————————————————

DOSE GROUP: 1 2 3 4 5 6 7 8

—————————————————————————————————————————————————————————————————————————

HEART : 16 — — 16 — 16 — 16

— Cardiomyopathy : 1 — — — — 1 — —

Grade 1: 1 — — — — 1 — —

Grade 2: — — — — — — — —

—————————————————————————————————————————————————————————————————————————

AORTA : 16 — — 16 — 16 — 16

—————————————————————————————————————————————————————————————————————————

ESOPHAGUS : 16 — — 16 — 16 — 16

—————————————————————————————————————————————————————————————————————————

TRACHEA : 16 — — 16 — 16 — 16

—————————————————————————————————————————————————————————————————————————

LIVER : 16 — — 16 — 16 — 16

— Infl. cell foci : 11 — — 10 — 6 — 7

Grade 1: 11 — — 10 — 6 — 7

Grade 2: — — — — — — — —

— Vacuolation; PP : 5 — — 1 — 3 — 4

Grade 1: 5 — — 1 — 3 — 4

Grade 2: — — — — — — — —

— Pigment; hepatocytes: 1 — — — — — — 1

Grade 1: — — — — — — — 1

Grade 2: 1 — — — — — — —

—————————————————————————————————————————————————————————————————————————

SPLEEN : 16 — 1 16 — 16 — 16

— Hematopoiesis : 12 — — 10 — 13 — 8

Grade 1: 7 — — 3 — 7 — 5

Grade 2: 5 — — 7 — 6 — 3

— Hemosiderin : 16 — 1 16 — 16 — 16

Grade 1: 3 — 1 5 — 3 — 2

Grade 2: 12 — — 10 — 13 — 13

Grade 3: 1 — — 1 — — — 1

—————————————————————————————————————————————————————————————————————————

MESENT. LYMPH NODE : 16 — — 16 — 16 — 16

— Dilation; sinusoid : 2 — — — — 1 — —

Grade 1: 2 — — — — 1 — —

Grade 2: — — — — — — — —

— Erythrocytosis : — — — 1 — 1 — 1

Grade 1: — — — 1 — 1 — 1

—————————————————————————————————————————————————————————————————————————

—————————————————————————————————————————————————————————————————————————

KIDNEYS : 16 — — 15 1 16 2 16

— Dilatation; pelvis : — — — — — 1 1 1

Grade 1: — — — — — 1 1 —

Grade 2: — — — — — — — —

Grade 3: — — — — — — — 1

— Infiltrate; lymphoid: — — — 1 — — — —

Grade 1: — — — 1 — — — —

— Tubular basophilia : — — — 1 — — — —

Grade 1: — — — 1 — — — —

— Tubular casts : 1 — — — — — — —

Grade 1: 1 — — — — — — —

— Scar; cortex : — — — — 1 1 — —

Grade 2: — — — — — 1 — —

Grade 3: — — — — 1 — — —

—————————————————————————————————————————————————————————————————————————

URINARY BLADDER : 16 — — 15 — 16 — 16

—————————————————————————————————————————————————————————————————————————

DUODENUM : 16 — — 16 — 16 — 16

— Inflammation; submuc: — — — 1 — — — —

Grade 1: — — — 1 — — — —

—————————————————————————————————————————————————————————————————————————

JEJUNUM : 16 — — 16 — 16 — 16

— Congestion : — — — 1 — — — —

Grade 1: — — — 1 — — — —

—————————————————————————————————————————————————————————————————————————

ILEUM : 16 — — 16 — 16 — 15

—————————————————————————————————————————————————————————————————————————

G.A.L.T. : 6 — — 8 — 6 — 12

— Lipid : 1 — — — — 2 — —

Grade 1: — — — — — 1 — —

Grade 2: 1 — — — — 1 — —

— Incr;germinal centre: — — — 1 — — — —

Grade 2: — — — 1 — — — —

—————————————————————————————————————————————————————————————————————————

CECUM : 16 — — 16 — 16 — 16

— Hemorrhage : 1 — — — — — — —

Grade 1: 1 — — — — — — —

Grade 2: — — — — — — — —

—————————————————————————————————————————————————————————————————————————

COLON : 16 — — 16 — 16 — 16

—————————————————————————————————————————————————————————————————————————

RECTUM : 16 — — 16 — 16 — 16

—————————————————————————————————————————————————————————————————————————

FORESTOMACH : 15 — — 16 — 16 — 16

—————————————————————————————————————————————————————————————————————————

GLANDULAR STOMACH : 16 — — 16 — 16 — 16

— Dilated glands : — — — 1 — 1 — 2

Grade 1: — — — 1 — 1 — 2

— Ectopic squamous ep : 1 — — — — 1 — —

Grade 1: 1 — — — — 1 — —

— Degeneration; glands: 5 — — 2 — — — 1

Grade 1: 5 — — 2 — — — 1

— Hemorrhage : 1 — — — — — — —

Grade 1: 1 — — — — — — —

—————————————————————————————————————————————————————————————————————————

—————————————————————————————————————————————————————————————————————————

LUNG : 16 4 2 16 2 16 2 16

— Alveolar macrophages: 5 4 2 10 2 7 2 8

Grade 1: 4 2 — 2 2 5 — 5

Grade 2: 1 2 2 8 — 2 1 3

Grade 3: — — — — — — 1 —

— Agonal hemorrhage : 1 — — — — — — 1

Grade 1: 1 — — — — — — 1

Grade 2: — — — — — — — —

— Alveolar bone : 1 — — — — — — —

Grade 1: 1 — — — — — — —

—————————————————————————————————————————————————————————————————————————

THYMUS : 16 — — 16 — 16 — 16

— Agonal hemorrhage : 2 — — 1 — 1 — 1

Grade 1: 2 — — 1 — 1 — 1

Grade 2: — — — — — — — —

Grade 3: — — — — — — — —

—————————————————————————————————————————————————————————————————————————

OVARIES : 16 1 — 16 — 16 — 16

— Corpora lutea : 15 — — 16 — 16 — 16

— Cyst; luteal : 1 — — — — — — —

— Cyst; follicular : 1 — — — — — — —

— YOLK SACK CARCINOMA : — 1 — — — — — —

—————————————————————————————————————————————————————————————————————————

SUBLINGUAL GLANDS : 16 — — 15 — 15 — 15

—————————————————————————————————————————————————————————————————————————

MANDIBULAR GLANDS : 16 — — 16 — 16 — 16

—————————————————————————————————————————————————————————————————————————

MANDIB.LYMPH NODES : 16 1 1 14 2 16 — 16

— Erythrocytosis : 3 1 1 3 2 2 — 3

Grade 1: 3 1 — 2 2 2 — 2

Grade 2: — — — 1 — — — 1

Grade 3: — — 1 — — — — —

— Hemosiderin : — — — — — 1 — —

Grade 1: — — — — — 1 — —

—————————————————————————————————————————————————————————————————————————

PANCREAS : 16 — — 16 — 16 — 16

— Atrophy;acin.cel.foc: 3 — — 2 — 3 — 2

Grade 1: 2 — — 2 — 3 — 2

Grade 2: 1 — — — — — — —

Grade 3: — — — — — — — —

—————————————————————————————————————————————————————————————————————————

THYROID GLANDS : 16 — — 16 — 16 — 16

— C—cell hyperpl; diff: 1 — — — — — — 1

Grade 1: 1 — — — — — — 1

—————————————————————————————————————————————————————————————————————————

PARATHYROID GLANDS : 16 — — 16 — 16 — 14

—————————————————————————————————————————————————————————————————————————

ADRENAL CORTICES : 16 — — 16 — 16 — 16

— Extracaps.cort.tiss.: — — — 2 — 4 — 4

— Congestion : 2 — — 1 — 1 — —

Grade 1: 2 — — 1 — 1 — —

—————————————————————————————————————————————————————————————————————————

ADRENAL MEDULLAS : 16 — — 16 — 16 — 15

—————————————————————————————————————————————————————————————————————————

SKELETAL MUSCLE : 16 — — 16 — 16 — 16

— Infl. cell foci : 1 — — 1 — 1 — 1

Grade 1: 1 — — 1 — 1 — 1

—————————————————————————————————————————————————————————————————————————

TONGUE : 16 — — 16 — 16 — 16

—————————————————————————————————————————————————————————————————————————

—————————————————————————————————————————————————————————————————————————

SCIATIC NERVE : 16 — — 16 — 16 — 16

—————————————————————————————————————————————————————————————————————————

SKIN/SUBCUTIS : 16 — — 16 — 16 — 16

— Epidermoid cyst : 1 — — — — — — —

—————————————————————————————————————————————————————————————————————————

MAMMARY GLAND AREA : 16 — — 16 — 16 — 16

—————————————————————————————————————————————————————————————————————————

EYES : 16 — — 16 — 16 — 16

— Atrophy; retina : 5 — — 1 — 3 — 2

Grade 1: 5 — — 1 — 3 — 2

—————————————————————————————————————————————————————————————————————————

HARDERIAN GLANDS : 16 — — 16 — 16 — 16

— Infiltrate; lymphoid: — — — 3 — 2 — 1

Grade 1: — — — 2 — 2 — 1

Grade 2: — — — 1 — — — —

— Inflammat/necrosis : 1 — — 1 — 1 — 2

Grade 1: 1 — — 1 — 1 — 2

— Pigment; brown : 7 — — 8 — 8 — 6

Grade 1: 7 — — 8 — 8 — 6

—————————————————————————————————————————————————————————————————————————

LACRIMAL GLANDS : 16 — — 16 — 16 — 16

— Metaplasia;Harderian: 1 — — — — — — —

Grade 1: 1 — — — — — — —

Grade 2: — — — — — — — —

— Infiltrate; lymphoid: — — — — — — — 2

Grade 1: — — — — — — — 1

Grade 2: — — — — — — — 1

—————————————————————————————————————————————————————————————————————————

BRAIN : 16 — — 16 — 16 — 16

—————————————————————————————————————————————————————————————————————————

UTERUS : 16 — — 16 1 16 — 16

— Dilated lumen : 6 — — 6 1 9 — 7

Grade 1: 3 — — 1 — 6 — 6

Grade 2: 3 — — 5 1 3 — 1

—————————————————————————————————————————————————————————————————————————

CERVIX : 16 — — 16 — 16 — 16

—————————————————————————————————————————————————————————————————————————

VAGINA : 16 — — 16 — 15 — 15

— Diestrus : 6 — — 8 — 7 — 4

— Proestrus : 6 — — 4 — 5 — 5

— Estrus : 3 — — 3 — 2 — 3

— Metestrus : — — — 1 — 1 — 3

—————————————————————————————————————————————————————————————————————————

PITUITARY GLAND : 16 — — 16 — 16 — 16

— Cyst; Pars Anterior : 1 — — 1 — 2 — 1

— Angiectasis : 1 — — — — — — —

Grade 1: 1 — — — — — — —

—————————————————————————————————————————————————————————————————————————

SPINAL CORD : 16 — — 16 — 16 — 16

—————————————————————————————————————————————————————————————————————————

BONE, STERNUM : 16 — — 16 — 16 — 16

—————————————————————————————————————————————————————————————————————————

BONE MARROW, STERNUM : 16 — — 16 — 16 — 16

—————————————————————————————————————————————————————————————————————————

FEMUR/JOINT : 16 — — 16 — 16 — 16

—————————————————————————————————————————————————————————————————————————

—————————————————————————————————————————————————————————————————————————

MEDIAST. LYMPH NODE : — — — 1 — — — —

— Hemorrhage : — — — 1 — — — —

Grade 2: — — — 1 — — — —

— Pigment: hemosiderin: — — — 1 — — — —

Grade 1: — — — 1 — — — —

Grade 2: — — — — — — — —

—————————————————————————————————————————————————————————————————————————

BODY CAVITIES : 1 — 1 — — — — —

— Fat necrosis : 1 — 1 — — — — —

Grade 2: 1 — 1 — — — — —

—————————————————————————————————————————————————————————————————————————

ABDOMINAL CAVITY : 1 — 1 5 — 1 — 1

— Infilt; mixed cells : 1 — — 5 — 1 — 1

Grade 1: 1 — — 5 — 1 — 1

Grade 2: — — — — — — — —

—————————————————————————————————————————————————————————————————————————

Infl., inflammatory; submuc, submucosal; G.A.L.T., gut associated lymphoid tissue; incr., increased cellularity; acin.cel.foc, acinar cell, focal; hyperpl, hyperplasia; diff, diffuse; extracaps.cort.tiss., extracapsular cortical tissue; infilt, infiltrate

**Table 12:** Microscopic findings in male and female Wistar Han RCC rats in the chronic toxicity phase of the combined chronic toxicity/carcinogenicity feeding trial with GM maize NK603 at an inclusion rate of 11 and 33% in the diet

| **Group** | **Isogenic maize**  **(% of diet)** | **NK603 only**  **(% of diet)** | **NK603 +**  **Roundup**  **(% of diet)** | **No. of**  **Males** | **No. of**  **Females** |
| --- | --- | --- | --- | --- | --- |
| **1** | 33 | 0 | 0 | 20 | 20 |
| **2** | 22 | 11 | 0 | 20 | 20 |
| **3** | 0 | 33 | 0 | 20 | 20 |
| **4** | 22 | 0 | 11 | 20 | 20 |
| **5** | 0 | 0 | 33 | 20 | 20 |

—————————————————————————————————————————————————————————————————————————

DOSE GROUP: 1 2 3 4 5

SEX : M F M F M F M F M F

—————————————————————————————————————————————————————————————————————————

GENERAL OBSERVATIONS : 1 — — — 1 — — — — —

— Autolysis : — — — — 1 — — — — —

—————————————————————————————————————————————————————————————————————————

SYSTEMIC NEOPLASMS : — — — — — — 1 — — —

— MALIGNANT LYMPHOMA : — — — — — — 1 — — —

—————————————————————————————————————————————————————————————————————————

HEART : 20 20 1 — 20 20 1 — 20 20

— Cardiomyopathy : 8 4 1 — 12 4 — — 12 4

— Degeneration; valve : 1 — — — — — — — — —

— Hypertrophy; myocard: — — — — 1 — — — — —

—————————————————————————————————————————————————————————————————————————

AORTA : 20 20 1 — 20 20 1 — 20 20

—————————————————————————————————————————————————————————————————————————

ESOPHAGUS : 20 20 1 — 20 20 1 — 20 20

— Hyperkeratosis : — — 1 — — — — — — —

—————————————————————————————————————————————————————————————————————————

TRACHEA : 20 20 1 — 20 20 1 — 20 20

— Blood in lumen : — — — — 1 — — — — —

— Inflammation; wall : — — — — 1 — — — — —

—————————————————————————————————————————————————————————————————————————

LIVER : 20 20 2 1 20 20 1 7 20 20

— Altered focus; clear: 2 — — — — — — — 1 —

— Altered focus; eosin: 4 — — — 2 2 — — 1 —

— Altered focus;basoph: 1 6 1 — 5 10 — — 3 5

— Altered focus;vacuol: 2 — — — 1 — — 1 1 —

— Angiectasis : — 2 — — — — — — — —

— Degen/necrosis; CL : — 1 — — — — — — — —

— Fibrosis; bile duct : — — — — 1 — — — — —

— Hematopoiesis : — 2 — — — 1 — — — —

— Hyperpl; bile duct : — — — — — — — — — 1

— Infl. cell foci : 18 10 — — 15 11 — — 19 10

— Lobar torsion : 1 — — — — — — — — —

— Necrosis; focal : — — — — — 1 — — — —

— Necrosis;single cell: 1 — — — — — — — — —

— Pigment; hepatocytes: — 2 — — — — — — 1 —

— Pigment; macrophages: 1 4 — 1 2 2 — — 1 2

— MALIGNANT LYMPHOMA : — — — — — — 1 — — —

— Tension lipidosis : — — — — — — — 1 1 —

— Vacuolation; CL : 4 — — — 1 — — — 3 —

— Vacuolation; PP : 5 5 1 — 7 1 — — 10 5

— Vacuolation; diffuse: 1 — — — — — — — — —

— Vacuolation; focal : — 1 1 — 4 1 — — 4 —

—————————————————————————————————————————————————————————————————————————

—————————————————————————————————————————————————————————————————————————

SPLEEN : 20 20 2 — 19 20 2 — 20 20

— Hematopoiesis : 19 20 1 — 17 16 — — 18 18

— Hemosiderin : 20 18 1 — 19 20 — — 20 19

— Mineralization : — — — — 1 — — — — —

— MALIGNANT LYMPHOMA : — — — — — — 1 — — —

—————————————————————————————————————————————————————————————————————————

MESENT. LYMPH NODE : 20 20 2 — 20 20 2 1 20 20

— Ceroid : 11 19 — — 11 19 — — 10 19

— Dilation; sinusoid : 1 — — — — 1 — — 2 2

— Erythrocytosis : 6 1 1 — 5 3 — — 4 4

— Pigment; hemosiderin: — — — — — — — — — 1

—————————————————————————————————————————————————————————————————————————

KIDNEYS : 20 20 4 1 20 20 7 2 20 20

— Chron. Prog. Nephrop: 14 7 3 — 12 3 — 1 7 9

— Dilatation; pelvis : — — — — 1 — — 1 — 1

— Hyperplasia; urothel: — 1 — — — — — — — —

— Infiltrate; lymphoid: 1 — — — — — — — — —

— Inflammation; pelvis: — 1 — — — — — — 1 1

— Mineral; pelvis : — 1 — — — — — — — —

— Pigment; cortex : — 1 — — — — — — — —

—————————————————————————————————————————————————————————————————————————

URINARY BLADDER : 20 20 2 — 20 19 1 — 20 20

— Seminal plug : — — 1 — 1 — — — — —

—————————————————————————————————————————————————————————————————————————

DUODENUM : 20 20 2 10 20 20 2 6 20 20

— Hemorrhage : — 10 1 7 — 9 — 4 2 7

—————————————————————————————————————————————————————————————————————————

JEJUNUM : 20 20 7 6 20 20 9 12 20 20

— Congestion : — — — 3 — — — — — —

— Hemorrhage : 3 5 5 1 2 5 3 8 7 5

—————————————————————————————————————————————————————————————————————————

ILEUM : 20 20 1 — 20 20 1 — 20 20

— Hemorrhage : 1 1 — — — 1 — — — 1

—————————————————————————————————————————————————————————————————————————

G.A.L.T. : 17 13 1 — 14 9 1 — 15 11

— Erythrocytosis : — 1 — — — — — — — —

— Lipid : 2 1 — — 1 1 — — 2 —

—————————————————————————————————————————————————————————————————————————

CECUM : 20 20 2 — 19 20 4 1 20 20

— Hemorrhage : — — — — — — — — — 1

—————————————————————————————————————————————————————————————————————————

COLON : 20 20 1 — 19 20 2 — 20 20

— Hemorrhage : 1 — — — — — — — — —

—————————————————————————————————————————————————————————————————————————

RECTUM : 20 20 1 — 19 20 1 — 20 20

—————————————————————————————————————————————————————————————————————————

STOMACH : — 1 1 — — — — — — —

—————————————————————————————————————————————————————————————————————————

FORESTOMACH : 20 20 1 — 19 20 1 — 20 20

— Hyperpl;epithel;foc : — — — — — — 1 — — —

— Ulceration : 1 — — — — — — — — —

—————————————————————————————————————————————————————————————————————————

GLANDULAR STOMACH : 20 20 2 — 19 20 2 — 20 20

— Degeneration; glands: — — — — 2 — — — — —

— Dilated glands : 10 1 — — 4 2 — — 6 3

— Ectopic squamous ep : — — — — — — — — — 1

— Hemorrhage : — 1 — — 1 — 1 — 1 —

— Inflammation; submuc: — 1 — — — 1 — — — —

—————————————————————————————————————————————————————————————————————————

—————————————————————————————————————————————————————————————————————————

LUNG : 20 20 7 10 20 20 3 7 20 20

— Agonal hemorrhage : 2 — — — 1 1 — — 1 —

— Alveolar macrophages: 6 16 5 4 3 13 1 4 9 15

— Congestion : 1 — — — — — — — — —

— Hyperpl; foc; alv/br: 1 — — — — — — — — —

— Hyperpl; foc; alveol: — — — — — — — — 1 —

— Metastasis : — — — — — — — — — 1

— Osseous metaplasia : — — — — 1 — — — 1 —

—————————————————————————————————————————————————————————————————————————

THYMUS : 20 20 5 20 19 19 6 18 19 20

— Agonal hemorrhage : 4 1 1 — 7 3 1 — 3 2

— Atrophy; lymphoid : — — — 2 2 — 1 — — 2

—————————————————————————————————————————————————————————————————————————

TESTES : 20 — 2 — 20 — 2 — 20 —

— Atrophy; tubule : 1 — 2 — — — 1 — 3 —

— Hyperpl;mesoth;focal: — — — — 1 — — — — —

—————————————————————————————————————————————————————————————————————————

EPIDIDYMIDES : 20 — 1 — 20 — 1 — 20 —

— Decrease spermatozoa: — — 1 — — — — — — —

— Hyperpl;mesoth;focal: — — — — 1 — — — — —

—————————————————————————————————————————————————————————————————————————

OVARIES : — 20 — 2 — 20 — 1 — 20

— Corpora lutea : — 9 — — — 12 — — — 11

— Cyst; NOS : — — — — — — — — — 1

— Cyst; follicular : — 10 — — — 8 — — — 9

— Cyst; luteal : — — — — — 2 — 1 — —

— Hyperpl;SCS; diffuse: — 11 — — — 7 — — — 11

—————————————————————————————————————————————————————————————————————————

MANDIB.LYMPH NODES : 20 19 12 5 20 20 12 6 20 20

— Dilatation; sinusoid: 1 — — — — — — — — —

— Erythrocytosis : 17 5 7 5 15 4 11 5 14 8

— Infilt; plasma cell : — 1 2 — 1 2 — — — 2

— Pigment; hemosiderin: — — — — — 2 — — 1 1

— MALIGNANT LYMPHOMA : — — — — — — 1 — — —

—————————————————————————————————————————————————————————————————————————

SUBLINGUAL GLANDS : 15 14 1 — 16 18 1 — 18 16

—————————————————————————————————————————————————————————————————————————

MANDIBULAR GLANDS : 20 20 1 — 20 20 1 — 20 20

—————————————————————————————————————————————————————————————————————————

PANCREAS : 20 20 1 — 19 20 1 — 20 20

— Atrophy;acinar;focal: 10 6 — — 7 8 — — 7 3

— Basophilia; focal : — — — — — — — — 1 3

— Fibrosis; Islets : 1 — — — — — — — 2 —

— Hemorrhage; Islets : 1 — — — 3 — — — 3 —

— Hemosiderin; Islets : 7 — — — 5 — — — 8 —

— Infiltrate; lymphoid: — — — — — — — — 1 —

—————————————————————————————————————————————————————————————————————————

THYROID GLANDS : 20 19 1 — 18 20 1 — 20 20

— C—CELL ADENOMA : — — — — — — — — 1 1

— Cyst(s) : — — — — 1 — — — — —

— FOLLIC. CELL ADENOMA: — — — — — 1 — — — —

— Hyperpl;C—cell;diff : 5 9 — — 11 8 — — 6 7

— Hyperpl;C—cell;focal: 3 2 — — 1 3 — — 2 1

—————————————————————————————————————————————————————————————————————————

PARATHYROID GLANDS : 18 17 1 — 15 18 1 — 19 18

—————————————————————————————————————————————————————————————————————————

ADRENAL GLANDS : — — — — — 1 — — — 1

—————————————————————————————————————————————————————————————————————————

—————————————————————————————————————————————————————————————————————————

ADRENAL CORTICES : 20 20 1 — 20 20 1 1 20 20

— Degeneration; cystic: — 4 — — — 2 — 1 — 9

— Extracaps; cort tiss: 2 2 — — 1 3 — — 1 —

— Hemorrhage : — 2 — — — — — — — 1

— Hyperpl;subcaps; foc: — — — — — — — — — 2

— Hyperplasia; focal : — 2 — — — 1 — — 1 1

— Hypertrophy; focal : — 1 — — 1 2 — — — 1

— Vacuolation : — — — — 1 — — — 2 —

— Vacuolation; focal : 1 — — — — — — — 2 —

—————————————————————————————————————————————————————————————————————————

ADRENAL MEDULLAS : 20 20 1 — 20 20 1 1 20 20

— Hyperplasia; focal : — — — — 1 — — — — —

—————————————————————————————————————————————————————————————————————————

SKELETAL MUSCLE : 20 20 1 — 20 20 1 — 20 20

— Infl. cell foci : 1 2 — — 4 2 — — 3 2

—————————————————————————————————————————————————————————————————————————

TONGUE : 20 20 1 1 20 20 1 — 20 20

— Hemorrhage : — — — — — 1 — — — —

— Infl. cell foci : — — — — — — 1 — — —

—————————————————————————————————————————————————————————————————————————

SCIATIC NERVE : 20 20 1 — 20 20 1 — 20 20

— Degeneration; axonal: — — — — — — 1 — — —

—————————————————————————————————————————————————————————————————————————

SKIN/SUBCUTIS : 20 20 1 2 20 19 2 — 20 20

— Atrophy; adnexa : 1 — — — — — — — — —

— Crust : — 1 — — — 1 — — — —

— Epidermoid cyst : — — — — 1 — — — 1 —

— Fibrosis : — — — — 1 — — — — —

— Infilt; mononuclear : 2 — — — — — — — — —

— Inflamm; granulomat : 1 — — — — — — — — —

— Ulceration : 1 — — — — 1 — — — —

—————————————————————————————————————————————————————————————————————————

MAMMARY GLAND AREA : 20 20 1 3 20 19 1 — 20 20

— ADENOCARCINOMA : — — — 1 — — — — — —

— Dilatation; gland : — 1 — — — 1 — — 1 —

— FIBROADENOMA : — 2 — — — — — — — —

— Galactocele : — 1 — 1 — 1 — — — —

— Hyperpl; duct; focal: — 2 — — — — — — — —

— Hyperpl;gland; focal: — 1 — 1 — — — — — —

— Hyperplasia; diffuse: — — — — — — — — — 3

—————————————————————————————————————————————————————————————————————————

EYES : 20 20 1 — 20 20 1 — 20 20

— Atrophy; retina : 9 18 — — 6 17 — — 4 14

— MALIGNANT LYMPHOMA : — — — — — — 1 — — —

—————————————————————————————————————————————————————————————————————————

HARDERIAN GLANDS : 20 20 1 — 20 20 1 — 20 20

— Atrophy; focal : — 1 — — — — — — — —

— Hemorrhage : — — — — — — — — — 1

— Hyperplasia; focal : — 3 — — 1 1 — — — 2

— Infiltrate; lymphoid: 3 3 1 — 2 1 — — 1 3

— Inflammat/necrosis : — — — — — 1 — — — —

— Pigment; brown : 19 11 1 — 16 16 — — 17 10

—————————————————————————————————————————————————————————————————————————

LACRIMAL GLANDS : 20 20 4 — 20 20 3 — 20 20

— Atrophy; focal : — 1 — — — — — — 2 —

— Degeneration/inflamm: — — — — — — — — — 1

— Hemorrhage : 2 — — — 3 — — — 3 —

— Infiltrate; lymphoid: 12 1 1 — 11 — — — 13 1

— Metaplasia;Harderian: 18 4 4 — 15 4 3 — 17 2

—————————————————————————————————————————————————————————————————————————

—————————————————————————————————————————————————————————————————————————

BRAIN : 20 20 1 1 20 20 1 — 20 20

— Compression by pit. : — — 1 1 — — — — 1 —

— MALIGNANT LYMPHOMA : — — — — — — 1 — — —

—————————————————————————————————————————————————————————————————————————

PROSTATE GLAND : 20 — 1 — 20 — 2 — 20 —

— Atrophy : — — 1 — — — — — — —

— Infiltrate; lymphoid: — — — — 4 — — — 4 —

— Infiltrate; neutroph: — — — — 4 — — — 4 —

— Vacuolation; epithel: 1 — — — 2 — — — 1 —

—————————————————————————————————————————————————————————————————————————

COAGULATING GLANDS : 20 — 1 — 20 — 1 — 20 —

— Atrophy : — — 1 — — — — — — —

—————————————————————————————————————————————————————————————————————————

SEMINAL VESICLES : 20 — 1 — 20 — 2 — 20 —

— Atrophy : — — 1 — — — — — — —

— Dilatation : — — — — 2 — — — — —

—————————————————————————————————————————————————————————————————————————

UTERUS : — 20 — 1 — 20 — — — 20

— Dilatation; gland : — 9 — — — 3 — — — 8

— Dilatation; lumen : — — — — — 3 — — — 1

— ENDOMET.STROM. POLYP: — — — 1 — 1 — — — —

— Hyperpl;cystic endom: — — — — — 2 — — — —

— Metapl;squam;glands : — 9 — — — 4 — — — 7

—————————————————————————————————————————————————————————————————————————

CERVIX : — 20 — — — 20 — — — 20

—————————————————————————————————————————————————————————————————————————

VAGINA : — 16 — — — 13 — — — 12

— Fibrosis : — — — — — 1 — — — —

— Keratinised epithel.: — 4 — — — — — — — 2

— Mucification : — 5 — — — 6 — — — 6

—————————————————————————————————————————————————————————————————————————

PITUITARY GLAND : 20 20 1 4 20 20 1 — 20 20

— ADENOMA; PARS ANTER : 2 3 1 2 3 3 — — 3 1

— Angiectasis : — 2 — 1 — — — — 1 —

— Cyst; Pars Anterior : 2 1 — — — — — — 1 1

— Cyst;Pars Intermedia: — — — — 1 — — — — —

— Hyperpl;focal;P. Ant: 1 2 — 1 2 3 — — 4 1

— Vacuol; focal; PA : — 1 — — — — — — — —

— Vacuolated cells; PA: — — — — — — — — 1 —

—————————————————————————————————————————————————————————————————————————

SPINAL CORD : 20 20 1 — 20 20 1 — 20 20

— Degen; nerve roots : — — — — — — — — — 1

— Hemorrhage; agonal : — — — — — 1 — — — —

—————————————————————————————————————————————————————————————————————————

BONE, STERNUM : 20 20 1 — 20 20 1 — 20 20

— Degen;chondromucin. : — — — — — — — — — 1

—————————————————————————————————————————————————————————————————————————

BONE MARROW, STERNUM : 20 20 1 — 20 20 1 — 20 20

— Degen;chondromucin. : — — — — — 1 — — 1 —

— MALIGNANT LYMPHOMA : — — — — — — 1 — — —

—————————————————————————————————————————————————————————————————————————

FEMUR/JOINT : 20 20 1 — 20 20 1 — 20 20

— Degen;chondromucin. : — — — — 1 — — — 1 —

— Osteomalacia : — — — — — — — — 1 —

— MALIGNANT LYMPHOMA : — — — — — — 1 — — —

—————————————————————————————————————————————————————————————————————————

CLITORAL GLANDS : — — — — — — — — — 1

— Cyst : — — — — — — — — — 1

—————————————————————————————————————————————————————————————————————————

—————————————————————————————————————————————————————————————————————————

PREPUTIAL GLANDS : 1 — 2 — — — — — — —

— Inflammation : — — 2 — — — — — — —

—————————————————————————————————————————————————————————————————————————

URETERS : — — — — — — — 1 — —

— Dilatation : — — — — — — — 1 — —

—————————————————————————————————————————————————————————————————————————

LYMPH NODES : — — — — — — 1 — — —

— MALIGNANT LYMPHOMA : — — — — — — 1 — — —

—————————————————————————————————————————————————————————————————————————

MEDIASTINAL TISSUE : — — — — — — — 1 — —

— Infilt; neutrophil : — — — — — — — 1 — —

—————————————————————————————————————————————————————————————————————————

MEDIAST. LYMPH NODE : 1 — — — — — 1 — — —

— Hemorrhage : 1 — — — — — — — — —

— Pigment; hemosiderin: 1 — — — — — — — — —

— MALIGNANT LYMPHOMA : — — — — — — 1 — — —

—————————————————————————————————————————————————————————————————————————

BODY CAVITIES : — — — — — — 3 — — —

— Fat necrosis : — — — — — — 1 — — —

—————————————————————————————————————————————————————————————————————————

ABDOMINAL CAVITY : 7 7 3 4 6 5 6 5 5 6

— Epidermoid cyst : — — 1 — — — — — — —

— Fat necrosis : 3 6 — 1 3 3 1 5 2 2

— Hemorrhage : 4 2 2 3 4 3 4 — 2 2

— Metastasis : — — — — — — — — — 1

—————————————————————————————————————————————————————————————————————————

BONE : — — — — — — — — — 1

— OSTEOSARCOMA : — — — — — — — — — 1

—————————————————————————————————————————————————————————————————————————

CL, centrilobular; PP, periportal; urothel, urothelium; G.A.L.T., gut associated lymphoid tissue; hyperpl, hyperplasia; epithel, epithelial; foc, focal; ep, epithelium; submuc, submucosal; alv/br, alveolar/bronchial cell; alveol, alveolar cell; mesoth, mesothelium; NOS, not otherwise specified; SCS, sex cord stromal; infilt, infiltrate; follic., follicular; extracaps cort tiss, extracapsular cortical tissue; subcaps, subcapsular; infl., inflammatory; inflamm, inflammation; granulomat, granulomatous; pit., pituitary neoplasm; neutroph, neutrophil; endom(et), endometrial; strom, stromal; metapl, metaplasia; squam, squamous; PA (P. Ant.), pars anterior; degen, degeneration; chondromucin., chondromucinous

**Table 13:** Non-neoplastic lesions in male and female Wistar Han RCC rats in the carcinogenicity phase of the combined chronic toxicity/carcinogenicity feeding trial with GM maize NK603 at an inclusion rate of 11 and 33% in the diet

| **Group** | **Isogenic maize**  **(% of diet)** | **NK603 only**  **(% of diet)** | **NK603 +**  **Roundup**  **(% of diet)** | **No. of**  **Males** | **No. of**  **Females** |
| --- | --- | --- | --- | --- | --- |
| **1** | 33 | 0 | 0 | 50 | 50 |
| **2** | 22 | 11 | 0 | 50 | 50 |
| **3** | 0 | 33 | 0 | 50 | 50 |
| **4** | 22 | 0 | 11 | 50 | 50 |
| **5** | 0 | 0 | 33 | 50 | 50 |

—————————————————————————————————————————————————————————————————————————

DOSE GROUP: 1 2 3 4 5

—————————————————————————————————————————————————————————————————————————

GENERAL OBSERVATIONS : 12 2 6 8 10 5 6 8 8 8

— Autolysis : 9 2 6 6 6 5 5 6 7 5

—————————————————————————————————————————————————————————————————————————

SYSTEMIC NEOPLASMS : — — 2 1 — 1 2 1 1 1

—————————————————————————————————————————————————————————————————————————

HEART : 50 50 15 27 50 50 20 20 50 50

— Bacterial colonies : — — — — — — — — 1 —

— Blood clot : — — 1 — 3 1 — — — 1

— Cardiomyopathy : 41 17 14 8 39 11 13 7 41 18

— Hyperpl;Schwann cell: — 1 — — — — — — — —

— Hypertrophy; myocard: 5 — 2 — 1 3 1 — 10 2

— Thrombus : — 1 — — — 1 — — — —

—————————————————————————————————————————————————————————————————————————

AORTA : 50 50 14 26 50 50 18 19 50 50

— Dilatation : — — 1 — — — — — 2 —

— Mineralisation : — — 1 — — — — — 2 —

—————————————————————————————————————————————————————————————————————————

ESOPHAGUS : 50 50 14 26 50 50 18 19 50 50

— Hyperkeratosis : 8 3 2 5 6 8 4 2 9 7

— Ulceration : — — — — 1 — — — — —

—————————————————————————————————————————————————————————————————————————

TRACHEA : 50 50 14 26 50 50 18 19 50 50

— Exudate;neutro;lumen: 1 — — — — — — — — —

—————————————————————————————————————————————————————————————————————————

LIVER : 50 50 28 35 50 50 26 38 50 50

— Altered focus; clear: 1 4 1 — 6 — 1 — 4 1

— Altered focus; eosin: 26 9 10 1 21 15 3 2 21 11

— Altered focus; mixed: 10 7 2 — 13 8 1 1 6 3

— Altered focus;basoph: 7 27 1 11 19 26 — 7 8 33

— Altered focus;vacuol: 9 4 2 1 14 1 1 2 7 2

— Angiectasis : 1 1 3 2 7 1 2 3 5 3

— Atrophy; diffuse : — — — 1 3 — — — 1 —

— Congestion : 2 — 1 1 4 2 4 1 7 1

— Cyst; biliary : 4 7 — 2 3 2 — 2 3 8

— Dilatation;bile duct: 1 — — — — — — 2 — —

— Cyst; squamous : 1 — — — — — — — — —

— Degen/necrosis; CL : 3 2 2 3 3 2 1 4 6 3

— Degeneration; cystic: 5 1 2 — 1 — — — 4 —

— Fibrosis; bile duct : 5 4 — 1 5 11 — — 6 8

— Fibrosis; lobule : — — — 1 — — — — — —

— Hematopoiesis : 2 5 — 4 — 3 2 1 1 3

— Hepatodiaph. nodule : — — — — — — — 1 — —

— Hyperpl; bile duct : 4 10 — 3 3 13 — 3 7 16

— Hyperpl; oval cell : — — — — — — — — 2 —

— Hypert;hepatoc;diff : — 1 — — — — — — — —

— Hypert;hepatoc;PP : — — — — — 1 — — — —

— Infarction : 1 — — — — — — — — —

— Infl. cell foci : 21 28 3 8 16 15 — 3 23 15

— Leucocytosis : — — — 1 — — — — — —

— Necrosis; focal : 2 1 — 1 1 — — 1 3 2

— Pigment; hepatocytes: — 3 — 1 — 2 — 2 2 4

— Pigment; macrophages: 1 6 — 2 — 5 4 6 4 4

— Tension lipidosis : 2 2 1 3 2 3 — 1 — 2

— Thrombus : — 1 — — — — — — — —

— Vacuolation; CL : — 1 — — — 2 1 — 3 1

— Vacuolation; PP : 11 18 4 6 14 18 7 4 14 9

— Vacuolation; diffuse: 5 2 4 5 4 4 1 4 4 5

— Vacuolation; focal : 9 3 1 — 3 7 — 1 11 2

—————————————————————————————————————————————————————————————————————————

SPLEEN : 50 50 19 29 50 50 24 25 50 50

— Arteritis : — — — — — — — — 1 —

— Bacterial colonies : — — — — — — — — 1 —

— Congestion : 2 — 1 2 — 1 2 1 — 1

— Fibroplasia : — — — — 1 — — — — —

— Hematopoiesis : 40 42 9 17 37 35 10 14 36 42

— Hemosiderin : 46 44 12 22 40 45 16 17 44 44

— Incr.; megakaryocyte: — — — — — — — — — 1

— Infilt; histiocyte : — — — — — 1 — — — —

— Infilt; neutrophil : — — — — — — 1 — 1 —

— Lymphoid atrophy : 1 3 — — 1 — 1 1 2 —

— Thrombus : — 1 — — — — — 2 — —

— Accessory tissue : — — 1 — — — — — — —

—————————————————————————————————————————————————————————————————————————

MESENT. LYMPH NODE : 50 50 17 25 50 50 19 21 49 49

— Angiectasis : 1 — — — — — — — — —

— Atrophy; lymphoid : — — — — — — — — 1 —

— Ceroid : 39 41 7 16 37 37 9 15 32 31

— Dilation; sinusoid : 5 5 2 — 1 — 1 1 3 3

— Erythrocytosis : 17 13 4 5 16 16 3 2 17 15

— Fibrosis : — — — — — — — — 1 —

— Hyperpl;histioc;foc : — — — — — 1 — — — —

— Infilt; plasma cell : 1 1 — 1 — 1 — — — —

— Pigment; hemosiderin: 10 21 2 7 15 28 4 7 13 16

—————————————————————————————————————————————————————————————————————————

KIDNEYS : 50 50 28 31 50 50 27 23 50 49

— Chron. Prog. Nephrop: 48 28 23 9 44 33 21 11 46 28

— Cyst; cortex : — 2 — 1 — — — — 2 —

— Cyst; medulla : — — — — 1 — — — — —

— Dilatation; pelvis : — — — — — — 1 — — —

— Hemocyst; pelvis : — 3 — — — — — 1 1 —

— Hemorrhage; agonal : — — — — — 1 — — 1 —

— Hyperplasia; urothel: 2 1 1 1 2 3 1 — 2 3

— Hypert;tubule;focal : — — — — — 1 — — — —

— Infiltrate; lipocyte: — — — — 1 — — — — —

— Infiltrate; lymphoid: 1 2 — — 1 — — — 1 —

— Inflammation; pelvis: 4 — 3 1 4 2 1 2 2 4

— Metaplasia; osseous : — — — — — — 1 — — —

— Mineral; pelvis : — 2 — — — 2 — — — —

— Scar; cortex : — — — — — — 1 — — —

— Pigment; cortex : 2 8 1 3 1 11 — 1 2 4

— Tubular basophilia : — — — — — 1 — — — —

— Vacuolation; cortex : — 1 — — — 1 — — — —

—————————————————————————————————————————————————————————————————————————

—————————————————————————————————————————————————————————————————————————

URINARY BLADDER : 50 50 15 25 50 50 18 20 50 49

— Inflammation; acute : 2 — — 1 — — — — — —

— Urothel.hyperpl;diff: — — 1 — 1 — 1 — — —

—————————————————————————————————————————————————————————————————————————

DUODENUM : 50 50 14 26 50 50 18 20 50 50

— Congestion : 1 — — — 1 — — — 1 —

— Hemorrhage : — 1 2 — — — — — — 1

— Hyperpl;muscle;focal: — — — — — — — — — 1

— Intussusception : — — — — — — — — 1 —

— Arteritis : — — — — — — 1 — — —

—————————————————————————————————————————————————————————————————————————

JEJUNUM : 50 50 16 27 49 50 19 21 50 50

— Congestion : 2 — — — — — — — — —

— Hemorrhage : — — — 1 — — — — — —

— Hyperpl;muscle;focal: — — — 1 — 1 — — — —

— Dilatation; lumen : — — 1 — — — — — — —

—————————————————————————————————————————————————————————————————————————

ILEUM : 50 49 14 26 50 49 18 20 50 49

— Hemorrhage : — — — 1 — — — — — —

— Inflamm; necrotizing: — — — 1 — — — — — —

—————————————————————————————————————————————————————————————————————————

G.A.L.T. : 34 26 13 15 33 30 16 16 32 31

— Lipid : 1 — — — 1 1 — — 2 1

— Mineralisation : — — 1 — — — — — — —

—————————————————————————————————————————————————————————————————————————

CECUM : 50 50 15 26 50 50 18 19 50 49

— Diverticulum : — 1 — — — — — — — —

— Hemorrhage : — — 1 — — — — — — —

— Fibrosis : — — — — — — 1 — — —

— Erosion : — — — — — — 1 — — —

—————————————————————————————————————————————————————————————————————————

COLON : 50 50 14 26 50 50 18 20 50 50

—————————————————————————————————————————————————————————————————————————

RECTUM : 50 50 14 26 50 50 18 19 50 50

— Hemorrhage : — 1 — — — — — — — —

—————————————————————————————————————————————————————————————————————————

STOMACH : — — 2 — — — — 1 1 1

—————————————————————————————————————————————————————————————————————————

FORESTOMACH : 50 50 18 27 50 50 18 20 50 50

— Cyst : — — — — 1 — — — — —

— Edema : 2 3 1 4 3 5 1 2 3 1

— Erosion : 2 — 2 6 2 1 1 3 — —

— Hemorrhage : — — — — — — 2 — — 1

— Hyperpl;epithel;L.R.: 1 1 — — — — — 1 — —

— Hyperpl;epithel;diff: 6 4 5 4 6 5 5 3 9 4

— Hyperpl;epithel;foc : — — 1 — — 1 1 — — 1

— Infiltrate; lipocyte: 5 2 2 1 6 3 — — 2 4

— Inflammation; serosa: — — 1 — — — — — 1 —

— Inflammation; submuc: 4 2 1 5 — 6 — 4 5 3

— Ulceration : 3 2 3 1 4 4 4 2 6 1

—————————————————————————————————————————————————————————————————————————

GLANDULAR STOMACH : 50 50 19 27 50 50 19 20 50 50

— Atrophy; gland : 1 — 1 4 1 2 1 — — —

— Congestion : 1 — — — — — — — 1 —

— Degeneration; glands: — — — 1 — 1 — — 1 —

— Dilated glands : 12 18 2 3 6 12 — 2 17 13

— Ectopic glands : — — — — 1 — — — — —

— Ectopic squamous ep : — — — — — — — — — 1

— Edema : — 1 1 — 1 — — — — —

— Erosion : 2 6 2 2 3 2 — — 6 5

— Fibrosis : — — — — 1 — — — 4 —

— Hemorrhage : 1 1 1 — — 1 — — 3 —

— Hyperpl;Brunner's gl: 1 — — — — — — — 1 —

— Hyperplasia; glands : — — — — — — — — 1 —

— Infiltrate; lipocyte: — — — 1 — — — — 1 2

— Inflammation; submuc: — — 1 — 1 — — — — 2

— Mineralisation : — — — — — — — — 2 —

— Ulceration : — — — — — — — — 1 —

—————————————————————————————————————————————————————————————————————————

LUNG : 50 50 25 35 50 50 24 34 50 50

— Agonal hemorrhage : — 1 — — — 1 1 1 1 1

— Alveolar macrophages: 32 45 12 24 40 44 15 24 35 43

— Arteritis : — — — — — — — — 1 —

— Congestion : 5 1 6 1 9 1 3 — 2 1

— Adhesion; pleura : — — — — — — 1 — — —

— Edema : 1 1 4 1 3 — 3 — 2 —

— Embolus; septic : 1 — — — — — — — — —

— Hemorrhage : — — — 1 — — — — — —

— Hyperpl; foc; alveol: — — — — — — — — — 2

— Inflammation; alveol: — — 1 — 1 — — 1 — —

— Inflammation; septal: — — 1 — — — — — 2 —

— Leucocytosis : 1 1 — — — — — — — —

— Osseous metaplasia : — — — — 1 1 — — — —

— Pigment; hemosiderin: — — — — — 1 — — — —

— Thrombus : — — — — — — — — — 1

—————————————————————————————————————————————————————————————————————————

THYMUS : 47 46 14 49 48 45 22 50 44 47

— Hyperpl; mixed; foc : — — — — 2 1 1 1 — —

— Hyperpl;lymphoid;dif: — — — — — — — — 2 1

— Hyperpl;lymphoid;foc: 1 — — — — — 1 — — 2

— Agonal hemorrhage : 3 5 3 3 2 3 4 4 3 1

— Atrophy; lymphoid : 21 9 9 19 26 11 10 16 24 18

— Necrosis/apoptosis : — 3 — — — 1 — — — —

—————————————————————————————————————————————————————————————————————————

TESTES : 50 — 18 — 50 — 23 — 50 —

— Arteritis : 4 — 4 — 2 — 2 — 7 —

— Atrophy; tubule : 7 — 5 — 14 — 11 — 13 —

— Edema : — — 1 — 1 — — — — —

— Hemorrhage : — — — — — — — — 1 —

— Hyperpl;Leydig;focal: 1 — — — — — — — — —

— Sperm'cele granuloma: — — — — 1 — — — — —

—————————————————————————————————————————————————————————————————————————

EPIDIDYMIDES : 50 — 17 — 50 — 20 — 50 —

— Arteritis : 1 — 1 — — — — — — —

— Cellular debris : 2 — — — — — 1 — 4 —

— Decrease spermatozoa: 4 — 2 — 3 — 5 — 8 —

— Granuloma : 1 — — — 1 — 1 — — —

— Hyperpl;mesoth;focal: 1 — — — — — — — 1 —

— Inflammation : — — 1 — — — — — — —

— Vacuolation; focal : 2 — — — — — 1 — 5 —

—————————————————————————————————————————————————————————————————————————

OVARIES : — 50 — 30 — 50 — 21 — 50

— Atrophy : — — — 1 — 1 — — — —

— Corpora lutea : — 33 — 20 — 36 — 13 — 33

— Cyst; NOS : — 1 — — — 1 — 1 — 1

— Cyst; Rete ovarii : — 1 — — — 1 — — — —

— Cyst; bursal : — 1 — — — — — — — —

— Cyst; follicular : — 12 — — — 7 — 2 — 5

— Cyst; luteal : — 3 — — — 2 — — — 2

— Cyst; parovarian : — — — 1 — 1 — — — 1

— Foc.granulos.hyperpl: — — — — — — — — — 1

— Focal SCS hyperpl : — 12 — 5 — 9 — 2 — 5

— Hyperpl; Rete ovarii: — — — — — 1 — — — —

— Hyperpl;SCS; diffuse: — 12 — 2 — 10 — 2 — 10

—————————————————————————————————————————————————————————————————————————

—————————————————————————————————————————————————————————————————————————

MANDIB.LYMPH NODES : 49 49 17 31 50 49 26 25 50 48

— Abscess : — — — — 1 — — — — —

— Congestion : 2 — 1 — 2 — 3 1 2 —

— Dilatation; sinusoid: 11 4 2 1 15 3 9 1 14 4

— Erythrocytosis : 13 17 4 10 17 11 9 5 14 14

— Granuloma : — — — — — — — — 1 —

— Infilt; plasma cell : 22 24 6 13 30 31 16 13 24 33

— Infilt;macrophage : 1 — — — — — — — — —

— Lymphoid atrophy : — — — — — — — — — 1

— Lymphoid hyperplasia: — — — — — — — 1 — 1

— Pigment; hemosiderin: 6 12 — 7 11 11 1 5 6 10

— Hematoma : — — — 1 — — — — — —

—————————————————————————————————————————————————————————————————————————

SUBLINGUAL GLANDS : 49 48 12 25 45 46 17 20 49 48

— Atrophy : — — — 1 — 2 — — 2 3

— Dilatat;duct/acinus : — — — — — 1 — — — —

— Inflammat/necrosis : — — — — — 1 — — — —

—————————————————————————————————————————————————————————————————————————

MANDIBULAR GLANDS : 50 49 13 25 50 49 17 20 50 49

— Atrophy : — — 1 1 1 2 — — 3 2

—————————————————————————————————————————————————————————————————————————

PANCREAS : 49 50 14 26 50 50 20 22 49 50

— Apoptosis : — — — — — — — — 1 —

— Arteritis : 2 — 1 — — — — — 1 —

— Atrophy;acinar;diff : 1 — 1 1 1 — — 1 4 3

— Atrophy;acinar;focal: 21 16 7 7 21 12 2 3 17 12

— Basophilia; focal : 5 5 — — 3 2 — — 2 1

— Cyst(s) : — 1 — — — 1 — — 2 —

— Fat necrosis : — 1 — — — — — — — —

— Fibrosis; Islets : — — 1 — 1 — — — 1 —

— Hemorrhage; Islets : 1 — — — 2 — — — 1 —

— Hemorrhage; acinar : — — — — — — — — 1 —

— Hemosiderin; Islets : 14 — 1 1 14 1 1 — 23 —

— Hyperpl;acinar;focal: 1 — — — 1 2 1 — 1 —

— Infiltrate; lipocyte: 32 22 4 6 32 17 3 1 27 28

— Infiltrate; lymphoid: — — — — — 1 — — — 1

— Infiltrate; mononuc : — — — — — — — — 1 —

— Inflamm; chronic : — — 1 — — — — — 1 —

— Islet hyperpl; diff : 3 — — — — 1 — — 7 1

— Islet hyperpl; focal: 5 1 2 2 10 1 1 1 8 5

—————————————————————————————————————————————————————————————————————————

THYROID GLANDS : 50 49 17 24 50 50 21 21 49 49

— Cyst(s) : 1 — — — — — 1 1 — 1

— Hyperpl;C—cell;diff : 36 30 10 10 32 30 5 4 46 37

— Hyperpl;C—cell;focal: 7 4 1 1 9 5 3 — 6 6

— Hyperpl;follic;focal: 2 1 — 1 3 1 — — — 2

—————————————————————————————————————————————————————————————————————————

PARATHYROID GLANDS : 49 45 14 22 48 49 16 20 49 49

— Arteritis : 1 — — — — — — — — —

— Hyperplasia; diffuse: — — 2 — 1 — 1 — 5 —

— Hyperplasia; focal : — — — — 1 — — — — —

—————————————————————————————————————————————————————————————————————————

ADRENAL GLANDS : 3 — 2 — 2 1 2 1 1 —

—————————————————————————————————————————————————————————————————————————

ADRENAL CORTICES : 50 50 22 35 50 50 22 31 50 50

— Atrophy : — — 1 — 1 1 1 — — —

— Degeneration; cystic: 2 36 4 22 6 40 1 23 3 35

— Extracaps; cort tiss: 9 7 2 3 10 4 — — 6 7

— Hematopoiesis : 2 2 — 4 — 5 1 2 — 3

— Hemorrhage : — — — — — 1 — — — 1

— Hyperpl;subcaps; foc: 3 10 2 — 4 7 1 2 6 4

— Hyperplasia; focal : 5 4 — 2 12 5 4 5 9 7

— Hypertrophy; diffuse: 1 3 1 3 1 — — — — 2

— Hypertrophy; focal : 5 10 3 7 9 8 1 2 7 7

— Leucocytosis : — 1 — — — — — — — —

— Necrosis; focal : — 1 — — — — — — — —

— Thrombus : — — — — 1 1 — — — —

— Vacuolation : 1 1 — — — — — — 2 —

— Vacuolation; focal : 21 2 6 2 18 1 4 1 18 5

—————————————————————————————————————————————————————————————————————————

ADRENAL MEDULLAS : 50 48 20 35 50 50 22 30 50 50

— Degeneration; cystic: — — — — — — — — 1 —

— Hyperplasia; focal : 2 1 1 1 4 3 2 — 5 —

—————————————————————————————————————————————————————————————————————————

SKELETAL MUSCLE : 50 50 14 26 50 50 18 20 50 50

— Atrophy; diffuse : — — — — — — — — 3 —

— Infl. cell foci : 5 6 — 6 7 5 3 — 5 8

—————————————————————————————————————————————————————————————————————————

TONGUE : 50 50 15 25 50 50 18 20 50 50

— Arteritis : 2 — 1 — — — — — 2 —

— Thrombus : 1 — 2 — — — — — — —

— Vacuol;salivary gl : — — — — 1 — 1 1 3 —

—————————————————————————————————————————————————————————————————————————

SCIATIC NERVE : 50 50 14 26 50 50 18 20 50 50

— Degeneration; axonal: 8 5 3 3 14 10 3 4 7 7

—————————————————————————————————————————————————————————————————————————

SKIN/SUBCUTIS : 49 50 21 28 50 50 24 23 50 50

— Abscess : — — 1 — — — 1 1 1 —

— Acanthosis : — — 1 — — 1 — — — —

— Atrophy; adnexa : — — — 1 — 1 — 2 1 1

— Crust : 1 4 — — 1 2 — 2 2 3

— Cystic follicles : — — 1 — — — — — — —

— Edema : — — — — — — — — — 1

— Epidermoid cyst : 3 — 2 1 6 — 2 1 4 —

— Fibroplasia : — — — — 2 — — — — —

— Fibrosis : — — — — 1 — — — 1 —

— Hemorrhage; subcutis: — — — — — — — — 1 —

— Infilt; mononuclear : — — — — — 1 — — — —

— Inflamm; granulomat : — — — — 2 — — — 2 —

— Hyperkeratosis : — — — — — — — 1 — —

— Hyperplasia; epiderm: — 1 — — — — — — — —

— Ulceration : — 4 — — — 1 1 2 — 2

—————————————————————————————————————————————————————————————————————————

MAMMARY GLAND AREA : 49 50 14 38 50 49 18 31 50 50

— Abscess : — — — — — — — 1 — —

— Cyst : — 1 — — — — — — — —

— Dilatation; gland : — 1 — — — 3 — 2 1 1

— Galactocele : — 4 1 2 — 3 — — — 2

— Hyperpl;gland; focal: 1 1 — 2 — 2 — — — 7

— Hyperplasia; diffuse: — 9 — 8 — 7 — 5 — 5

—————————————————————————————————————————————————————————————————————————

EYES : 49 50 14 25 50 50 18 20 50 50

— Atrophy; retina : 32 38 2 18 25 33 3 13 32 40

— Degeneration; lens : 1 — — — 1 — — — — —

— Fibrovascularization: — — — — 1 — — — — —

— Hemorrhage : — — — — — — — — — 1

— Inflammation; acute : 1 — — — — — — — — —

— Keratitis : 1 — — — — — — — — —

— Panophthalmitis : — — — — — — — — — 1

—————————————————————————————————————————————————————————————————————————

—————————————————————————————————————————————————————————————————————————

HARDERIAN GLANDS : 49 50 15 25 50 50 18 20 50 50

— Atrophy; diffuse : — — — — 1 — — — 1 —

— Atrophy; focal : 2 1 — — 1 1 — — — —

— Hyperplasia; focal : 11 6 3 1 13 1 3 1 15 1

— Infiltrate; lymphoid: 5 4 — 1 2 8 — 1 2 5

— Inflammat/necrosis : 1 1 — — 2 1 — — 1 1

— Pigment; brown : 48 43 12 18 50 45 16 16 49 43

—————————————————————————————————————————————————————————————————————————

LACRIMAL GLANDS : 50 50 15 26 50 50 17 20 50 50

— Atrophy; diffuse : 1 2 — — — 1 — — 1 1

— Atrophy; focal : — 2 — — — — — — — 1

— Degeneration/inflamm: — — — — — — — — — 1

— Infiltrate; lymphoid: 5 2 2 — 4 1 — — 6 1

— Inflamm./necrosis : — — — — — 1 — — — —

— Metaplasia;Harderian: 49 17 11 5 48 19 17 3 49 17

—————————————————————————————————————————————————————————————————————————

BRAIN : 50 49 16 29 50 50 19 23 50 50

— Compression by pit. : 13 14 7 18 11 19 9 17 19 12

— Hemorrhage : — — — — — — — — 3 —

— Inflamm; meninges : — — — — — — 1 — — —

—————————————————————————————————————————————————————————————————————————

PROSTATE GLAND : 50 — 15 — 49 — 18 — 50 —

— Arteritis : — — — — — — — — 1 —

— Atrophy : 16 — 4 — 11 — 5 — 13 —

— Hyperplasia; focal : 2 — 2 — — — — — 3 —

— Infiltrate; lymphoid: 7 — 5 — 10 — 2 — 18 —

— Infiltrate; neutroph: 20 — 5 — 22 — 6 — 27 —

— Inflammation : 4 — 2 — — — 2 — 3 —

— Vacuolation; epithel: — — — — 3 — — — — —

—————————————————————————————————————————————————————————————————————————

COAGULATING GLANDS : 48 — 15 — 48 — 18 — 49 —

— Atrophy : 15 — 6 — 11 — 7 — 13 —

— Infiltrate; neutroph: 1 — — — — — — — — —

— Inflammation : 1 — — — — — — — 2 —

— Spermatocele : 1 — — — — — — — — —

—————————————————————————————————————————————————————————————————————————

SEMINAL VESICLES : 50 — 15 — 49 — 18 — 50 —

— Atrophy : 16 — 6 — 11 — 6 — 15 —

— Hemorrhage : — — — — — — 1 — — —

— Hyperplasia; focal : 2 — — — — — — — — —

— Infiltrate; neutroph: 1 — — — — — — — — —

— Inflammation : 1 — — — — — — — — —

—————————————————————————————————————————————————————————————————————————

UTERUS : — 50 — 27 — 50 — 22 — 49

— Dilatation; gland : — 13 — 5 — 10 — 3 — 8

— Dilatation; lumen : — 1 — 2 — 3 — 1 — 2

— Fibrosis : — 1 — — — — — — — —

— Angiectasis : — — — — — 1 — — — —

— Hyperpl;cystic endom: — 2 — — — 1 — — — 1

— Hyperpl;foc;endomet : — — — — — — — — — 1

— Metapl;squam;glands : — 5 — 1 — — — — — 1

—————————————————————————————————————————————————————————————————————————

CERVIX : — 50 — 26 — 49 — 19 — 49

— Fibrosis : — 1 — 1 — 1 — — — 2

—————————————————————————————————————————————————————————————————————————

VAGINA : — 48 — 24 — 47 — 16 — 46

— Cyst : — — — — — — — — — 1

— Fibrosis : — 1 — — — — — 1 — 1

— Keratinised epithel.: — 4 — 1 — — — 1 — 1

— Mucification : — 31 — 14 — 31 — 5 — 30

—————————————————————————————————————————————————————————————————————————

—————————————————————————————————————————————————————————————————————————

PITUITARY GLAND : 50 50 19 38 50 50 26 32 50 50

— Angiectasis : 1 3 1 1 1 1 — — — 1

— Atrophy; diffuse : — — — — — — — — 1 —

— Cyst; Pars Anterior : 1 — — — 2 — — — 1 —

— Hemorrhage; Pars Int: — 2 — — — — — — — —

— Hemosiderin;Pars Int: — 2 — — — — — — — —

— Hemosiderin;Pars Ant: — — — 1 — — — — — —

— Hyperpl;focal;P. Ant: 11 10 2 3 15 5 — — 6 8

— Hyperpl;diff;P. Ant : — — — — 1 — — — — —

— Hyperpl;focal;P.Int : — 1 — — — — — 1 — —

— Infilt;lipocyte;foc : — — — — — — — — 1 —

— Necrosis; Pars Anter: — — — — — — — 1 1 —

— Vacuol; focal; PA : — — — — — — — — 1 —

—————————————————————————————————————————————————————————————————————————

SPINAL CORD : 50 50 14 26 50 50 18 20 50 50

— Degen; nerve roots : 23 35 3 11 17 24 5 5 26 34

— Hemorrhage;secondary: — — 1 1 — 1 — — — —

—————————————————————————————————————————————————————————————————————————

BONE, STERNUM : 50 50 13 26 50 50 18 20 50 50

— Degen;chondromucin. : — 1 — — — — — — — 1

— Hyperostosis : — — — — — — — — 1 —

— Osteolysis;secondary: — — — — — — — — 1 —

—————————————————————————————————————————————————————————————————————————

BONE MARROW, STERNUM : 50 50 13 25 50 50 18 19 50 50

— Incr; hematopoiesis : 4 7 2 6 5 6 1 3 3 5

—————————————————————————————————————————————————————————————————————————

FEMUR/JOINT : 50 50 14 26 50 50 18 19 50 50

— Degen;chondromucin. : — — — — — 1 — — — —

— Inflammation; joint : — — — — — — — — 1 —

— Osteolysis;secondary: — — — — — — — — 1 —

—————————————————————————————————————————————————————————————————————————

BILE DUCT, EXTRAHEP. : — — — — 1 — — — 1 —

— Dilatation : — — — — 1 — — — 1 —

— Hyperplasia; diffuse: — — — — — — — — 1 —

—————————————————————————————————————————————————————————————————————————

PAROTID GLANDS : 2 1 — — — — — — 1 —

— Atrophy : 1 — — — — — — — 1 —

— Hyperplasia; focal : 1 1 — — — — — — — —

— Fibrosis; focal : 1 — — — — — — — — —

—————————————————————————————————————————————————————————————————————————

CLITORAL GLANDS : — — — 1 — — — — — —

— Cyst : — — — 1 — — — — — —

— Inflamm; suppurative: — — — 1 — — — — — —

—————————————————————————————————————————————————————————————————————————

PREPUTIAL GLANDS : 1 — — — 1 — 2 — 1 —

— Abscess : — — — — — — 1 — 1 —

— Hyperplasia; diffuse: 1 — — — — — — — — —

— Inflammation : 1 — — — — — — — — —

—————————————————————————————————————————————————————————————————————————

LYMPH NODES : — — 2 — — — 2 — 1 —

— Erythrocytosis : — — — — — — — — 1 —

— Fibrosis : — — — — — — — — 1 —

— Lymphangiectasis : — — — — — — — — 1 —

— Dilation; sinusoid : — — 1 — — — 1 — — —

—————————————————————————————————————————————————————————————————————————

AXILLARY LYMPH NODES : — 1 — — — 1 1 2 — —

— Erythrocytosis : — — — — — 1 — — — —

— Incr.;plasma cells : — 1 — — — — — — — —

— Dilation; sinusoid : — — — — — — — 1 — —

—————————————————————————————————————————————————————————————————————————

—————————————————————————————————————————————————————————————————————————

MEDIASTINAL TISSUE : 1 1 1 — — — — — — —

— Increase; brown fat : 1 — — — — — — — — —

—————————————————————————————————————————————————————————————————————————

MEDIAST. LYMPH NODE : 2 — 4 — — 1 2 — 3 —

— Dilatation; sinusoid: 1 — 1 — — — — — 2 —

— Hemorrhage : 2 — 4 — — 1 1 — 2 —

— Infilt;macrophage : 1 — — — — — — — — —

— Pigment; hemosiderin: 1 — — — — 1 1 — 1 —

—————————————————————————————————————————————————————————————————————————

LUMBAR LYMPH NODES : — — — — — — 1 — — —

— Dilatation; sinusoid: — — — — — — 1 — — —

— Erythrocytosis : — — — — — — 1 — — —

—————————————————————————————————————————————————————————————————————————

INGUINAL LYMPH NODES : — 2 — — — — — — — —

— Incr.;plasma cells : — 1 — — — — — — — —

—————————————————————————————————————————————————————————————————————————

BODY CAVITIES : — — 2 — — — 1 — — —

—————————————————————————————————————————————————————————————————————————

ABDOMINAL CAVITY : 5 7 8 10 5 6 6 7 7 3

— Fat necrosis : 4 7 7 9 4 5 4 5 5 2

— Infilt; mixed cell : — — — — — 1 — — — —

— Infilt;mononuc cell : — — — — — — — — 1 —

—————————————————————————————————————————————————————————————————————————

SALIVARY GLANDS : — — — — — — — — 1 —

—————————————————————————————————————————————————————————————————————————

LARYNX : — — — — 1 — — — — —

—————————————————————————————————————————————————————————————————————————

SMALL INTESTINE : — — — — — — — — — 1

—————————————————————————————————————————————————————————————————————————

DIAPHRAGM : — — 1 1 — — — — — —

—————————————————————————————————————————————————————————————————————————

neutro(ph), neutrophil; eosin, eosinophilic cell type; basoph, basophilic cell type; vacuol., vacuolated cell type; CL, centrilobular; hepatodiaph., hepatodiaphragmatic nodule; hyperpl, hyperplasia; hypert, hypertrophy; hepatoc, hepatocyte; dif(f), diffuse; infl., inflammatory; CL, centrilobular; hystioc, histiocyte; foc, focal; infilt, infiltrate; chron. prog. nephrop, chronic progressive nephropathy; urothel, urothelium; urothel. hyperpl, urothelial hyperplasia; inflamm, inflammation; G.A.L.T., gut associated lymphoid tissue; epithel, epithelial; L.R., limiting ridge; submuc, submucosal; ep(ithel), epithelium; Brunner’s gl, Brunner’s glands; alveol, alveolar cell (hyperplasia) or alveoli (inflammation); Leydig, Leydig cell; sperm'cele, spermatocele; mesoth, mesothelium; NOS, not otherwise specified; foc. granulos. hyperpl, focal granulosa cell hyperplasia; SCS, sex cord stromal; mononuc, mononuclear cell; extracaps cort tiss, extracapsular cortical tissue; subcaps, subcapsular cell; vacuol, vacuolation; salivary gl, salivary glands; granulomat, granulomatous; epiderm, epidermal; pit, pituitary neoplasm; cystic endom, cystic endometrial; endomet, endometrium; squam, squamous; pars int, pars interior; PA or p. ant. or pars anter, pars anterior; chondromucin., chondromucinous; incr., increased cellularity
